# A complete statistical model for calibration of RNA-seq counts using external spike-ins and maximum likelihood theory

**DOI:** 10.1371/journal.pcbi.1006794

**Published:** 2019-03-11

**Authors:** Rodoniki Athanasiadou, Benjamin Neymotin, Nathan Brandt, Wei Wang, Lionel Christiaen, David Gresham, Daniel Tranchina

**Affiliations:** 1 Center for Genomics and Systems Biology, Department of Biology, New York University, New York, New York, United States of America; 2 Center for Developmental Genetics, Department of Biology, New York University, New York, New York, United States of America; 3 Department of Biology, New York University, New York, New York, United States of America; 4 Courant Institute of Mathematical Sciences, New York University, New York, New York, United States of America; University of Michigan, UNITED STATES

## Abstract

A fundamental assumption, common to the vast majority of high-throughput transcriptome analyses, is that the expression of most genes is unchanged among samples and that total cellular RNA remains constant. As the number of analyzed experimental systems increases however, different independent studies demonstrate that this assumption is often violated. We present a calibration method using RNA spike-ins that allows for the measurement of absolute cellular abundance of RNA molecules. We apply the method to pooled RNA from cell populations of known sizes. For each transcript, we compute a nominal abundance that can be converted to absolute by dividing by a scale factor determined in separate experiments: the yield coefficient of the transcript relative to that of a reference spike-in measured with the same protocol. The method is derived by maximum likelihood theory in the context of a complete statistical model for sequencing counts contributed by cellular RNA and spike-ins. The counts are based on a sample from a fixed number of cells to which a fixed population of spike-in molecules has been added. We illustrate and evaluate the method with applications to two global expression data sets, one from the model eukaryote *Saccharomyces cerevisiae*, proliferating at different growth rates, and differentiating cardiopharyngeal cell lineages in the chordate *Ciona robusta*. We tested the method in a technical replicate dilution study, and in a *k*-fold validation study.

## Author summary

We present a complete statistical model for the analysis of RNA-seq data from a population of cells using external RNA spike-ins and a maximum-likelihood method for genome-wide estimation of transcripts per cell. The model includes biological variability of cellular transcript number and sampling noise. We derive an unbiased estimator of transcripts per cell for every transcript, given by simply multiplying the count by a library-dependent, but transcript-independent, scale factor. This is a nominal estimate that can be converted to an absolute estimate by dividing by the transcript’s relative yield coefficient, measured in a separate experiment. A negative binomial probability mass function with novel normalization (size) factors allows for parametric testing of hypotheses concerning dependence of the absolute abundance of each transcript on experimental condition. Our method integrates information from every RNA-seq experiment across all replicates and experimental conditions to determine the calibration constants. We test the method with a dilution study and a *k*-fold cross-validation study. We illustrate our method with applications to two independent data sets from yeast and the sea squirt that were derived by different library preparation protocols. We show that our methods detect genome-wide amplification of expression, and we compare our method to others.

## Introduction

Accurate transcriptome measurements are central to understanding the fundamental mechanisms of gene expression. A main challenge presented by the RNA-seq method for digitizing information about cellular RNA content—both its composition and abundance—is correcting noise, errors, and biases introduced in the process of making the measurement. An important step in typical library preparation for sequencing is random fragmentation of the molecules to be sequenced. The actual unit that is being digitized during RNA-seq is therefore not the RNA molecules directly, but their fragments, whose number depends on transcript length and RNA abundance. However, it is the molecular abundance of a transcript that is of interest, rather than the distribution of fragments over its gene model.

The introduction of Reads per Kilobase of exon model per Million mapped reads (RPKM) as the unit measurement by Mortazavi *et al*. [1] addressed this problem. The work of Mortazavi *et al*. [1] and of Tarazona *et al*. [2] both addressed the problem of varyng sample size (sequencing depth). Other researchers looked more carefully at fragment biases and developed a maximum likelihood algorithm to estimate the true differences [3]. The adoption of “read-counts” overlapping each transcript [4] instead of FPKMs (Fragments per Kilobase of exon model per Million mapped reads) [5] has allowed a more intuitive interpretation of the data.

Among the models that have been proposed for RNA-seq count data [6–9], the intuitively appealing negative binomial model [10] has become the most popular. The negative binomial probability mass function can be thought of as a mixture distribution arising from concatenating biological noise in transcript abundance (described by a gamma probability density function) and sampling noise (described by a Poisson distribution) in the compilation of corresponding sequencing counts. Applications of the negative binomial distribution and methods of hypothesis testing have been reviewed recently in [11]. Widely used normalization, statistical modeling, and hypothesis-testing methods are implemented in widely used R packages, edgeR [12], EDASeq [13], and DESeq2 [14]. Methods that focus particularly on the removal of unwanted variation from unspecified extraneous, nuisance sources are implemented in the R package RUVSeq [15]. A common assumption among all these methods is that, while the proportions of some transcripts vary across conditions/treatments, most transcripts do not vary between experimental conditions, and the total abundance of cellular RNA remain more-or-less fixed.

Lovén *et al*. [16] were the first to demonstrate an experimental system in which the central assumption of transcriptome equivalence among conditions is not satisfied. The researchers discovered that in cells overexpressing the oncogene cMyc, 90% of all transcripts are also overexpressed. To overcome the problem and allow comparison of expression levels between normal and cMyc overexpressing cells, the group incorporated external spike-ins in their samples, which were then used as a *de facto* invariant pool of RNAs. The spike-in approach had been previously used successfully in microarray experiments and its use in RNA-seq was facilitated by the development of external RNA spike-in mixes by the External RNA Controls Consortium (ERCC) [17, 18]. The need to normalize high-throughput RNA and DNA counts, in general, by the use of spike-in standards was recently explained and validated in the wide-sweeping paper of [19]. Even more recently, external RNA spike-ins were used in single cell RNA-seq experiments [20].

The ERCC external RNA spike-in mix 1 (Ambion) that was used in this study, consists of 92 different synthetic RNAs at 22 different concentrations spanning six orders of magnitude (30,000–0.01 amol/*μ*L). Instead of relying on normalization methods that aim to match the cellular and spike-in RNA read-count distributions, we take advantage of the digital nature of the RNA-seq output and we use the spike-ins as calibrators of known absolute abundance in the samples. We demonstrate that our calibration/normalization model is applicable in two different model organisms (the unicellular eukaryote *Saccharomyces cerevisiae* and the multicellular chordate *Ciona robusta*, three experimental setups, a growth rate regulation and a dilution study in yeast, as well as an embryonic differentiation and cell lineage specification study in *Ciona*), and two library preparation protocols. We perform hypothesis testing and detect global amplification of gene expression in both organisms.

## Materials and methods

### Experimental design

We used three distinct experimental setups, two representing different cases in which the assumption of constant transcriptome sizes is violated. We added a fixed, known amount of external RNA spike-ins to the sample of cells. In the rest of the paper, we refer to *spike-in abundance per cell* to mean the ratio of spike-in amount (molecules) added to the sample divided by the number of cells in the sample.

#### Dilution experiments

We used 2 different volumes, 1.8 and 8 *μ*L of 1:10 dilution of our stock spike-in mixture added to the same quantity of cells. The low-volume aliquot was added to 3 of 6 technical RNA replicates, and a high-volume aliquot was added to the other 3. Thus, the spike in abundances were intended to be larger by a factor of 4.44 for the high-volume replicates compared to the low-volume replicates.

#### Growth rate experiments

For the growth rate (GR) datasets, we harvested ten million cells from asynchronous *Saccharomyces cerevisiae* cultures, with exponential growth rate constants of 0.12, 0.20 or 0.30 h^-1^. Experimental control of cell growth was achieved using chemostats [21, 22] in limiting concentrations of carbon [23]. We used RNA extracted from chemostat cultures growing at the three growth rates in biological triplicates.

#### Embryonic differentiation experiments

We used an *in vivo* cardiopharyngeal differentiation system in *Ciona robusta* [24, 25]. We measured the RNA abundance across two types of Trunk Ventral Cells (TVC) progeny, isolated from larvae 15 hours post fertilization (hpf), including the homogenous First Heart Progenitors (FHP)-like cell population from Fgfr^DN^ sample, the homogeneous Second TVC (STVC)-like cell population from M-Ras^CA^ sample, and the heterogeneous STVC+FHP cell population from control (LacZ) sample. These cell types are known to differ in size. Given the findings of [26] who found RNA abundance scales with cell volume in mammalian cells, we hypothesized that cells size differences in *Ciona* may results in differences in absolute mRNA abundance. We isolated RNA from 800 cells from this embryonic lineage in each one of the three experimental conditions, sorted by Fluorescence Activated Cell Sorting (FACS) as described in [27], in triplicate, for a total of nine libraries.

### RNA-seq library preparation and data preprocessing

In all libraries we avoided the use of poly-dT for reverse transcription as it has been previously shown to be incompatible with quantitation through external spike-ins for RNA-seq [28]. In all cases the filtered aligned reads were converted to counts using the function featureCounts from the package Rsubread (R, Bioconductor) and strand information.

GR samples and samples for the dilution study were prepared essentially as described in the Borodina *et al*. [29] directional RNA-seq protocol. We modified the protocol by using UMI adaptors [30, 31] that were used to eliminate PCR duplicates from the results. RNA was extracted from ten million cells after the addition of 2 *μ*l of 1:20 dilution of ERCC spike-in Mix 1 stock [32] in the lysis buffer. All samples were distributed in two lanes and sequenced on an Illumina HiSeq 2000, with 100 nt-long, single end reads. The RNA-seq data (fastq files) were first filtered for residual rRNA reads. The data were aligned to the latest version of the yeast genome (sacCer3) and spike-in sequences, and filtered for mapping quality using Bowtie with optimized parameters. The aligned reads were then processed with a custom script that removes PCR duplicates based on the combination of mapping coordinates and UMI adaptor barcodes.

For the samples of three different *in vivo* *Ciona* cell lineages, 800 cells were directly sorted into lysis buffer from RNAqueous-Micro Total RNA Isolation Kit (Ambion) containing a fixed amount of ERCC spike-in mix 1. Total RNA extraction was performed according to the manufacturer’s instructions, followed by depletion of rRNA in the samples. The quality and quantity of total RNA in all stages were measured using Agilent RNA 6000 Pico Kit (Agilent) on Agilent 2100 Bioanalyzer. cDNA were synthesized using the SMART-Seq v4 Ultra Low Input RNA Kit (Clontech). RNA-Seq Libraries were prepared and barcoded using Ovation Ultralow System V2 1-16 (NuGen). The samples were pooled in one lane and sequenced on an Illumina HiSeq 2500, with 50 nt-long, single end reads. The RNA-seq reads were mapped to the *Ciona* genome (v.2008, ghost database) using Tophat2 with default parameters. The mapped reads were assigned to *Ciona* KH gene models (v.2013).

### Computational methods

All computer programming, including data analysis, Monte Carlo simulations, hypothesis testing, and figure preparation were done in the R programming language and environment. For pairwise testing for differential gene expression, we used DESeq function in the DESeq2 package [14]. In some cases hypothesis testing followed the application of an RUV normalization method in a suite of 3 R functions, RUVr, RUVs and RUVg, in the RUVseq package [15]. For maximum likelihood estimation of parameters we used the R function nlm, a general R function that minimizes a supplied objective function over its parameters. We used it to minimize minus log likelihood of the observed data in the context of a model. The first argument of the nlm function is the name of the name of the R function that computes minus log likelihood for the problem at hand. We wrote such a function for each maximum likelihood problem we considered.

## Results

All symbols, variables, parameters, and statistics used throughout this paper are listed in Table 1.

**Table 1:** Symbols and definitions.

Symbol	Definition
*i*	RNA index: *i* = 1, 2, …, *s* for spike-in molecules (*s* = 92); *i* > 92 for native RNA Index for sample or library
*l*	index for experimental condition or cell type: e.g., *l* = 1, 2, 3 for C-limited growth at 0.12, 0.20, and 0.30 h^−1^, respectively
Ω_*l*_	Set of indices for experimental condition: e.g., Ω_1_ = {1, 2, 3}
$L_{j}^{\text{SI}}$	Counts for spike-ins in library *j*
$L_{j}^{\text{tot}}$	Counts for all molecules (RNA and spike-ins) in library *j*
$L_{j}^{\text{RNA}}$	Counts for transcripts only in library *j*
*Y*_*i*,*j*_	Random count (from a population perspective) for molecule *i* in library *j*
*y*_*i*,*j*_	Observed count for molecule *i* in library *j*
*n*_*i*,*j*_	Abundance (amol) of spike-in molecule *i* in sample *j*, from which library *j* is derived, independent of *j*, except for the technical-replicate libraries
*f*_*i*_	Fraction of total spike-in counts across replicates contributed by spike-in molecule *i*
*α*_*i*_	Relative yield (dimensionless) for spike-in or transcript *i*; i.e. expected counts per attomole divided by that of a reference spike-in. Note that *α*_*i*_ is molecule-dependent but independent of library *j*
*N*_*i*,*j*_	Random amol of transcript *i*, for *i* > *s*, in sample *j*
*Z*_*i*,*j*_	*α*_*i*_ *N*_*i*,*j*_, referred to as nominal abundance or abundance for short
*ν*_*j*_	Calibration constant for converting count *y*_*i*,*j*_ to abundance *z*_*i*,*j*_
*a*	Shape (dispersion) parameter in negative binomial/gamma probability mass/density function.
NB(*μ*, a)	Negative binomial random variable with mean *μ* and shape parameter *a*
NB(y; *μ*, a)	Negative binomial probability mass function evaluated at *y*.
*γ*	Growth rate.
*δ*_*j*_	Library *j* correction factor for unwanted variation.
*ϕ*	Vector of parameters, (*ϕ*_0_, *ϕ*_1_), in exponential function for *μ* as a function of *γ*.
*f*_*Z*_(*z*; *i*, *j*)	Probability density function (pdf) of random (from a population perspective) amol of RNA transcript *i* in replicate *j*
∼	Distributed as; e.g. X∼N(0,1): *X* is distributed as a normal random variable with mean 0 and variance 1.
*χ*^2^(*n*)	Chi-squared random variable with *n* degrees of freedom.

### A mathematical and statistical framework linking sequencing counts to spike-in and cellular RNA abundance

**Fig 1:**
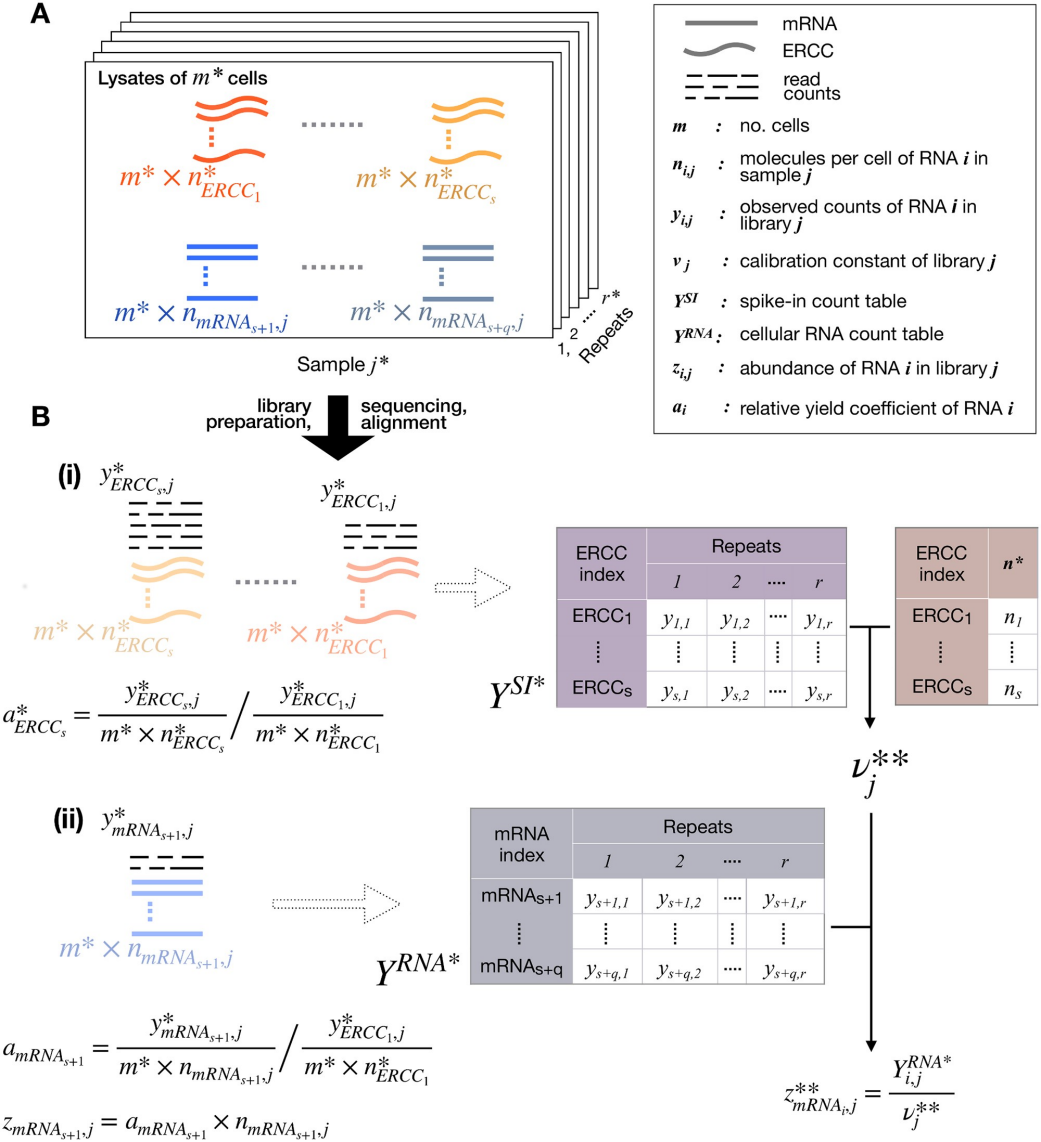
Diagrammatic summarization of the approach. Asterix (*) on a variable or constant quantity means its is known by design or can be measured/calculated. Double asterisk (**) on a variable means its value is an estimate defined in this study. Index *i* = 1…*s*, denotes a spike-in, while *i* = *s* + 1…*s* + *q*, cellular RNA. For clarity in the diagram, these indices have been given the notation *ERCC*_1…*s*_ and *mRNA*_*s* + 1…*s*+*q*_. The remaining mathematical notations in this figure follows exactly that of Table 1. **(A)** A fixed amount of spike-in RNA is added in fresh lysates from *m* cells in *r* repeats. The quantity of added spike-ins is known, and we want to calculate the quantity of endogenous mRNAs. **(B)** RNA is extracted from the lysates, RNA-seq libraries are prepared using a multi-step protocol, sequenced, aligned and count tables are constructed for spike-ins **(i)** and cellular RNA **(ii)**. We use the spike-in count table together with the vector of spike-in abundance to estimate the library calibration factor *ν*, which is in turn applied for the estimation of nominal abundance of endogenous RNA in the sample. The mathematical definition of relative yield (*α*), and nominal abundance (*z*) are also shown. Note that the definition of *z* as a function of *α* cannot be estimated (**) as neither *α*_*mRNA*_*s*+1__, nor *n*_*mRNA*_*s*+1_, *j*_ are known.

We use external RNA spike-ins as a calibration tool to normalize RNA-seq counts by introducing the variable relative yield (Fig 1). We parametrize a multinomial model for sampling noise, conditioned on native RNA abundances, with library size, relative yield coefficients, and known absolute abundances of spike-in molecules. In the context of this model, we derive a maximum likelihood estimation of nominal RNA abundance, proportional to absolute abundance, for each endogenous transcript in our sample. In the remainder of this paper, we often omit the qualifier “nominal” when we mean nominal abundance, and use the phrase “absolute abundance” when we mean molecules or attomoles (per cell or per sample).

In S1 Appendix, we show that the maximum likelihood estimator of RNA abundance is a library dependent scaling of counts by a factor that is proportional to total spike-in counts; it also depends on the known abundance of a reference spike-in, and fraction of overall spike-in counts contributed by this reference spike-in.

The expected proportion of counts for a given molecule (native RNA or spike-in) *i* represented in library *j* depends on the product of its abundance (attomoles or molecules per cell) in the original sample *n*_*i*,*j*_ and its *relative yield coefficient* *α*_*i*_; (Fig 1B(ii)). For spike-in molecules, *n*_*i*,*j*_ are known. We define the relative yield coefficient of a molecule (spike-in or RNA) to be the ratio of its yield coefficient to that of a reference spike-in. By yield coefficient of a molecule we mean the expected number of fragments per molecule contributed by that molecule to a total RNA-seq library of fixed size and prepared according to a fixed protocol. The relative yield coefficient captures specific properties of an RNA molecule such as transcript length and GC content. By convention, we assign the index 1 to the reference spike-in; consequently *α*_1_ = 1, by definition (Fig 1B(i)). For the sake of generality, we do not assume that relative yield coefficient is proportional to transcript length as in [33]. The relative yield coefficient of a spike-in is related to its FPKM within an RNA-seq spike-in library as follows. The relative yield coefficient multiplied by abundance in the original sample, and divided by length is proportional to FPKM.

In S1 Appendix, we express the expected proportion of counts for each molecule, indexed by *i*, in library *j* in terms of all *z*_*i*,*j*_ = *α*_*i*_*n*_*i*,*j*_, in a multinomial joint distribution of counts, and then solve for the maximum likelihood estimator of *z*_*i*,*j*_. Because we do not estimate the relative yield coefficients, *α*_*i*_ for cellular RNA molecules in the present paper, we cannot disentangle here their relative yield coefficients *α*_*i*_ and absolute molecular abundances, *n*_*i*,*j*_ (see Discussion). Consequently, we refer to *z*_*i*,*j*_ as a nominal abundance. The corresponding terms for spike-in molecules do not depend on library index *j* when the same amounts of spike-ins are used in each sample; so, for spike-ins, we can write more simply *z*_*i*_ = *α*_*i*_*n*_*i*_. For sake of clarity, we mention that, by our convention, the indices for the *s* spike-in molecules are *i* = 1, 2, …, *s*, and the molecule indices for the *q* detected native RNA molecules are *i* = *s* + 1, *s* + 2, …, *s* + *q*.

As shown in S1 Appendix, the derived maximum likelihood values of abundances, *z*_*i*,*j*_ for the RNA molecule *i* in library *j* are given by

$$\begin{array}{c} z_{i,j}\;=\;\frac{y_{i,j}}{\nu_{j}}\;\;\text{for}\;i=s+1,\;s+2,\ldots ,s+q,\;\text{and}\;j=1,2,\ldots ,r \end{array}$$ (1)

where *y*_*i*,*j*_ is the count (sequencing reads for RNA molecule *i* in library *j* and *ν*_*j*_ is the maximum likelihood calibration constant for library *j*. The calibration constant *ν*_*j*_ is given by

$$\nu_{j}\;\overset{\text{def}}{=}\;\frac{f_{1}L_{j}^{\text{SI}}}{n_{1}},$$ (2)

where *f*_1_ is the proportion of spike-in counts across all libraries contributed by the reference spike-in, *n*_1_ is the attomoles or molecules per cell, depending on one’s choice of units, for the reference spike-in, and $L_{j}^{\text{SI}}$ is the size (total counts) of spike-in library *j*.

In S1 Appendix we extend the estimation of *z*_*i*,*j*_ to a full statistical model, including biological variation, linking cellular RNA abundance to RNA-seq counts.

The *ν*_*j*_ calibration constant in Eq (1) is qualitatively like the dimensionless “technical” (library) size factor *s*_*j*_ of [34], but with an explicit relationship to absolute abundance, because 1/*ν*_*j*_ is on the scale of attomoles or molecules per cell. The relationship between the two factors is discussed thoroughly in S8 Appendix. The numerator on the right-hand side of Eq (2), according to our statistical model, is the expected number of counts from the reference spike-in, in replicate *j*, given the spike-in library size $L_{j}^{\text{SI}}$. As shown in S2 Fig, the “expected” counts given by the model for the reference spike-in, closely approximate the actual number. Therefore, Eq (1) says that the inferred abundance of RNA transcript *i* in replicate *j* is given by the counts for this transcript multiplied by a scale factor that is the attomoles or molecules per cell, per count of the reference spike-in. If it were known that, in fact, RNA transcript *i* on average yields twice as many aligned counts per amol as the reference spike-in, the abundance of RNA transcript *i* in the 10^7^ cells (from which our sample came) would be given by *z*_*i*,*j*_/2.

The normalization in Eq (2) is reminiscent of RPM (reads per million mapped reads) normalization, but the denominator involves the total number of reads in the spike-in library only. It makes intuitive sense, because read depth scales spike-in counts and endogenous RNA counts the same way [17]. Therefore, dividing an endogenous RNA count by spike-in counts derived from a fixed number of molecules in the original biological sample simultaneously normalizes for read depth and provides a measure proportional to the molecular abundance of the endogenous RNA in question. [17] applied this sort of normalization to ERCC spike-in counts, but also divided by spike-in length to obtain FPKM (fragments per kilobase per million mapped reads). Under the assumption that count scales with molecular length, FPKM would be proportional to spike-in abundance, in the absence of any molecular biases, e.g., GC content (see Fig 2 in [17]).

As shown in S1 Appendix, the derived maximum likelihood values of abundances for the *s* spike-in molecules are given by

$$\begin{array}{c} z_{i}\;=\;\frac{n_{1}}{f_{1}}\;f_{i}\;\;\text{for}\;i=1,\ldots s, \end{array}$$ (3)

where *f*_*i*_ is the empirical fraction of total spike-in counts, across all libraries, that is accounted for by spike-in molecule *i*, and *f*_1_ is that of the reference spike-in.

Because the absolute abundances of the spike-ins, *n*_*i*_ are known, Eq (3) and the definition *z*_*i*_ = *α*_*i*_*n*_*i*_, allows us to estimate the spike-in relative yield coefficients as

$$\begin{array}{ll} \alpha_{i} & =\frac{z_{i}}{n_{i}}\\ & =\frac{n_{1}}{n_{i}}\frac{f_{i}}{f_{1}} \end{array}$$ (4)

We chose as the reference spike-in the one that contributes the largest fraction of overall spike-in counts. Any of the top few spike-ins would do just as well.

We compute the spike-in relative yield coefficients, *α*_*i*_, describe their statistical properties and model the spike-in relative yield coefficients in terms of their biophysical properties in S6 Appendix. However, we do not yet have a large enough repertoire of spike-in molecules nor accurate enough biophysical models to use the model relative yield coefficients for spike-ins to estimate those of native RNA molecules. Thus, the computed spike-in *α*_*i*_ terms in the present paper are not participating in the estimation of native RNA abundances. In the Discussion we analyze how *α*_*i*_ could be used in the estimation approach.

### Evaluation of calibrations and treatment of variation within conditions

Following the recommendation of [35], we prepared diagnostic relative-log-expression (RLE) plots for all three of our data sets (dilution study, yeast GR study, *Ciona* embryonic differentiation study) to help in evaluating our maximum likelihood (*ν*_*j*_) calibration method. We found unwanted variation in the total inferred RNA abundance within conditions for all three data sets, and the variations are similar across data sets. We ascribe this variation to technical errors in one or more steps in the preparation of samples to be sequenced: variation in RNA extraction efficiency, error in cell count, dilution and/or volume errors in preparation of the spike-ins added to the cellular RNA. We refer to these errors collectively as library preparation errors. Details of results and analyses are presented in S1 Fig and S2 Appendix. Accordingly, we derived a library-specific scale factor, *δ*_*j*_ (S2 Appendix), much like the total RNA correction factor, *ξ*_*j*_, in the single-cell RNA-seq study of [36] (S8 Appendix). The corrected nominal abundance values are computed as *z*_*i*,*j*_/*δ*_*j*_, and we performed statistical analyses and hypothesis testing on these corrected values. In S8 Appendix we discuss, and in S5 Fig. we illustrate, the similarities and differences of this noise reduction to a removal of unwanted variation (RUV) method RUVr in the RUVSeq R package [15] that is based on residuals in a generalized linear model.

#### K-fold cross-validation of calibration method

In our model, spike-in counts and those from cellular RNA are treated similarly for given abundances of underlying spike-ins and RNA. They are on the same footing in that the joint distribution of spike-in and RNA sequencing reads, given the molecular abundances in the sample, is assumed to be multinomial. An important difference is that we know that the absolute abundance of each spike-in molecule is fixed across samples. This knowledge allowed us to to compute a scaling factor that converts sequencing reads to corresponding (nominal) attomoles for each native RNA transcript in each library.

The multinomial model for spike-in counts is an oversimplification, because it does not take into account overdispersion that was characterized by [17, 36]; this is illustrated in S2 Fig and discussed in S5 Appendix. Therefore, we provide an additional practical test of our method by *training* our model on libraries from all but one condition in a study and using this resulting model to estimate absolute abundance of spike-in molecule (units of molecules per cell), when they are treated as native RNA, in each of the leave-out conditions. It is important to note, as long as the spike-in counts adhere closely to the multinomial model, performance in the k-fold cross-validation study is guaranteed to be good; so this study is a way of quantifying the extent to which the multinomial model provides an adequate description of spike-in statistics regardless of the cellular RNA background. Extrapolation of the results here to infer that our method also provides accurate estimates of true cellular RNA abundance is based on the assumption that the RNA sequencing reads also adhere to the multinomial model, at least approximately, for given RNA abundances. This includes the assumption that the relative yields of the RNA molecules are fixed for a fixed protocol. Details of our k-fold cross-validation method and our quantification of error by mean fold error are provided in S3 Appendix.

**Fig 2:**
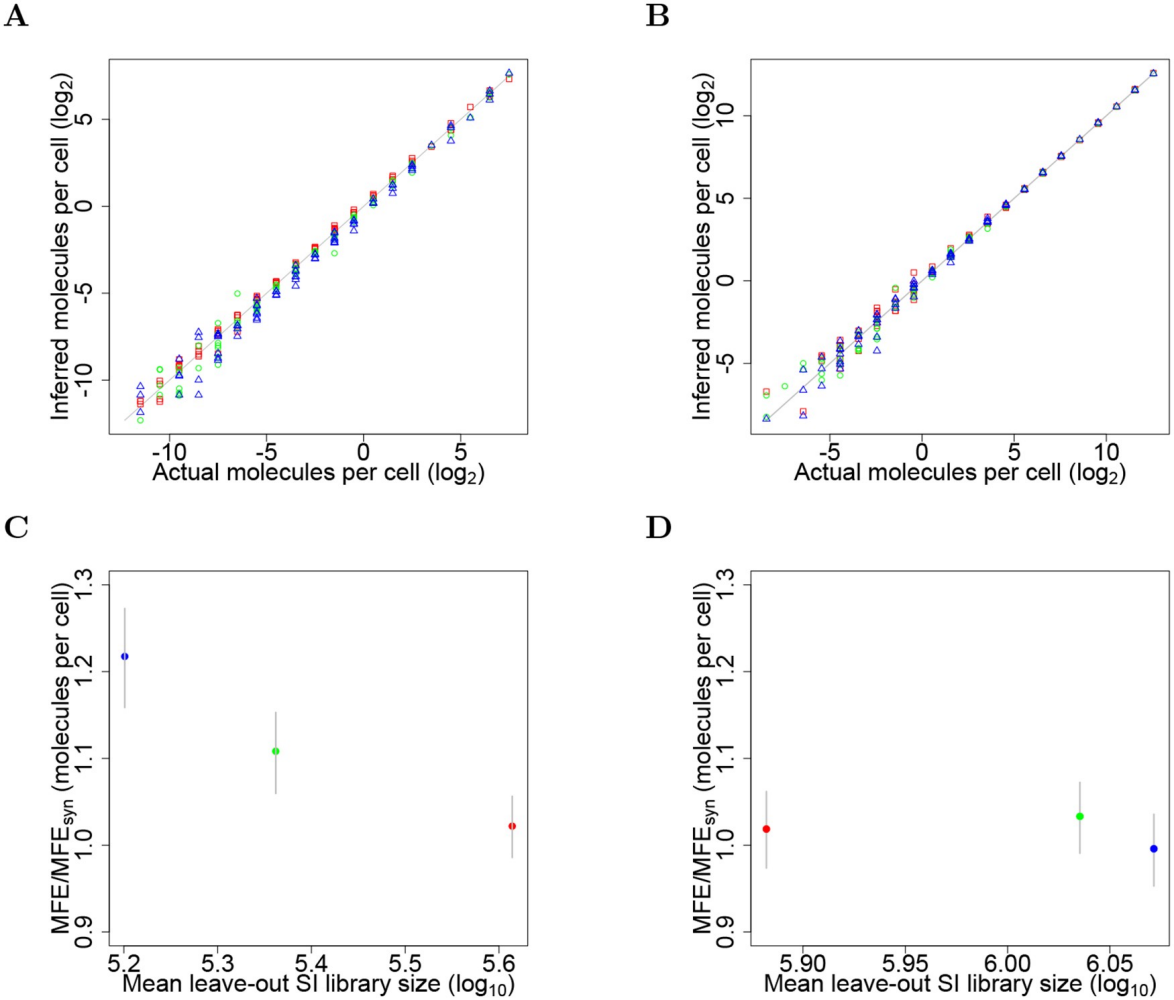
k-fold cross-validation: Inferred vs. actual molecules per cell of spike-ins. **(A)** GR experiment data. Inferred (mean) values vs. actual values are plotted (symbols) for each spike-in molecule in each of 3 leave-out conditions: carbon-limited growth at rates of 0.12 (red), 0.20 (green) and 0.30 h^-1^ (blue). **(B)** *Ciona* lineage specification data. Each symbol corresponds to the inferred value in each of 3 leave-out conditions: LacZ (red), Fgfr^DN^ (green), and M-Ras^CA^ (blue). Although in (A) and (B) each leave-out condition is plotted with a distinct symbol, a symbol can appear multiple times for some values along the x-axis, because these values are represented by several different spike-ins; i.e., among the 92 spike-in molecule there are 22 unique abundance values. **(C)** Measure of performance in three-fold cross-validation in (A). Mean Fold Error (MFE) is computed between inferred and actual molecules per cell. Symbols plot the average value, over 10,00 Monte Carlo trials, of the ratio MFE/MFE_syn_ versus the mean spike-in library size in the leave-out condition. Vertical bars span the mid 0.95 quantiles of MFE/MFE_syn_ values obtained in 10,000 MC trials for each leave-out condition. **(D)** Measure of performance in three-fold cross-validation study in (B) for the *Ciona* data.

Fig 2A shows results of a three-fold cross-validation study based on data from the GR study. We *trained* the model on spike-in counts from 2 conditions (at 3 replicates each). We tested the model by predicting molecules per cell for each spike-in molecule in the leave-out condition (average over 3 libraries in the leave-out condition). Inferred (predicted) spike-in molecules per cell in a leave-out condition is plotted versus the actual molecules per cell. In this three-fold cross-validation, inferences for each molecule were made successively for each of the 3 possible leave-out conditions (red squares, green circles, and blue triangles, respectively). The solid line plots inferred equals actual abundance. Fig 2A shows good agreement between inferred and actual abundance, for all leave-out conditions (growth rates), over a 2.1 × 10^6^-fold range. Fig 2B shows results of a similar three-fold cross-validation based on spike-in data from study of differential RNA abundance in 3 experimental conditions in *Ciona*.

We objectively verify the seemingly good performance for all leave-out conditions, that span over a 2.1 × 10^6^-fold range of abundances (Fig 2C and 2D). We compared the Mean Fold Error (MFE) values for the laboratory data, where the mean is over spike-ins, to those obtained from synthetic data, MFE_syn_ values, for 10,000 Monte Carlo trials in which synthetic counts were generated for all spike-in libraries at once according to the multinomial model. Panels C and D quantify the error in panels A and B, respectively. Fig 2C plots the average value over MC trials of MFE_*l*_/MFE_*l*,syn_ (symbols) for each leave-out condition *l* as a function of the mean spike-in library sizes for the leave-out condition. Perfect agreement between inferred and actual abundance per cell, across all spike-ins in the leave condition would give a MFE ratio of 1. Fig 2D for *Ciona*, with all spike-in library sizes exceeding those for yeast in panel C, shows MFE ratios less than 1.04 for all leave-out libraries. The 2 largest libraries for the GR experiment (panel C) gave ratios roughly equal to 1.1.

Our conclusion is that the population of spike-in molecules, spanning a 2.1 × 10^6^-fold range of abundance, behave in a reliable manner, with respect to inferred average abundances, and that the multinomial model, despite its flaws, is serviceable, provided that the total size of the spike-in library exceeds roughly 250,000 reads.

It is important to note that the approximate adherence of spike-in counts to the multinomial model, for the larger library sizes, does not seem to be affected by the abundance of native RNA. For example, the abundance of native RNA, as judged by the ratio of total endogenous RNA counts to total spike-in counts, varies over a 2.8-fold range among the libraries that underlie the data points in Fig 2C. A similar computation of the range of RNA abundance in the *Ciona* data (Fig 2B and 2D) gives a 2.4-fold range.

### Validation using a dilution study

**Fig 3:**
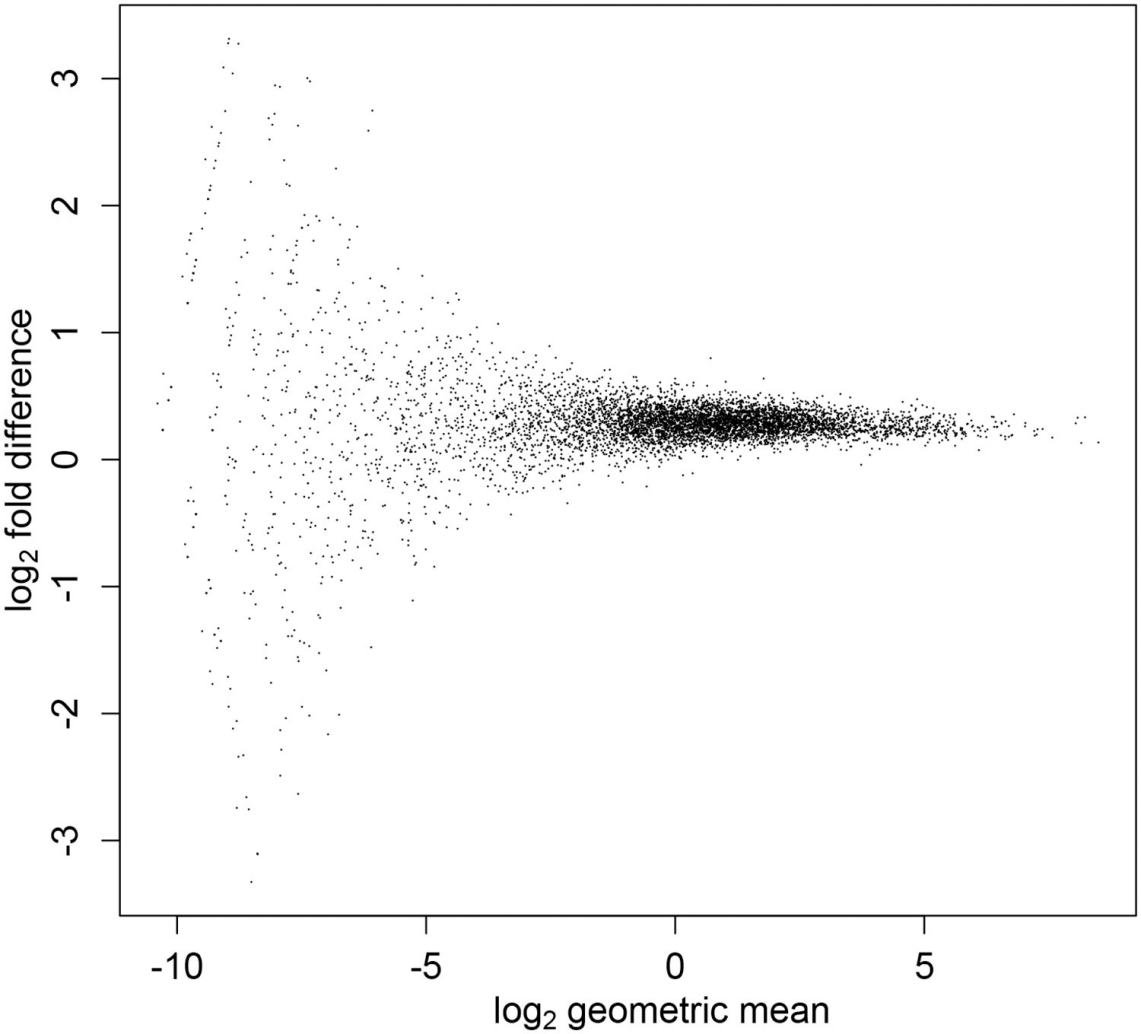
MA plot for dilution study. MA plot for mean RNA abundance (*z*-values) for libraries prepared with high- and low-dilution spike-in aliquots. The abundance *z*_*i*,*j*_ corresponding to count *y*_*i*,*j*_ was obtained by the maximum likelihood normalization *z*_*i*,*j*_ = *y*_*i*,*j*_/*ν*_*j*_ in Eq (2). The ordinates of the scatter plot (one point for each transcript) should be centered around zero, which corresponds to equal inferred transcript abundance for libraries prepared with high- and low-dilution spike-in aliquots.

For this dilution study we prepared 3 replicate libraries with a “high” spike-in aliquot dilution, and 3 with a“low” dilution as described in Materials and Methods. In the absence of any library preparation noise, the molar amount of spike-ins are 4.44 times larger in samples that were added to low dilution spike-in aliquots compared to those with the high-dilution aliquots. The generalization of the *ν*_*j*_ normalization in this case is to simply add the subscript *j* (indexing library) to *n*_ref_ to give $\nu_{j}=f_{1}L_{j}^{\text{SI}}/n_{1,j}$. Perfect performance of our method, would give identical mean abundances for libraries prepared with the high- and low-volume aliquots. In Fig 3 the MA plot shows less than ideal performance in that the ordinates in the scatter plot are offset a bit (by 0.28) from zero. This corresponds to a mean fold difference of 1.2 rather than 1. This discrepancy might be due, at least in part, to sample preparation handling errors.

### Statistical model for cellular RNA and corresponding sequencing reads

Modeling RNA abundances within a condition as gamma-distributed random variables results in counts with the familiar negative binomial distribution, as shown in S1 Appendix. Previous applications of the negative binomial distribution for modeling RNA sequencing counts and various methods of hypothesis testing have recently been reviewed in [11]. Our model for sequencing counts is formally equivalent to that in [34], but with an important distinction. The library-specific size factor of [34] (based on genes), written according to our notation, is

$$\begin{array}{c} s_{j}\;=\;\underset{i}{\mathrm{median}}\frac{y_{i,j}}{{\left(\prod_{k=1}^{r}y_{i,k}\right)}^{1/r}}. \end{array}$$ (5)

The size factors *s*_*j*_ is a dimensionless constant that carries with it an implicit assumption of fixed total amount of cellular RNA. Furthermore, in practice, the median of *s*_*j*_ across libraries is of order 1. In contrast, although our calibration constants *ν*_*j*_ can be thought of as “size factors,” they are proportional to the total spike-in library size $L_{j}^{\text{SI}}$ in Eq (2), and 1/*ν*_*j*_ has dimensions of attomoles, or molecules per cell, depending on the units one chooses to use for the spike-in abundances *n*_*i*_ (*i* ∈ {1, 2, …*s*}).

Our *ν*_*j*_ calibration factor is closely related to the extension of [34], by [36] to estimate cellular RNA abundance with the use of a “technical size factor” resembling that in Eq (5), but computed using spike-in counts only. Hypothesis testing methods currently in the literature that are based on the negative binomial distribution of counts with library size factors *s*_*j*_, such as DESeq [34], can be used following our *ν*_*j*_ normalization in the manner described in S4 Appendix.

### Hypothesis testing

#### I(a). Dependence of RNA abundance on growth rate in yeast

Growth rate (GR) changes result in co-directional changes in total cellular RNA content in both prokaryotic and eukaryotic cells. Previous studies found that changes in the total abundance of rRNA and tRNA contribute to the GR dependent changes in RNA content in yeast cells [37, 38]. Our maximum likelihood *ν*_*j*_ calibration method clearly verifies these earlier observations and provides further insights of a transcriptome-wide up-regulation of total RNA abundance with increasing growth rate in the yeast GR study as described in detail below.

We found that the dependence of inferred RNA abundance on growth rate, *γ*, was well described by an exponential function

$$\begin{array}{c} \exp(\phi_{0}+\phi_{1}\left[\gamma -\bar{\gamma }\right]), \end{array}$$ (6)

where *ϕ*_0_ and *ϕ*_1_ are parameters to be fitted, for our particular set of growth rates: 0.12, 0.20, and 0.30 h^-1^. This function is not meant for extrapolation outside our experimental range of growth rates. For extrapolation outside this range of growth rates, a Hill-type function, which has a saturation point, might make more biological sense, but the curvature of the RNA abundance response to growth rate would require at least 3 parameters for such a function to be consistent with our data. Technical details for hypothesis testing in this GR study are given in S4 Appendix.

**Fig 4:**
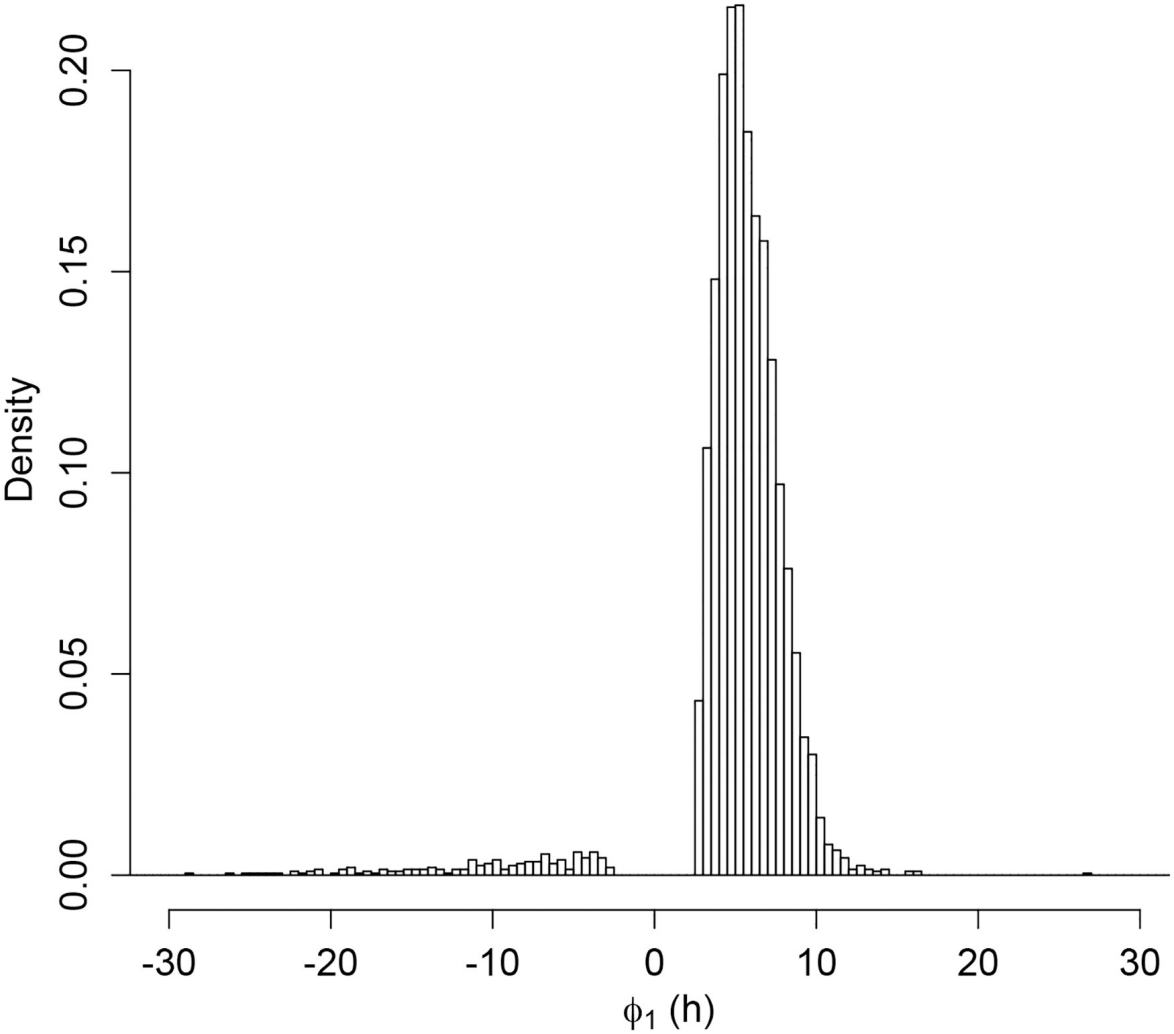
Exponential rate constants for exponential dependence of RNA abundance on growth rate. Histogram (density scale) of the exponential constant *ϕ*_1_-values in Eq (6), which describes the empirical exponential dependence of RNA abundance on growth rate. The histogram includes only those vales found to be statistically significant at an FDR of 0.01 [39].

We found 4,200 out of 7,468 detected transcripts to have statistically significant (empirical p-value) dependence of RNA abundance on growth rate (False Discovery Rate (FDR) cutoff, *q* = 0.01) In other words, a value of the exponential constant *ϕ*_1_ in Eq (6) significantly different form zero. The distribution of exponential constants is shown in Fig 4.

Of the significantly regulated transcripts, 95% were up-regulated (*ϕ*_1_ > 0 in Eq (6)) by increasing growth rate, and only 5%, down-regulated (*ϕ*_1_ < 0) (Fig 4). We found the geometric mean of the statistically significant positive exponential-constants to be 5.6. A *ϕ*_1_ value of 5.6 gives RNA abundance at the highest growth rate of 0.30 h^-1^ that is 2.7 times that at the lowest growth rate of 0.12 h^-1^. The geometric mean of the absolute value of negative exponential constants was 12, which corresponds to a down-regulation by a factor of 8.7 between growth rates of 0.30 and 0.12 h^-1^.

**Fig 5:**
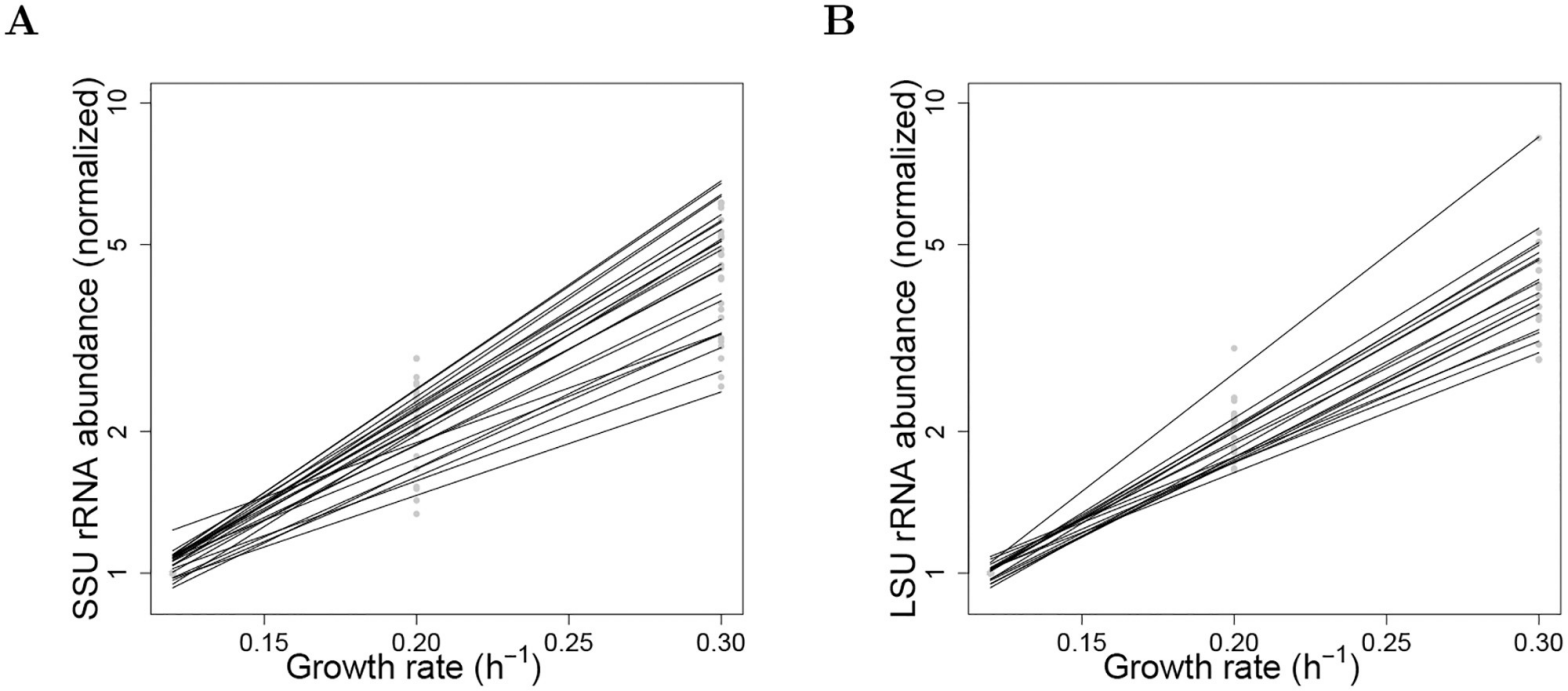
Up-regulation of ribosomal RNA molecules by growth rate. **(A)** Small subunit ribosomal RNA (SSUrRNA) molecules (GO:0015935). Normalized abundances (filled symbols) of significantly up-regulated SSUrRNAs, plotted as a function of growth rate on log-linear coordinates, and corresponding exponential-model values (solid lines), drawn from Eq (6), $\mu_{Z}\left(\gamma \right)\;=\;\exp(\phi_{0}+\phi_{1}\left[\gamma -\bar{\gamma }\right])$ where *γ* is growth rate. Each filled symbol at a given growth rate is the normalized mean over 3 replicates at that growth rate; the normalization factor is the mean at the lowest growth rate, 0.12 h^-1^. The determination of maximum likelihood parameters, *ϕ*_0_ and *ϕ*_1_, in Eq (6) was based on all replicates, so the model values (solid lines) are not constrained to go through the mean normalized value of 1 at the lowest growth rate. Mean ± sd for exponential constant *ϕ*_1_, 8.0±1.5. **(B)** Large subunit ribosomal RNA (LSU rRNA) molecules (GO:0015934). Normalized abundances of significantly up-regulated LSU rRNAs. Symbols and lines as in panel A. The total mean ± sd for exponential constant *ϕ*_1_ is 7.9±1.4.

In Fig 5 we demonstrate the transcript dependence on growth rate in yeast for individual transcripts. Abundance as a function of growth rate for small subunit ribosomal mRNAs (SSU rRNA) and large subunit ribosomal mRNAs (LSU rRNA) that were significantly up-regulated by growth rate are shown in Fig 5A and 5B, respectively. Of all 44 transcripts in both Gene Ontology (GO) groups, only one transcript, corresponding to one of a total 19 LSU rRNAs in the group, was not significantly upregulated.

Previously published results [37] have shown a dependence of rRNA abundance on growth rate in yeast. In this study we provide evidence that this is a more generalized phenomenon that extends to multiple RNA types, and our calibration method can generate sensible results in this context.

#### I(b). Consequences of a first round of global normalization that ignores spike-ins

**Fig 6:**
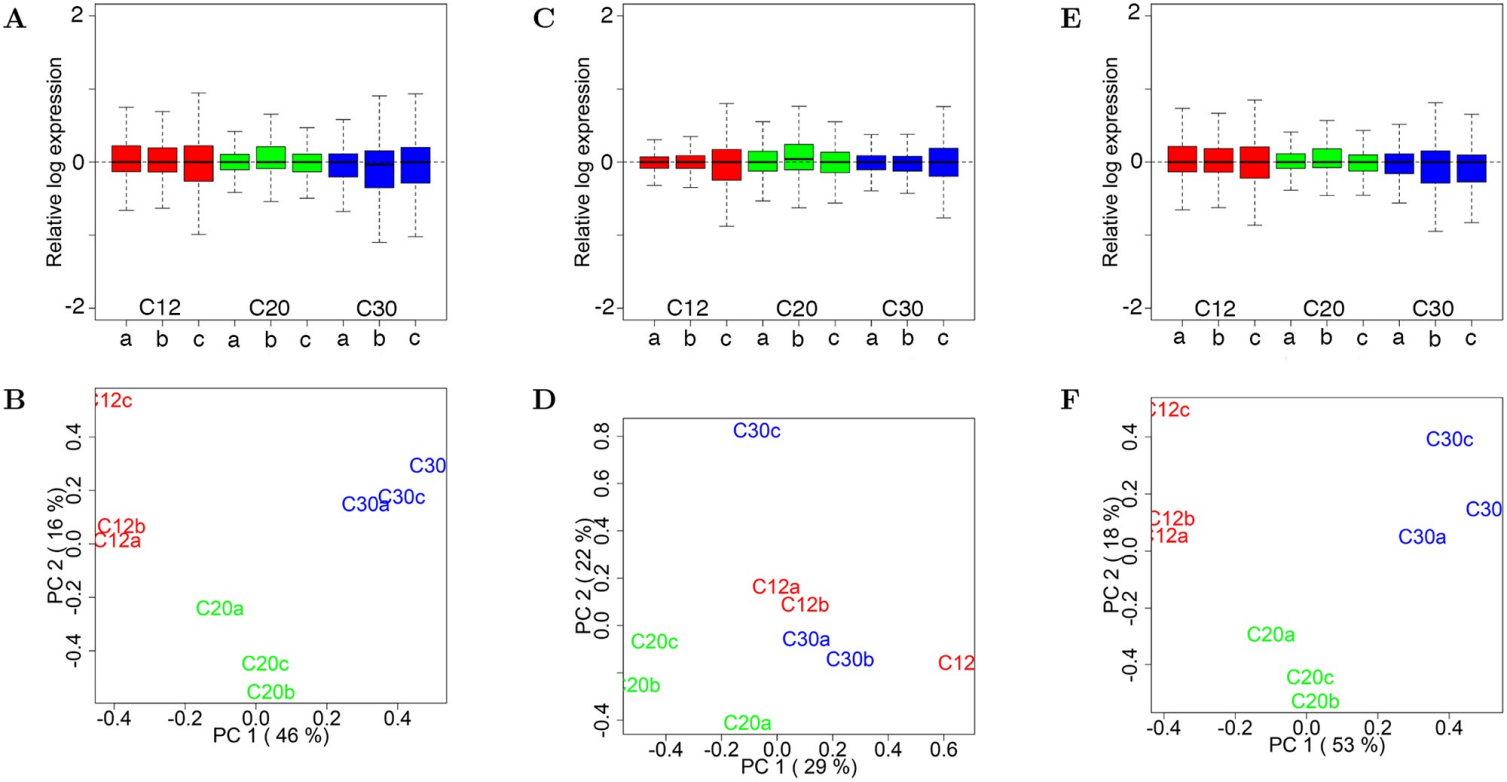
Global normalization in the first round ignoring spike-ins. **(A)** Based on data form the yeast growth rate study. Relative log expression (RLE) plots of raw counts normalized by median size factors [34]. The condition-dependent variation in the 0.5 quantile of log relative expression of S1D Fig has been largely eliminated. **(B)** PCA biplot corresponding to (A). **(C)** RLE plots of normalized counts produced by applying RUVg normalization [15], with one factor of unwanted variation, to the median-normalized counts in panels A and B. The ERCC spike-ins (same median global normalization applied to counts from cellular RNA). The RLE plots exhibit reduced variation of relative log expression within libraries compared to (A). **(D)** PCA biplot, corresponding to (C). The sensible clustering before RUVg normalization (B) has been disturbed. **(E)** RLE plots produced by applying a different RUV technique instead, RUVs [15], to the median-normalized counts in (A) and (B). Variation within libraries is somewhat reduced compared to that with median normalization alone in panel A. **(F)** PCA biplots corresponding to RLE plots in (E). These PCA plots are very similar to those in (A) for median normalization only. Pairwise testing for differential gene expression between growth rates of 0.30 and 0.12 h^-1^ gave very similar results for median normalization with and without RUVs normalization.

None of the classical global normalization methods that have implicit assumptions that only a small fraction of genes are differentially expressed and that the total cellular RNA is roughly constant across conditions (reviewed in [40]) provide an appropriate first normalization step when total cellular RNA abundance differs substantially across conditions. Such global normalization methods end-up obscuring genome-wide increases or decreases in RNA molecules per cell, when they exist. To emphasize this point, we demonstrate what happens in the analysis of our yeast GR data if one proceeds in a typical manner of an investigator unsuspecting of global amplification of expression. In this demonstration, we performed the first round of normalization with size factors *s*_*j*_ (median, based on the full set of genes and spike-ins) [34]. Fig 6A shows RLE plots of normalized counts after *s*_*j*_ [34] normalization. As we expect, condition-dependent variation in the 0.5 quantile of log relative expression that we see in S1 Fig has been almost entirely eliminated. To one unsuspecting global gene regulation, the only remarkable feature of the RLE plots in panel A is small variation within libraries and across conditions. A common exploratory tool for quality control is provided the PCA biplot of the normalized scores of the second, versus the first principal component for the log counts matrix, as shown in Fig 6B. This biplot suggests that the the libraries fall nicely into 3 groups, one for each condition (growth rate). This is confirmed by both hierarchical and kmeans clustering based on the entire scores matrix for the 9 principal components of the matrix of log transformed normalized counts.

Testing for differential gene expression between the lowest and highest growth rates (0.12 and 0.30 h^-1^, respectively) using the DESeq function in the DESeq2 R package [14] (with default *s*_*j*_ size factors) yields 1118 differentially expressed transcripts at and FDR of 0.01, half (566) up-regulated and half (552) down regulated. The symmetry between up- and down-regulated genes is characteristic of this misapplied normalization assumption. These results are incorrect and miss entirely the global gene amplification as judged by results with *ν*_*j*_ normalization method. However, to an investigator not anticipating global amplification of expression, the results are consistent with the notions that total RNA does not vary with condition, a small proportion of genes are regulated by conditions, and there is no preponderance for up-regulated or down-regulated genes.

Despite the seemingly *clean* results in Fig 6A and 6B, an unsuspecting investigator might seek to further reduce noise by applying the RUVg method [15] in a manner that uses the spike-ins as an invariant, control “gene” set. Fig 6C shows RLE plots of normalized counts produced by applying RUVg normalization, with one factor of unwanted variation, to the normalized counts in panel A. The RLE plot in Fig 6C exhibits reduced variation of relative log expression within libraries in general. However, the corresponding PCA biplot in Fig 6D shows that the relatively tight clustering before RUV normalization in panel B has been disturbed.

Following up RUVg normalization with testing for differential gene expression between the lowest and highest growth rates (including *W* and the original the design matrix (S8 Appendix, Eq (6)) yields only 44 differentially expressed transcripts at an FDR of 0.01. This is akin to the proverbial “throwing the baby out with the bath water” that [41] spoke about in their precautionary advice concerning the application of RUV methods. In this case, it simply reflects the fact that the normalization using spike-in as an invariant gene set does not capture well the sources of unwanted variation. The reason is that variation in the normalized spike-in counts are correlated with the biological variation in native RNA counts, driven in this experimental setup by growth rate. Fig 6E and 6F, show results of applying an alternative RUV normalization method, RUVs [15], instead, following median normalization. The RLE plots and PCA plots differ little form those produced by median (*s*_*j*_) normalization [34] only. Results of testing for differential gene expression are virtually the same with and without the second step of RUVs normalization. A conclusion is that the yeast GR data do not seem to exhibit any dramatic unwanted variation, and that the incorrect story the median-normalized counts tell is not altered for better or worse by application of RUV methods.

#### II. Differential gene expression in different embryonic cell types in *Ciona*

In our *Ciona* embryonic differentiation study, our interest is in detecting pair-wise differential gene expression among 3 cell types lacZ control, Fgfr^DN^, and M-Ras^CA^. Based on unpublished whole-mount *in situ* hybridization and single-cell sequencing data, we had reasons to suspect that FHP cells experience a global down-regulation response to termination of FGF-MAP*k* signaling (mimicked experimentally by the Fgfr^DN^ perturbation). Such a global effect is consistent with the literature on global transcriptome effects since FGF-MAP*k* acts downstream of *c-myc* [16]. The methodology for differential expression analysis in this study is detailed in S4 Appendix.

**Fig 7:**
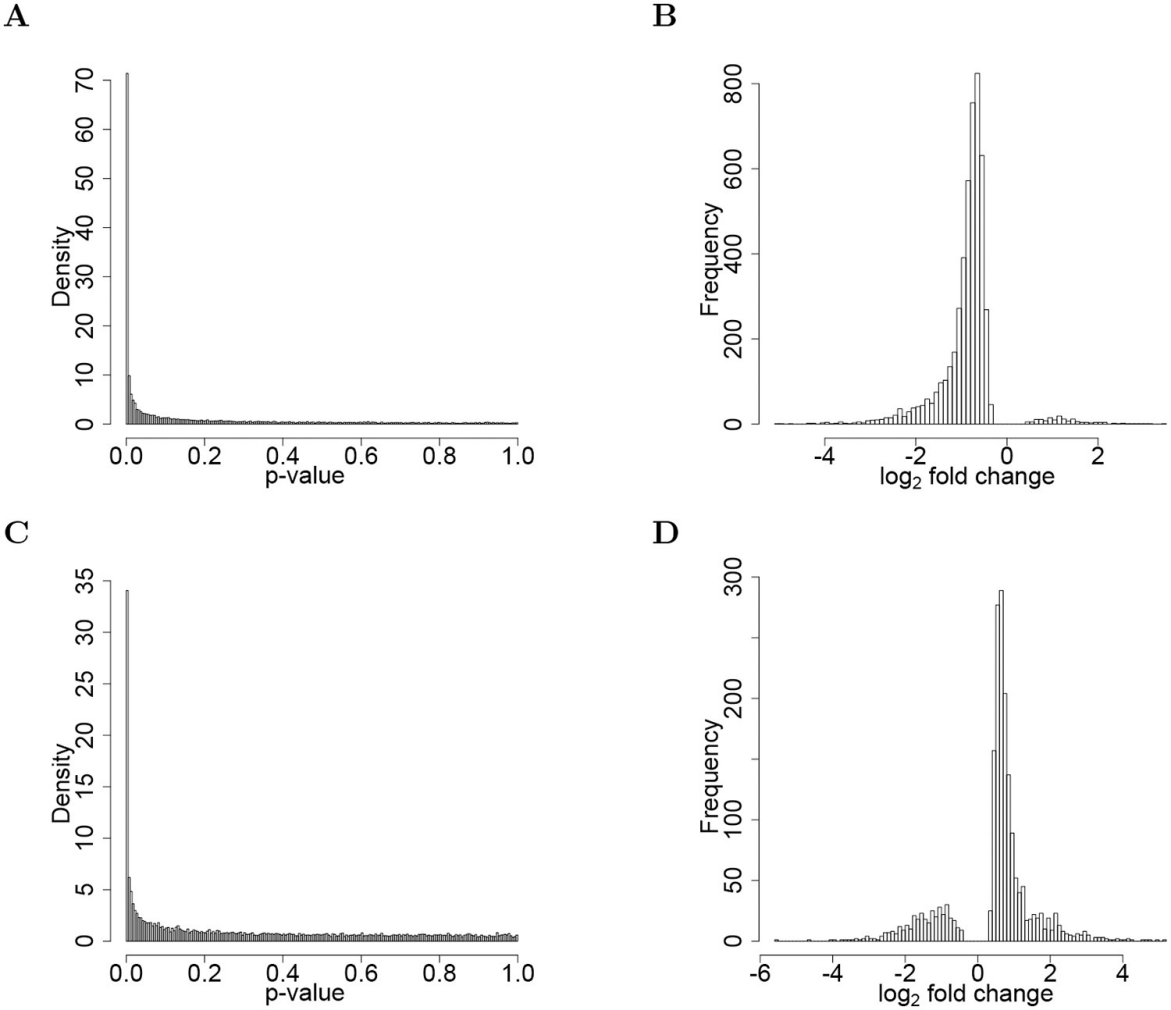
Differential gene expression in *Ciona* embryonic differentiation study. (A) Diagnostic histogram of p-values for null hypothesis of no differential gene expression between the Fgfr^DN^ and LacZ cell types. (B) Fold change (log_2_ scale) for the Fgfr^DN^/lacZ comparison. The vast majority of the significant (FDR = 0.01) transcripts were down-regulated in the Fgfr^DN^ cell type, 4,778 out of 4,493. The average fold change for up- and down-regulated transcripts was 2.5 and 1.9, respectively. (C) Similar to panel A, but for the null hypothesis of no differential gene expression between the Fgfr^DN^ and M-Ras^CA^ cell types. (D) Fold change, similar to panel B, but for the M-Ras^CA^/Fgfr^DN^ comparison. 1,934 transcripts differentially expressed at an FDR value of 0.01 (corresponding cutoff *p*-value equal to 0.0017). Of the 1,934 significant (FDR = 0.01) fold changes, 1,560 were greater than 1, and 374, less than 1. The average fold change for up- and down-regulated transcripts was 1.9 and 2.8, respectively.

We found dramatic pairwise abundance differences between the LacZ and Fgfr^DN^ cells types. For the FgfrDN/lacZ comparison in Fig 7A and 7B, the vast majority of the significant (FDR = 0.01) transcripts were down-regulated in the FgfrDN cell type, 4,778 out of 4,493. The average fold change for up- and down-regulated transcripts was 2.5 and 1.9, respectively with approximately one-third of the transcripts (4,934 out of the 15,078 detected transcripts) differentially expressed (FDR = 0.01, corresponding cutoff *p*-value equals 0.0038). For the M-Ras^CA^/Fgfr^DN^ comparison in Fig 7Ca and 7D, 1,934 transcripts were differentially expressed at an FDR value of 0.01 (corresponding cutoff *p*-value equal to 0.0017). Of the 1,934 significant (FDR = 0.01) fold changes, 1,560 were greater than 1, and 374, less than 1. The average fold change for up- and down-regulated transcripts was 1.9 and 2.8, respectively. We found essentially no differential gene expression between the LacZ and M-Ras^CA^ cell types. Only 17 and 50 transcripts were called differentially expressed at FDR values of 0.01 and 0.10, respectively.

These results corroborate our original observations, providing quantitative measures for the global down-regulation of gene expression in the Fgfr^DN^ perturbation.

## Discussion

In this paper, we described a detailed statistical model for cellular RNA and exogenous spike-ins in a sample prepared from a fixed number of cells to which a population of spike-in molecules of known numbers has been added. In the context of this model, we derived by maximum likelihood arguments, a calibration method for RNA-seq data that estimates cellular molecular abundance of RNA. Although our molecular abundance *z*-values are nominal, they are only one step away from absolute molecular abundance. Once the relative yield coefficient for transcript *i*, *α*_*i*_, is measured in separate experiments, the absolute molecular abundance in library *j*, *n*_*i*,*j*_ will be known via the equation: *n*_*i*,*j*_ = *z*_*i*,*j*_/*α*_*i*_.

Our method employs an explicit statistical model for spike-ins, the simplest sensible one, namely that the spike-in counts for a given library are sampled from a joint multinomial distribution with fixed proportion parameter for each spike-in molecule across all libraries/conditions for a fixed protocol. As a consequence, the counts within each spike-in library, regardless of *condition*, represent a technical replicate. We evaluated the spike-in model quantitatively in a number of ways (Fig 2 and S2 Fig). We found that the spike-in molecules adhere closely to the multinomial model provided the spike-in library exceeds roughly 250,000 reads. In other words, our results support those of [19]: the spike-in molecules contribute to spike-in counts within a spike-in library, embedded in an overall RNA-seq library, in a manner which is independent of the native RNA. A caveat is that we don’t know for sure if deviations of spike-in counts from the multinomial model that we observed are a consequence of some sort of poorly understood noise that is particularly prominent in spike-in libraries of low size, or if the unaccounted for noise was unrelated to library size per se.

We adopted a multinomial mode for spike-in noise, but our model could be extended with a more accurate model. Technical noise in spike-in counts has been studied and modeled recently [36], and we present similar analysis and modeling in S2 Fig and S5 Appendix. Although the proper experimental technique was followed in our study to minimize these errors, pipetting and dilution errors can not be completely eliminated. Pipetting, dilution, and cell number errors may have been sources contributing to the very high variation between experiments that was observed in previous attempts to incorporate spike-ins as normalization standards [42]. [20] however demonstrate technical robustness in the performance of spike-ins in sensitive single cell RNA-seq experiments. Our data agree with the assessment of [20].

We have shown however that our method, especially when supplemented with RUVr [15] correction or our own *δ*_*j*_ correction, is able to compensate for this source of unavoidable technical variability. Our model could be extended and improved in the future by incorporating a different model for spike-ins. Nevertheless, our model allows for powerful, genome-wide, parametric testing of hypotheses of various sorts concerning nominal RNA abundances, *z*-values that are explicitly related to absolute cellular molecular abundance (transcripts per cell or attomoles).

We applied our method, to quantify RNA abundance and to test for differential gene expression, using data from two studies with different library preparation protocols, and in species from different kingdoms: a growth rate study in yeast, and a low cell count differentiation study in *Ciona*. We found global changes in gene expression in both systems: a global increase in transcript abundance with growth rate in yeast, and a global decrease in the Fgfr^DN^ embryonic cell type in *Ciona*. Reanalysis of the raw data with other algorithms that hold the assumption of equivalent transcriptome sizes, as expected, were not able to reveal these global transcriptome trends.

### From relative yield coefficients to absolute cellular molecular abundance

Our focus in this paper is on deriving a nominal cellular molecular abundance that can be converted to absolute abundance by the transcript’s relative yield coefficient, which could be measured in separate experiments. In this study however, we do not attempt to measure the relative yield coefficient values, or estimate the absolute number of molecules per cell for each transcript within a condition. The current work allows us to say, that, for example, RNA transcript A has *x* times more molecules per cell, on average, in condition 1 compared to condition 2, even if the corresponding RNA-seq libraries were prepared in different batteries of experiments, different studies, or even prepared in different laboratories. Such a conclusion about what might be called, an absolute ratio of abundances, can be drawn without knowing the relative yield coefficient of transcript A. In the section that follows, we discuss the links between our work and methods by which these relative yield coefficients might be measured.

In this manuscript we offer RNA abundance estimates that are proportional to absolute transcript abundance. For this we assign a (relative) yield coefficient value of 1 to a *reference spike-in*, arbitrarily chosen from among those that contribute a sizable fraction of total spike-in counts. Our nominal abundance of an RNA molecule is based on the temporary assumption that this molecule has the same yield coefficient as the reference spike-in. If our calibration method is supplemented with additional data on the effect that a broad range of transcript physicochemical characteristics has on library preparation and sequencing, a more realistic relative yield coefficient could be assigned to each RNA molecule of interest.

A technical statement of the outstanding problem is that our inferred nominal abundances *z*_*i*,*j*_ do not disentangle true absolute molecular abundance, *n*_*i*,*j*_, and the corresponding relative yield coefficient, *α*_*i*_; because, by definition, *z*_*i*,*j*_ = *α*_*i*_ *n*_*i*,*j*_. However, once one measures absolute cellular abundance of transcript *i* in a preparation of cells from which library *j* was derived (*n*_*i*,*j*_), the relative yield coefficient becomes known, at, least in the idealized situation ignoring various sorts of noise, because *α*_*i*_ = *z*_*i*,*j*_/*n*_*i*,*j*_. For example, *n*_*i*,*j*_ might be measured by single-cell Fluorescence *In Situ* Hybridization (FISH) methods, performed on a large population of cells from which library *j* was derived.

Statistical methods taking into account biological noise and technical noise could be used to compute a confidence interval for *α*_*i*_, provided *n*_*i*,*j*_ could be estimated. Likelihood methods could be used to integrate data across several libraries in the estimation of *α*_*i*_. In principle, once *α*_*i*_ is estimated from one or more libraries and a population of cells from which those libraries were derived, this estimate could be used for other libraries (prepared using the same protocol), past, present, and future, to allow the determination of absolute cellular molecular abundances of transcript *i*.

Modeling, like that presented in S6 Appendix and S2 Fig, and like that of [17] could also play a vital role in estimating relative yield coefficients, especially if a wider array of synthetic spike-ins covering a large gamut of physical properties were designed and utilized. Our methods have the potential of facilitating statistical modeling of RNA counts because of the explicit relationship between our nominal abundances and absolute molecular, cellular abundances of RNA. In principle, variation in counts as a consequence of true biological variation in random attomoles, *N*, and variation in counts due to variation in relative yield coefficient across transcripts with nearly identical mean abundances, *μ*_*N*_, could be disentangled.

Our approach lays the groundwork for investigating, testing, and modeling how the physical properties—e.g., length, GC content, folding energy—determine the relative yield coefficient of spike-ins and native RNA transcripts alike. Empirical measurements of relative yield coefficients, as we have defined them, and biophysical modeling could facilitate progress in making the connection between sequencing counts and the underling molecular cellular abundances of the corresponding transcripts.

### Relationship to previous studies

Our work follows up on and extends the work of [15, 16, 36, 43, 44]. Our inference method is linear and global for each library, like that of [19], [36] and [45]. We showed that our global (library specific) *ν*_*j*_ calibration constants are closely related to the Anders and Huber-like “technical” size factors of [36], which are based on spike-in counts. We called their normalization constants $s_{j}^{\text{SI}}$, and we showed that they are proportional to our *ν*_*j*_ normalization constants in the cases of 2 of our data sets with large library sizes, as predicted by theory (S8 Appendix). An important difference is that the $s_{j}^{\text{SI}}$ calibration constants are on a dimensionless scale, on the order of 1, and do not allow one to infer absolute abundances of transcripts once their relative yield coefficients become known.

[16] applied loess normalization to ERCC spike-in counts to derive a normalization function that they then applied to the counts corresponding to native RNA. Our analysis and rigorous testing of our theory and methods suggest that a local nonlinear transformation, such as loess normalization of the count data is not needed for our RNA-seq data. It seems likely that any local nonlinear fitting of counts to make replicate spike-in libraries as similar as possible would involve overfitting the data.

Our work has some important features in common with the HTN method of [46], particularly, the assumptions underlying their Eq (1) and our Eqs S1 Appendix (2) and (3). These equations explicitly allow for differences in total RNA abundance across conditions. In addition, both normalization methods are global and linear. However the HTN method of [46]: relies on having *de facto* housekeeping genes rather than experimentally-added spike-ins; does not include a model for biological noise; assumes that relative yield is simply proportional to transcript length; is focused primarily on testing for differential gene expression; and does not provide estimates of absolute RNA abundance. Their global scale factor for a given library is determined by minimizing the sum over spike-ins of the square differences between the spike-in counts in that library and those of a library chosen to be the reference library. That scale factor is then used for the native RNA counts within the same non-reference library. It can be shown that this library-by-library normalization procedure, in the limit as library size (native RNA and spike-ins) approaches infinity, will give an abundance measure that is proportional to our *z* abundance values based on *ν*_*j*_ normalization.

A quite different suite of normalization methods, called RUV (removal of unwanted variation), was introduced by [15, 36, 43, 44] and applied with great effect to many different data sets. The methods involve singular value decomposition (SVD) variant of factor analysis to compute a factor matrix *W*, which is used to model nuisance sources of variation that are unrelated to the experimental design. The factor matrix *W* is included, in addition to a design matrix, in a generalized linear model for normalized counts. One qualitative way of thinking about the *W* matrix is that is adds columns to the original design matrix for explanatory variables that one didn’t originally know about. Although this method is widely effective at reducing unwanted variation in RNA-seq data, it does not allow one to infer absolute cellular molecular RNA abundances, even if the factor matrix is computed based on spike-ins or an invariant gene set (S8 Appendix), as the authors are well aware. The simple reason is that proportion of spike-in count is tightly correlated with the biological phenomenon of interest the change of total RNA abundance with condition. However, we showed that results of our maximum likelihood normalization method can be improved, with respect to clustering and detection of differential gene expression, by applying an an RUV method based on residual, RUVr (RUVSeq package [15]) after *ν*_*j*_ normalization. We obtained closely similar results by a simpler method involving a correction factor *δ*_*j*_ for each library that was based on our discovery in a dilution study with technical replicates that we seem to have some noise in the actual overall amount of spike-ins added to the cellular RNA. We tentatively ascribed these to dilution/volume errors in handling the stock spike-in mixture. This finding highlights the importance of replacing pipetting methods for handling the spike-ins with more accurate robotic methods.

### Conclusion

The continuing discovery of examples in which there are gross transcriptome differences between cellular states, has established a need for spike-in controls in RNA-seq experiments [19]. Despite some criticisms [15], external RNA spike-ins have been adopted in several recent studies alongside methods developed to use them for RNA-seq quantitation [16, 19, 36, 46, 47].

The model presented in this work lends itself for both absolute and relative RNA quantitation, dependent on the experimental ability to accurately isolate a fixed number cells for library preparation. In both cases, we offer evidence that our approach provides reproducible results in a wide variety of conditions and has a strong predictive power. In conclusion, the presented model allows for improved unbiased RNA-seq quantitation in any experimental setup using external RNA spike-ins.

## Supporting information

S1 Appendix Maximum likelihood estimation of parameters and statistical modeling. (PDF)

S2 Appendix Simple method for removal of unwanted variation. (PDF)

S3 Appendix K-fold cross-validation analysis. (PDF)

S4 Appendix Hypothesis testing methods. (PDF)

S5 Appendix Spike-in noise. (PDF)

S6 Appendix Modeling of relative yield coefficients, *α*_*i*_. (PDF)

S7 Appendix Determination of shape parameter *a* in the yeast growth rate study. (PDF)

S8 Appendix Comparison with other normalization methods. (PDF)

S1 FigRLE plots demonstrating unwanted variation.(A) RNA abundance, as estimated by the *ν*_*j*_ maximum-likelihood normalization method, in pilot studies with 6 technical replicate RNA libraries and 2 different volumes of the stock spike-in mixture added to the RNA, 3 with high (H) volume and 3 with low (L) volume. Actual RNA abundance does not vary across replicates. Variation in the medians of the distributions of relative log expression probably reflect technical volume/dilution errors in adding spike-ins to the cellular RNA and possible within-condition errors in total RNA due to cell count or RNA extraction. Before computing relative log expression, the table of counts was filtered to include only rows corresponding to those transcripts that were detected in more than 4 of 6 libraries (3 replicates for each of 2 spike-in volume aliquots). Next, the value 1 was added to each count (${\tilde{y}}_{i,j}=y_{i,j}+1$) to ensure that the log of each corresponding abundance exists. Log transformation normalized counts ${\tilde{z}}_{i,j}={\tilde{y}}_{i,j}/\nu_{j}$ (nominal abundance) followed. (B) Relative log expression for data in panel A, but after adjustment of abundances by a single scale factor for each library to correct for putative library preparation errors. Corrected abundances were computed by ${\tilde{z}}_{i,j}^{c}\;=\;{\tilde{z}}_{i,j}/\delta_{j}$, which is equivalent to ${\tilde{z}}_{i,j}^{c}\;=\;{\tilde{y}}_{i,j}/\left(\nu_{j}\delta_{j}\right)$. See text in S2 Appendix for discussion and S2 Appendix Eq (3) used to compute *δ*_*j*_ scale factors. The vector of *δ*-values is (1.13 1.14 1.05 0.978 1.01 0.744). (C) RNA abundance (*z*-values, computed by the *ν*_*j*_ maximum likelihood method) measured at 3 different growth rates per cell in yeast growth-rate/quiescence study before correcting for library preparation errors. Median values were computed across all libraries. Within each condition there is variation in location of the 0.5 quantile of RLE. The between condition variation reflects different overall RNA abundances at the growth rates per cell of 0.12, 0.20, and 0.30 h^-1^. (D) RLE plots for yeast GR data in panel C, but after adjustment of abundances by the *δ*_*j*_ scale factor for each library. Because expected abundances do not vary within a given condition, the *δ*-values were computed separately for each growth rate per cell. The vector of *δ*-values is (0.878, 0.958, 1.19, 1.123, 0.963, 0.922, 1.01, 1.24, 0.800) in order from left to right for the corresponding libraries (x-axis labels) in panel D. These *δ*-values are commensurate with those in the dilution studies in panels A and B. (E) RLE plots based on data from the *Ciona* embryonic differentiation study. RNA abundance measured for 3 different cell types, M-Ras^CA^, Fgfr^DN^ and LacZ, before correcting for putative library preparation errors. Filtering of the table of counts was performed as in the yeast data in panels C and D. (F) RLE plots for *Ciona* data in panel E, but after adjustment of abundances by the *δ*_*j*_ correction factor for each library. The vector of *δ*-values is (1.07, 1.17, 0.798, 1.48, 0.706, 0.959, 1.34, 1.32, 0.566) in order from left to right for the corresponding libraries (x-axis labels) in panel D.(TIF)

S2 FigSpike-in noise.(A) Comparison of observed and predicted counts, in MA-like plot format, according to the multinomial statistical model linking spike-in counts to abundance in the corresponding sample. Each plotted point represents log_2_ ratio of observed to predicted spike-in counts for each detected molecule *i* in one or more replicates *j* vs. log_2_ of the predicted counts. Data from the *Ciona* embryonic differentiation study. The mean log_2_ ratio of observed to predicted spike-in counts is equal to 0.046, which is consistent with unbiased prediction. Vertical bars with lower and upper endpoints (*L*, *U*) demarcate a mid 0.99 quantile range of random counts generated from the multinomial model with the maximum likelihood proportions, such that Pr{*Y* < *L*} < 0.005, and Pr{*Y* > *U*} < 0.005. Because the marginal probability mass functions are binomial, the (*L*, *U*) interval for each transcript is the same as the mid 0.99 binomial quantile for that transcript. (B) Comparison of observed and predicted counts from multinomial spike-in model, as in panel A, but based on data from the yeast dilution study. (C) Comparison of observed and predicted counts from multinomial spike-in model, as in panel A, but based on data from the yeast growth rate study. (D) CV^2^(mean) versus mean for normalized spike-in counts, on log-log axes, for the same *Ciona* spike-in data (open symbols) in panel A. For each spike-in *i* the mean normalized count plotted on the *x*-axis is the mean over all libraries *j* of $y_{i,j}/L_{j}^{\text{SI}}$. The corresponding squared CV is plotted on the y-axis. The solid black line connects the theoretical population CV^2^ values according to the multinomial model, and it is drawn from S5 Appendix Eq (3). The solid red line is the theoretical population CV^2^ that follows from a negative binomial model for random spike-in counts *Y*_*i*,*j*_, in which the mean is given by S5 Appendix Eq (1) and the shape parameter is a single value, *a* = 1000. It is drawn from S5 Appendix Eq (4). The dotted red lines demarcate the mid 0.99 quantile range of CV^2^ values generated in 10,000 Monte Carlo simulations in which the synthetic spike-in counts were drawn form the negative binomial (*a* = 1000). (E) CV^2^(mean) versus mean for normalized spike-in counts, as in panel B, but based on spike-in counts in the yeast dilution study. Solid black line from the multinomial model (S5 Appendix Eq (3). Solid red line is from negative binomial model with *a* = 1000 (S5 Appendix Eq (4). (F) CV^2^(mean) versus mean for normalized spike-in counts, as in panel B, but based on spike-in counts in the yeast growth rate study. Solid black line from the multinomial model (S5 Appendix Eq (3). Solid red line is from negative binomial model with *a* = 400 (S5 Appendix Eq (4)).(TIF)

S3 Fig Relative yield coefficients and their dependence of length, GC content, and folding energy. (A) Histogram of relative yield coefficients (*α*_*i*_) based on spike-in counts from 9 libraries in the *Ciona* embryonic differentiation study. The median *α*-value is 0.95, and the interquartile range (IQ) of 1.3 extends from 0.51 to 1.8. (B) Observed (and fitted values of *α*_*i*_, in the *Ciona* embryonic differentiation study, based on a mathematical model (S6 Appendix Eq (1)) including spike-in length (nt), GC content, and folding energy (Kcal/mol). The vertical bars delineate the mid 0.95 quantile of *α*-values, computed according to the multinomial model when the empirical proportions for the spike-ins are used as stand-ins for the true population proportions. In some cases, the filled symbol obscures exceptionally narrow mid 0.95 quantile ranges of the *α*_*i*_ values. The maximum likelihood values of the *β*-coefficients (see text) in S6 Appendix Eq (1) are: *β*_1_ = 0.013; *β*_2_ = 0.0032; and *β*_3_ = −0.0014. The root mean square error, normalized by max(*α*) − min(*α*), or normalized by sd(*α*) are equal to 0.16 or 0.98, respectively. (C) Histogram of relative yield coefficients (*α*_*i*_) based on spike-in counts from 6 libraries in the yeast dilution study (different library preparation protocol than that used in the *Ciona* embryonic differentiation study). The median of *α*-value is 0.65, and the IQ range of 0.83 extends from 0.28 to 1.1. (D) Observed (values in panel C) and fitted values of *α*, for yeast dilution study, based on S6 Appendix Eq (1). The maximum likelihood values of the *β*-coefficients S6 Appendix Eq (1) are: *β*_1_ = 0.0012; *β*_2_ = 0.0045; and *β*_3_ = 0.140. All 3 variables were found to be highly significant (see text). The root mean square error, normalized by max(*α*) − min(*α*), or normalized by sd(*α*) are equal to 0.14 or 0.93, respectively.
(TIF)

S4 Fig Comparison of distributions of CV(mean) for laboratory, and synthetic data. A rigorous challenge of our statistical model, with a single shape parameter *a* for each condition in the yeast GR study, is provided by looking at the overall distributions of CV(mean), and the distributions of CV(mean) for transcripts whose means fall into each of several subintervals of the range of mean values. (A) Histograms of sample estimates of CV(mean), plotted on a density scale, corresponding to the yeast quiescence study with C-limited growth at rate per cell of 0.12 h^-1^. Solid grey line correspond to experimental data (CV values computed from 3 replicates), and the dashed black lines are for corresponding synthetic (Monte Carlo) data generated according to the negative binomial model in S7 Appendix Eq (1). Mean ± sd of the distributions for lab, and synthetic data are 0.19±0.19 and 0.19±0.19, respectively. Shape parameter for synthetic data, *a* = 23, determined by a maximal marginal likelihood method (S6 Appendix). (B-D) CV(mean) for sample mean values in the the top 3 of 4 bins of equal width on the logarithmic scale of mean values. The spike at a CV equal to 1 corresponds to transcripts that were detected in only 1 out of 3 replicates, and it stems from those transcripts expressed at very low copy number (nominal abundance of ∼ 2 transcripts per 1000 cells). The spikes in CV values between roughly 0.5 and 1 (panels A–C) come exclusively from transcripts with very low levels of expression. (E–H) Similar to (A–E), but data are from libraries corresponding to growth rate per cell of 0.20 h^-1^. Mean ± sd of the full distributions for lab, and synthetic data in panel (E) are 0.19±0.22 and 0.19±0.21, respectively. Shape parameter for synthetic data, *a* = 34. (I–L) Similar to (A–E) but data are from libraries corresponding to growth rate per cell of 0.30 h^-1^. Mean ± sd of the full distributions for lab, and synthetic data in panel (E) are 0.21±0.23 and 0.20±0.21, respectively. Shape parameter for synthetic data, *a* = 24.
(TIF)

S5 Fig RUV normalization following maximum-likelihood *ν*_*j*_ normalization. (A) Diagnostic relative log expression (RLE) plots of adjusted counts obtained by applying an RUVr normalization method based on residuals to *ν*_*j*_-normalized counts (*z* abundance values) from yeast GR study (R function RUVr in the RUVSeq package [15]). Before application of RUVr, the table of counts was filtered to include only rows for those transcripts that were detected in more than 6 of 9 libraries (3 replicates for each of 3 growth rates per cell). This eliminates transcripts that were not detected at all in one or more of the 3 conditions. Next, the value 1 was added to each count, as usual, to ensure that the log of each count exists. Next, *ν*_*j*_ normalization was applied, followed by RUV normalization. These RLE plots resemble those for the *ν*_*j*_-normalized data, followed by *δ*_*j*_ to correct for putative volume/dilution/extraction/counting errors, in S1D Fig, in the variation within condition, and the variation of the 0.5 quantile of the log relative expression distributions across conditions. In principle, the two correction methods, the RUVr method with the factor matrix *W* and the *δ* correction factor method could yield identical results in the special case with one factor of variation where **W** = ± log ***δ*** (S8 Appendix). We did indeed find these 2 sets of constants to be similar for our yeast data, with correlation coefficient equal to 0.83. (B) Diagnostic principal components analysis (PCA) biplot of the normalized scores of the second, versus the first principal component, corresponding the normalized counts matrix for panel for panel A. The seemingly good clustering of the libraries in this plot is confirmed by kmeans, and hierarchical clustering. Each of the 3 clusters contains libraries from only one condition. (C) Similar to panel A, but based on data from the *Ciona* embryonic differentiation study. These RLE plots are similar to those in S1F Fig, where the original counts were *ν*_*j*_ normalization was followed by *δ*_*j*_ normalization to correct for putative library preparation errors. We found the elements of **W** and log ***δ*** to be quite similar for the *Ciona* data: ***W*** = [0.016, -0.29, 0.21, -0.40, 0.35, 0.043, -0.28, -0.30, 0.65], and −log ***δ*** = [-0.071, -0.15, 0.22, -0.40, 0.35, 0.042, -0.29, -0.28, 0.57], with correlation coefficient equal to 0.98. (D) PCA biplot corresponding to panel C.
(TIF)

S1 Code and Data Demonstration R Markdown file, associated R functions, and all data files. The YmatRNAyeast.txt, YmatRNAciona.txt, and YmatRNAdilution.txt files are data frames for native RNA count data (genes-by-replicates) for the yeast growth rate study, *Ciona* embryonic differentiation study, and yeast dilution study, respectively. The corresponding count data for spike-in molecules are named similarly, but with SI instead of RNA. Data files ERCCyeast.txt and ERCCciona.txt are data frames for detected ERCC spike-in molecules in the yeast growth rate study and *Ciona* study, giving attomoles of each molecule added to native RNA, length (nt), GC content, and folding energy (Kcal mol^-1^). The ERCCdilutionNmatAttomoles.txt file is a data frame for the yeast dilution study that gives attomoles of detected spike-in molecules for the high-volume (H) and low-volume (L) spike-in aliquot conditions.
(ZIP)
