# Supplementary material for: A complete statistical model for calibration of RNA-seq counts using external spike-ins and maximum likelihood theory
Source: PLoS Comput Biol. 2019 Mar 11;15(3):e1006794. doi: 10.1371/journal.pcbi.1006794 (PMC6428340; doi:10.1371/journal.pcbi.1006794)
Supplement: S1 Appendix — (PDF) [file pcbi.1006794.s001.pdf]

Maximum likelihood estimation of parameters and statistical modeling

Estimation of RNA abundances and spike-in relative yield coefficients

For sake of clarity we say at the outset that our major focus is on estimation of RNA abundances. The estimation of spike-in relative yield coefficients is for the sake of exploring biophysical modeling (below), with possible future application to aid in the estimation of relative yield coefficients of native RNA. Also, to aid the reader, we point out that our maximum likelihood method for estimating abundances requires pooling data across transcripts, spike-ins and libraries to compute an overall or total log-likelihood. The reason that all libraries are coupled is because the abundances of spike-ins are identical across libraries.

With the multinomial assumption for sampling noise, for given abundances of the s spike-in molecules (z_i, for $i = 1, 2, \ldots, s$) and for the q detected RNA molecules ($z_{i,j}$, for $i = s+1, s+2, \ldots, s+q$), and the total library size $\mathcal{L}_j^{\text{tot}}$, the joint distribution of counts (sequencing reads) for molecules i in library j — i.e., the joint probability mass function (same as the likelihood function) for random counts $\{Y_{1,j}, Y_{2,j}, \ldots, Y_{s+q,j}\}$ — is given by

$$\Pr\{\,(Y_{1,j} = y_{1,j}) \cap \cdots \cap (Y_{s+q,j} = y_{s+q,j})\,|\,\mathcal{L}_j^{\text{tot}}\} \;= \mathcal{L}_j^{\text{tot}}! \prod_{i=1}^{s+q} \frac{(p_{i,j})^{y_{i,j}}}{(y_{i,j})!}. \tag{1}$$

In Eq (1) we distinguish random variables, written in upper case, from particular observed values, written in lower case. The count proportions for molecule i in library j, $p_{i,j}$, are somewhat different for spike-ins and RNA molecules, because the spike-in abundances are the same for each library. For spike-ins the expected proportions (for given z-values) are given by

$$p_{i,j} \;=\; \frac{z_i}{\sum\limits_{i=1}^{s} z_i \;+\; \sum\limits_{i=s+1}^{s+q} z_{i,j}}, \;\text{ for } i = 1, 2, \ldots, s,, \tag{2}$$

and, for RNA molecules, the expected proportions are given by

$$p_{i,j} \;=\; \frac{z_{i,j}}{\sum\limits_{i=1}^{s} z_i \;+\; \sum\limits_{i=s+1}^{s+q} z_{i,j}}, \;\text{ for } i = s+1, s+2, \ldots, s+q. \tag{3}$$

We define LL^{tot} as the component of the overall log-likelihood of the data (i.e., log of the right-hand size of Eq (1)) that depends on the parameters to be estimated. LL^{tot} is given by the sum over i and j of the log-likelihood of the individual observations, $LL_{i,j}$. The total log-likelihood is given by

$$\begin{aligned} LL^{\text{tot}} \;&= \sum_{j=1}^{r} \sum_{i=1}^{s+q} LL_{i,j} \\ &= \sum_{j=1}^{r} \sum_{i=1}^{s+q} y_{i,j}\, \log p_{i,j} \end{aligned} \tag{4}$$

To find all estimated z-values, we substitute Eqs (2) and (3) for $p_{i,j}$ in Eq (4) above, and set the gradient of LL^{tot} equal to zero. The gradient is with respect to s individual z_i variables for the spike-ins ($i = 1, 2, \ldots, s$) and $q \cdot r$ individual $z_{i,j}$ values for the

cellular RNA molecules (q identified RNA molecules and r libraries; so $j = 1, 2, \ldots, r$, and $i = s+1, s+2, \ldots, s+q$, for RNA molecules). According to convention, the maximum-likelihood estimators we find below would be denoted by $\hat{z}_i$ (or z_i^{ML}) and $\hat{z}_{i,j}$ (or $z_{i,j}^{\mathrm{ML}}$). However, for sake of streamlining notation we will use neither modifier of the z-values.

The gradient equations for the spike-ins are

$$0 = \frac{\partial}{\partial z_i} LL^{\mathrm{tot}} \; = \; \frac{1}{z_i} \sum_{j=1}^{r} y_{i,j} - \sum_{l=1}^{r} \frac{\mathcal{L}_l^{\mathrm{tot}}}{\sum\limits_{k=1}^{s} z_k \; + \sum\limits_{k=s+1}^{s+q} z_{k,l}}, \text{ for } i = 1, 2, \ldots, s. \tag{5}$$

Using Eq(5) to solve for z_i/z_1, and using $z_1 = n_1$ by definition, gives

$$z_i \; = \; n_1 \frac{\sum\limits_{j=1}^{r} y_{i,j}}{\sum\limits_{j=1}^{r} y_{1,j}} \qquad \text{for } i = 1, \ldots s. \tag{6}$$

Eq(6) can also be written as

$$z_i \; = \; \frac{n_1}{f_1} f_i \qquad \text{for } i = 1, \ldots s, \tag{7}$$

where f_i is the empirical fraction of total spike-in counts, across all libraries that is accounted for by spike-in molecule i; i.e.,

$$f_i \stackrel{\mathrm{def}}{=} \frac{\sum\limits_{j=1}^{r} y_{i,j}}{\sum\limits_{k=1}^{r} \mathcal{L}_k^{\mathrm{SI}}}, \tag{8}$$

and

$$\mathcal{L}_k^{\mathrm{SI}} = \sum_{i=1}^{s} y_{i,k} \tag{9}$$

is the size of spike-in library k. The reader might find it helpful to keep in mind that the subscript 1 in Eq (7) reflects that fact that we have assigned the index 1 to the reference spike-in, and also reflects that fact that the relative yield coefficient of the reference spike-in is equal to 1, by definition. In the case of spike-in molecule, we can estimate the relative yields, α_i, from the z_i by

$$\begin{aligned} \alpha_i \; &= \frac{z_i}{n_i} \\ &= \frac{n_1}{f_1} \frac{f_i}{n_i}. \end{aligned} \tag{10}$$

For native RNA molecules, the gradient equations are

$$0 = \frac{\partial}{\partial z_{i,j}} LL^{\mathrm{tot}} \; = \; \frac{y_{i,j}}{z_{i,j}} - \frac{\mathcal{L}_j^{\mathrm{tot}}}{\sum\limits_{k=1}^{s} z_k \; + \sum\limits_{k=s+1}^{s+q} z_{k,j}}, \tag{11}$$

or

$$z_{i,j} \; = \; y_{i,j} \left(\frac{\sum\limits_{k=1}^{s} z_k \; + \sum\limits_{k=s+1}^{s+q} z_{k,j}}{\mathcal{L}_j^{\mathrm{tot}}} \right). \tag{12}$$

Computing the two sum terms in numerator of Eq (12) will allow us to solve for the abundance $z_{i,j}$ in this equation. From Eq (7),

$$\sum_{k=1}^{s} z_k = \frac{n_1}{f_1}. \tag{13}$$

To compute the second sum term in the numerator of Eq (12), we substitute Eq (13) into Eq (12), and sum both sides from $i = s$ to $i = s + q$, to obtain

$$\sum_{k=s+1}^{s+q} z_{k,j} = \frac{n_1}{f_1} \frac{\mathcal{L}_j^{\mathrm{RNA}}}{\mathcal{L}_j^{\mathrm{SI}}}. \tag{14}$$

Finally, substituting Eqs (13) and (14) into Eq (12) gives maximum likelihood abundances for RNA molecules.

$$z_{i,j} = \frac{y_{i,j}}{\nu_j} \quad \text{for } i = s+1,\ s+2, \ldots, s+q, \text{ and } j = 1, 2, \ldots, r \tag{15}$$

where

$$\nu_j \stackrel{\text{def}}{=} \frac{f_1 \mathcal{L}_j^{\mathrm{SI}}}{n_1}. \tag{16}$$

From abundance to counts

For each library, we take a population perspective and focus on all such libraries in which the total library size is equal to the observed size, $\mathcal{L}_j^{\mathrm{tot}}$, and the spike-in library size is equal to the observed size $\mathcal{L}_J^{\mathrm{SI}}$. Note that these library sizes imply that the total RNA library size is $\mathcal{L}_j^{\mathrm{RNA}} = \mathcal{L}_j^{\mathrm{tot}} - \mathcal{L}_j^{SI}$. If we condition the joint distribution for sequencing reads given in Eq (1), with size parameter $\mathcal{L}_j^{\mathrm{tot}}$, on the total spike-in library size $\mathcal{L}_j^{\mathrm{SI}}$, the marginal distribution of RNA counts within the RNA library is multinomial with size parameter $\mathcal{L}_j^{\mathrm{RNA}}$. In a population (of experiments) statistical model, the RNA abundances are random variables (denoted by upper case letters), $Z_{i,j}$ and, consequently, the multinomial proportions are random variables, $P_{i,j}$, where

$$P_{i,j} = \frac{Z_{i,j}}{\sum_{k=s+1}^{s+q} Z_{k,j}}. \tag{17}$$

Because the large multinomial size parameter $\mathcal{L}_j^{\mathrm{RNA}}$ and small proportions $P_{i,j}$, the multinomial distribution for RNA counts is well approximated as a product of independent Poisson probability mass functions, in which each Poisson mean parameter $\Lambda_{i,j}$ is equal to the corresponding mean in the multinomial distribution; i.e.,

$$\begin{aligned} \Lambda_{i,j} &= P_{i,j} \mathcal{L}_j^{\mathrm{RNA}} \\ &= \frac{Z_{i,j}}{\sum_{k=s+1}^{s+q} Z_{k,j}} \mathcal{L}_j^{\mathrm{RNA}}. \end{aligned} \tag{18}$$

The fact that each Poisson mean for count Y of each RNA molecules depends nonlinearly on all RNA abundances, $Z_{i,j}$, complicates statistical modeling. However, in the context of our maximum likelihood approach, it seems reasonable to replace the denominator in Eq (18) with our empirical maximum likelihood estimate, given by Eq (14).

If we do so, the random Poisson means become independent:

$$\Lambda_{i,j} = \nu_j \, Z_{i,j}. \tag{19}$$

where ν_j is as defined in Eq (16). Note that ν_j is a single, library-specific scale factor. To be explicit, the probability mass function for the random counts corresponding to a given RNA transcript abundance, $Z_{i,j}$, is given by

$$\Pr\{Y_{i,j} = y_{i,j} \,|Z_{i,j}\} = \frac{(\nu_j Z_{i,j})^{y_{i,j}}}{y_{i,j}!} \exp(-\nu_j Z_{i,j}). \tag{20}$$

To complete our statistical model we assume that each RNA abundance Z (suppressing subscripts for the sake of notational simplicity) is a gamma-distributed random variable [34] with mean μ_Z and shape parameter a. As a consequence, the unconditioned probability mass function for the corresponding counts Y is negative binomial [34] with mean $\mu_Y = \nu\mu_Z$ and shape parameter a, where ν is library-specific, and both μ_Z and a are transcript- and condition-specific, in general.
