# Supplementary material for: A complete statistical model for calibration of RNA-seq counts using external spike-ins and maximum likelihood theory
Source: PLoS Comput Biol. 2019 Mar 11;15(3):e1006794. doi: 10.1371/journal.pcbi.1006794 (PMC6428340; doi:10.1371/journal.pcbi.1006794)
Supplement: S2 Appendix — (PDF) [file pcbi.1006794.s002.pdf]

Simple method for removal of unwanted variation

Dilution study RLE plots reveal unwanted variation

S1 Fig A shows RLE plots [35] of estimated RNA abundances (z-values) for each of 3 replicates prepared with each of 2 spike-in aliquot volumes, high and low. Because the 6 RNA samples are technical replicates, we expect the estimated RNA abundance by our normalization method to be virtually identical across libraries. The variation in location of the median of the distribution of relative log expression values across conditions is most likely due to technical error in measuring out the spike-ins added to the RNA samples and/or slight variations in RNA extraction within conditions. The upshot is that the attomoles of the reference spike-in, n_{ref}, in the equation for the library-specific normalization constant ν_j, which we assumed is constant within each condition, could vary randomly to some extent, and the total cellular RNA could vary a bit within a condition. We will refer to such errors a library preparation errors.

We estimated the scaling-factor to correct for putative errors by normalizing the abundance z-values (nominal attomoles) in two different ways and obtained virtually identical results. For the sake of brevity, we ignore here filtering of the raw counts matrix and the addition of 1 to ensure that the log of each abundance exists. One method is qualitatively similar to the RUVr factor analysis method [15, 43, 44] based on residuals with 1 source of unwanted variation (see S8 Appendix). In this method, log abundance values, for libraries j in the set of libraries corresponding to condition l, Ω_l, are regarded as being shifted shifted slightly by single, library specific, nuisance factors β_j; and we want to correct for these. To do so, log abundances within a condition l are fitted (minimum sum of square deviations) by

$$\begin{aligned} \log z_{i,j} &= \log \mu_i^l + \beta_j, \text{ with the constraint that} \\ \sum_j \beta_j &= 0, \end{aligned} \tag{1}$$

where μ_i^l is the average abundance value for transcript i over libraries in condition l. This is equivalent to library-specific multiplicative corrections by factors δ_j, where

$$\begin{aligned} z_{i,j} &= \delta_j\, \mu_i^l, \text{ where} \\ \delta_j &= \exp(\beta_j), \text{ with the constraint that} \\ \sum_j \log \delta_j &= 0, \text{ or} \\ \prod_j \delta_j &= 1. \end{aligned} \tag{2}$$

For the dilution study, in which the true RNA abundance is supposed to be identical across all 6 libraries for each transcript, the subscript l in Ω_l takes on only one value, $l = 1$, and $\Omega_1 = \{1, 2, 3, 4, 5, 6\}$.

The general solution to Eq (1), for library with j in condition l ($j \in \Omega_l$) is

$$\begin{aligned} \beta_j &= \operatorname*{mean}_i (\log z_{i,j}) - \operatorname*{mean}_{i,k\in\Omega_l}(\log z_{i,k}), \text{ and} \\ \delta_j &= \exp(\delta_j) \end{aligned} \tag{3}$$

and the corresponding library-specific scaling factor on the counts is $\delta_j = \exp(\beta_j)$. In our other correction method, an Anders and Huber-like scaling [34]factor for a given replicate is computed as the median of the ratio, for each transcript, of its z-value over

its geometric mean across all n_r lanes in the same condition:

$$\delta_j \; = \; \underset{i}{\text{median}} \frac{z_{i,j}}{\left(\prod_{k \in \Omega_l} z_{i,k} \right)^{1/n_r}} \text{ for } j \in \Omega_l. \tag{4}$$

This δ_j correction factor is similar to the ξ_j factor of [36] (see S8 Appendix, Eq (4)), given by the quotient of biological and technical "size factors," s_j^B / s_j in their notation, where s_j^B is the median-based library size factor based on counts from biological RNA [34], and s_j^{SI} is the median-based library size factor based on spike-in counts only. We emphasize that our δ_j is based on counts from libraries within the same condition ($j \in \Omega_l$).

The two different methods above for computing δ-values gave virtually identical values. We found the vector of δ-values in the dilution study to be (1.13,1.14, 1.05, 0.978, 1.01, 0.744). RLE plots for the corrected abundances are shown in S1 Fig B.

Yeast GR study RLE plots have similar unwanted variation

Fig S1 Fig C shows RLE plots for RNA abundance estimated by the maximum likelihood (ν_j) method from 3 replicate libraries at each of 3 growth rates, $0.12\,\mathrm{h}^{-1}$ (red), $0.20\,\mathrm{h}^{-1}$ (green), and $0.30\,\mathrm{h}^{-1}$ (blue). The plot in panel C shows clear differences of the median relative log expression between conditions, with low, medium and high relative expression for growth rates of 0.12, 0.20, and $0.30\,\mathrm{h}^{-1}$. However there is unwanted variation within each group as well. The results for the dilution study above suggest that the within group variation could come from library preparation errors. Because we allow for overall abundance of RNA to differ between growth rates, we normalized expression within each group in the same way that we normalized across all libraries in the technical replicates in the dilution study above, in which there is only 1 condition by definition.

It is important to note that, because the geometric mean of δ-values within a condition is always equal to 1 (mean log equals zero), the δ correction factors do not change the geometric mean value (mean log value) of any transcript within any condition. Consequently, the δ correction factors do not introduce artifactual fold differences between conditions. Our statistical way of thinking about this issue is that, for a given library and condition, either the spike-in abundances and/or the expected RNA abundances in the corresponding sample to be sequenced are globally scaled (multiplicatively) by a common random variable. In the condition-wise computation of the correction factors, we are exploiting the fact that the true population value of expected RNA abundance is common among replicates. We show in a section below, entitled "RUV normalization followed by regression," that one in a suite of RUV methods [15, 43, 44], RUVr, produces very similar scalings (corrections) when it is applied to our ν_j normalized counts, and we explain the underlying theoretical reason.

We found the vector of δ-values (0.802, 0.930, 1.34, 1.21, 0.933, 0.886 1.03 1.35 0.724, from left to right) to be commensurate with the technical errors we identified in the dilution study above. RLE plots for the corrected abundances are shown in Fig S1 Fig D. The tighter spread of relative log expression within replicates for the growth rate of $0.20\,\mathrm{h}^{-1}$ is consistent with the larger value of the shape parameter a that we found for this condition (see below). We found similar reduction in unwanted variation by applying the RUVr method [41] to the ν_j-normalized abundance (z) values, as shown in S5 Fig and discussed in S8 Appendix.

Ciona embryonic differentiation study RLE plots show similar variation

Cellular differentiation during embryonic development is characterized by dramatic changes in expression. S1 Fig E and F are RLE plots like those in panels C and D, but they are based on data from the *Ciona* embryonic differentiation study. The plots are based on data from 3 replicates for each of 3 cell types, M-Ras^{CA} (red), Fgfr^{DN} (green), and LacZ (blue). As in the yeast data, RLE plots of abundances according to the ν_j normalization method in panel E show variation within condition of the median of the distributions of log relative error. When we assumed that all this within-condition variation stems from spike-in volume/dilution/extraction/counting errors and performed and corrected abundances within each group as above, we found the vector of δ values (correction factors) to be (1.07, 1.17, 0.798, 1.48, 0.706, 0.959, 1.34, 1.32, 0.566). These δ values are similar to those obtained for the yeast data above. RLE plots for corrected abundance values are shown in panel F. Again, we found similar reduction in unwanted variation by applying the RUVr method [41] to the ν_j-normalized abundance (z) values (see S5 Fig and S8 Appendix).
