# Supplementary material for: A complete statistical model for calibration of RNA-seq counts using external spike-ins and maximum likelihood theory
Source: PLoS Comput Biol. 2019 Mar 11;15(3):e1006794. doi: 10.1371/journal.pcbi.1006794 (PMC6428340; doi:10.1371/journal.pcbi.1006794)
Supplement: S3 Appendix — (PDF) [file pcbi.1006794.s003.pdf]

K-fold cross-validation analysis

In the k-fold cross-validation procedure, we test our abundance estimation method for its ability to recover absolute spike-in abundances in a leave out condition. In this method, we compute the spike-in abundance estimate $z_{i,j}$, using Eqs (1) and (2) in the paper (in which f_1 is computed from the complement of the leave-out libraries), for each library j in the leave-out set , and then divide by α_i as defined S1 Appendix Eq (10). In this battery of tests, we choose to use as a stand-in for the true population value of α_i the estimator based on all libraries. The philosophy here is that as the number of libraries for which the method has been applied grows over time with applications the estimated α-values become equivalent, for all practical purposes, with the true α-values. Moreover in any practical setting in which a yield coefficient is used to estimate $n_{i,j}$ from $z_{i,j} = \alpha_i n_{i,j}$ the yield coefficient would be assumed to be a fixed constant rather than variable over libraries. According to this procedure, we can write out explicitly what we get for the reconstructed attomoles of spike-in molecule i in replicate j in a leave-out condition:

$$\begin{aligned}\hat{n}_{i,j} &= \frac{\hat{z}_{i,j}}{\hat{\alpha}_i} \\ &= n_i \left(\frac{f_{i,j}}{f_i}\right)\left(\frac{f_1}{f_1^{\mathrm{IN}}}\right),\end{aligned} \tag{1}$$

where $f_{i,j}$ is the actual fraction of spike-in counts in library j that is accounted for by spike-in i, f_i is the corresponding global fraction as defined in S1 Appendix Eq (8), f_1 is the global proportion of the reference spike-in, and f_i^{IN} is the proportion of the reference spike-in in the leave-in set (complement of the 3 replicates in the leave-out condition). The errors in Eq (1) stem primarily from multinomial noise from spike-ins with low proportions for which departures of the ratio $\frac{f_{i,j}}{f_i}$ from 1 are most severe. But there is also unexplained noise in the smaller libraries where the $\frac{f_{i,j}}{f_i}$ ratio varies from 1 even for proportions that are not terribly small, as evidenced in S2 Fig.

We quantify error (deviation) between inferred, and actual abundance, $\hat{n}_i$ and n_i respectively, per cell by an average mean-fold error, MFE, over spike-ins, where the inferred molecules per cell is the average over the 3 replicate libraries comprising the leave-out condition, l; Our MFE gives equal weight to a particular ratio of inferred abundance to actual abundance and to its reciprocal; e.g., a ratio of 1/2 and 2 contribute equally to MFE. This metric for error is akin to root-mean-square error. Consequently, MFE ≥ 1 by definition. For a given leave-out condition l we first compute the mean absolute value of the $\log_2$ ratio,

$$\mathrm{MAE}_l = \operatorname*{mean}_{i \leq s} \left| \log_2 \left(\frac{\mathrm{mean}_{j \in \Omega_l}(\hat{n}_{i,j})}{n_i} \right) \right|, \tag{2}$$

and then MFE is given by

$$\mathrm{MFE}_l = 2^{\mathrm{MAE}_l} \tag{3}$$

To quantify performance based on MFE, we compute the ration of MFE to a MFE generated by a Monte Carlo trial ($\mathrm{MFE}_{\mathrm{syn}}$) in which the matrix of spike-n counts is generated randomly based on the multinomial model, and we did this 10,000 times. In Fig 2 the average ratio is plotted with a symbol for each leave-out condition, and the mid 0.95 quantile is delineated by a line through the average value.
