# Supplementary material for: A complete statistical model for calibration of RNA-seq counts using external spike-ins and maximum likelihood theory
Source: PLoS Comput Biol. 2019 Mar 11;15(3):e1006794. doi: 10.1371/journal.pcbi.1006794 (PMC6428340; doi:10.1371/journal.pcbi.1006794)
Supplement: S4 Appendix — (PDF) [file pcbi.1006794.s004.pdf]

Hypothesis testing methods

Testing dependence of RNA abundance on growth rate in yeast

To keep the number of parameters in our statistical model to a minimum, we assume that the negative binomial shape parameter, a, is fixed across RNA transcripts within each condition (growth rate) with values a-values of 23, 34, and 24 for growth rates per cell of 0.12, 0.20, and 0.30 h^{-1}, as obtained in S7 Appendix.

For the sake of streamlining notation, we consider any particular RNA transcript, so we can suppress the subscript i. Also, we ignore the δ_j correction factors in the equations below (but not in our analysis) to simplify notation. For a given replicate j, the model for the population mean says

$$\mu_Z(\gamma_j) \;=\; \exp\left(\phi_0 + \phi_1[\gamma_j - \bar{\gamma}]\right), \tag{1}$$

in which $\exp(\phi_0)$ gives the interpolated value of μ_Z at the mean of the experimental growth rates; i.e. $\bar{\gamma} = (0.12 + 0.20 + 0.30)/3 \;=\; 0.21$, which happens to be very close to the middle growth rate. It is worth noting that the assumed linear dependence of the log of abundance on explanatory variables is a standard generalized linear model (GLM) in RNA-seq analysis [14]. The null and alternative hypotheses are:

$$\mathrm{H_0} : \; \phi_1 \; = 0 \text{ and} \tag{2}$$

$$\mathrm{H_A} : \; \phi_1 \; \neq 0, \tag{3}$$

respectively.

We tested $\mathrm{H_0}$ for each RNA transcript by a method based on a standard test statistic, -2 times the log of a ratio of maximum likelihoods ($\mathrm{H_A}$ vs. $\mathrm{H_0}$), often referred to as the LRT test. Because our test statistic, κ^2, is computed with only 9 replicates across the 3 growth rates, one cannot count on the asymptotic χ^2 distribution with 1 degree of freedom. Therefore, we computed empirical p-values for each RNA transcript from 10,000 sets of Monte Carlo (MC) trials. In each set, each of 9 synthetic counts Y_j was sampled independently from negative binomial population, according to $\mathrm{H_0}$, with the maximum likelihood value for the parameter ϕ_0 under $\mathrm{H_0}$. The sampling was done with R's rnbinom( ) function. In each MC set, the random counts $(Y_1, Y_2, ..., Y_9)$ were treated exactly as if they were experimental counts; for each, a test statistic κ^2 was computed and recorded. The empirical p-value was given by the fraction of MC κ^2-values that were greater than the original experimental test statistic κ^2. After obtaining the list of p-values for all the genes, we computed the adjusted p-values, according to the Benjamini and Hochberg method [39]. The empirical p-value approach was more conservative (4200 genes) than the LRT test (4302 genes).

Hypothesis testing for differential gene expression in *Ciona* embryonic differentiation study

To test for differential gene expression among the 3 cell types we applied the DESeq function, in the DESeq2 [14] package, to our RNA count matrix; but we used the DESeq function option of replacing the default s_j median size factors with other size factors of of one's choice. Because the DESeq function "expects" dimensionless library size factors on the order of 1, and the default size factors for our spike-in count matrix have a geometric mean close to 1, we chose size factor that are a simple universal scaling of our $\nu_j\delta_j$ normalization factors, namely size factors given by $s_j = \nu_j\delta_j / \left[\prod_{k=1}^{r} \nu_k\delta_k\right]^{1/r}$, that have a geometric mean of 1. On a cautionary note, the abundances reported by the

DESeq function, with these size factors would have to be divided by $\left[\prod_{k=1}^{r} \nu_k \delta_k\right]^{1/r}$ to recover the original nominal abundances produced by the $\nu_j \delta_j$ normalization.
