# Supplementary material for: A complete statistical model for calibration of RNA-seq counts using external spike-ins and maximum likelihood theory
Source: PLoS Comput Biol. 2019 Mar 11;15(3):e1006794. doi: 10.1371/journal.pcbi.1006794 (PMC6428340; doi:10.1371/journal.pcbi.1006794)
Supplement: S5 Appendix — (PDF) [file pcbi.1006794.s005.pdf]

Sike-in noise

An intuitive way of examining the extent to which spike-in counts adhere to the the simple multinomial model for sampling noise is by way of MA-like plots in S2 Fig A–C, in which log fold-difference between observed and expected counts is plotted versus the log of expected counts. These figures complement those in Fig 2 in the main body of this paper. In S2 Fig A we compare observed, $y_{i,j}$, to expected, $\hat{y}_{i,j}$, counts for all 9 libraries in the *Ciona* embryonic differentiation study. According to the multinomial statistical model for all counts (RNA and spike-ins), the spike-in counts within a spike-in library also follow a multinomial distribution. According to the multinomial model, spike-in counts, the expected value of random counts for spike-in i in library j, given the total spike-in library size $\mathcal{L}_j^{\mathrm{SI}}$ is given by the spike-in proportion p_i multiplied by the total spike-in library size; i.e.,

$$\mathbb{E}[Y_{i,j} \,|\, \mathcal{L}_j^{\mathrm{SI}}] \;=\; p_i \, \mathcal{L}_j^{\mathrm{SI}}, \tag{1}$$

where p_i is the population proportion of spike-in molecule i within a total spike-in library. We substitute sample estimators f_i for the true population proportions p_i to obtain the expected spike-in counts, $\hat{y}_{i,j}$, according to the multinomial model,

$$\hat{y}_{i,j} = f_i \mathcal{L}_j^{\mathrm{SI}}. \tag{2}$$

Vertical bars (panel A) with lower and upper endpoints (L, U) demarcate the mid 0.99 quantile range of random difference between the logs. Because the marginal probability mass function (pmf) for each spike-in is binomial, in the multinomial model, the (L, U) interval for each molecule is computed from the 0.005 and 0.995 quantiles of the appropriate binomial pmf, with the empirical proportions taken as the true population proportions. S2 FigB is similar to A, but the spike-in counts are from the yeast dilution study, and deviations from the multinomial predictions are somewhat more pronounced. Finally, S2 Fig C corresponds to data from the yeast growth rate study, in which the libraries were roughly 5 times smaller than those in the *Ciona* embryonic differentiation study (panel A) and in the yeast dilution study (panel B). In the yeast growth rate study, deviations of spike-in counts from those predicted by the multinomial model are most pronounced, for reasons we do not understand. S2 Fig A and B show that for 2 data sets with similarly large library sizes, the vast majority of the more substantial log fold differences, say those larger in magnitude than 0.15 ($\sim 10\%$ deviation), are captured within the mid 0.99 quantile range. The conclusion is that spike-in noise is accounted for to large extent by multinomial sampling noise. Nevertheless, S2 Fig A–C clearly show that the multinomial model for spike-in counts, which takes into account only sampling noise, does not capture all variation. This is no surprise because because it does not take into account poorly understood non-Poisson noise, present even in technical replicates, that was characterized by [36] and [17]. It is important to note that deviations from the multinomial model in S2 Fig A–C cannot be a reflection of any experimental errors that cause deviations from intended attomoles of spike-ins added to the RNA from a population of cells of fixed size; e.g. imprecise measurement of the volume of the aliquot from the stock spike-in mixture and dilution dilution error. Deviations from the multinomial model also cannot be a reflection of imprecise measurement of the number of cells (10^7 for yeast data and 800 for *Ciona* data) from which the cellular RNA is extracted, Nor could the deviations stem from random loss of total RNA between addition of spike-ins to cell population and final production of the sample for sequencing. None of these potential errors would effect the proportions of spike-ins within the spike-in library.

A highly discriminating way to evaluate a noise model for counts is that of [36], who plotted CV^2(mean) versus mean, on log-log axes for spike-ins (and for native RNA as

well). S2 Fig D shows such a plot for the *Ciona* spike-in data (open symbols). For each spike-in i the mean normalized is count plotted on the x-axis. For each library j, the count for spike-in i, $y_{i,j}$, is normalization is by the total spike-in count in library j, $\mathcal{L}_j^{\mathrm{SI}}$; so the normalized count is simply $y_{i,j}/\mathcal{L}_j^{\mathrm{SI}}$, the proportion of total counts in spike-in library j accounted for by spike-in i. The mean on the x-axis is over all libraries. The corresponding squared CV is plotted on the y-axis. The CV would be unchanged if the counts were normalized by ν_j instead of by $\mathcal{L}_j^{\mathrm{SI}}$, and the mean values would simply be shifted on the x-axis. The solid black line connects the theoretical population CV^2 values according to the multinomial model, and it is drawn from

$$\mathrm{CV}^2(\mu_i) \;=\; \frac{1}{n_{\mathrm{rep}}} \operatorname*{mean}_j \left(\frac{1}{\mathcal{L}_j^{\mathrm{SI}}} \right) \left[\frac{1-\mu_i}{\mu_i} \right], \tag{3}$$

where $\mu_i = p_i$, the population proportion for spike-in i. This equation is indistinguishable form the equation for Poisson noise for small values of μ_i (or p_i). The majority of the empirical CV^2 values lie above the line. Moreover, the empirical points and the theoretical line diverge for large μ values. This behavior reflects over-dispersion in the spike-in counts that was characterized by [17] and [36].

Jitter in the relative yield coefficients of the spike-ins gives jitter in their nominal abundances. If this jitter is described by a gamma probability density function, the negative binomial approximation for spike-in counts is analogous to that for native RNA. However, we expect this approximation to be less good for the spike-ins with higher proportions where the binomial marginal probability mass function, for given abundances, is not well approximated as Poisson.

The solid red line in S2 Fig D is the population CV^2 given by a negative binomial model for random spike-in counts $Y_{i,j}$, in which the mean is given by Eq (1) and the shape parameter is a single value, $a = 1000$. The corresponding theoretical CV^2 equation for the negative binomial model is

$$\mathrm{CV}^2(\mu) \;=\; \left[\frac{1}{n_{\mathrm{rep}}\, a} \right] + \left[\frac{1}{n_{\mathrm{rep}}} \operatorname*{mean}_j \left(\frac{1}{\mathcal{L}_j^{\mathrm{SI}}} \right) \right] \frac{1}{\mu}. \tag{4}$$

Eq (4) is a theoretically derived special case of the empirical equation of [36], $\mathrm{CV}^2 = \alpha_0 + \alpha_1/\mu$, also for spike-in noise. The dotted red lines (S2 FigD) demarcate the mid 0.99 quantile range of CV^2 values generated in 10,000 Monte Carlo simulations in which the synthetic spike-in counts were drawn form the negative binomial distribution above. The vast majority of the empirical CV^2 are captured within the mid 0.99 quantile range of random CV^2 values according to the negative binomial model. Furthermore, points like the empirical CV^2 values that fall outside the mid 0.99 quantile range can be generated by choosing some of the spike-in a-values to be smaller than 1000. However, we do not understand why a sizable majority of empirical CV^2 values lie above the solid red line. Our Monte Carlo simulations indicated that bias in the empirical CV^2 values (judged based on the negative binomial model) is not sufficient to account for the vertical (or horizontal) offset between the red line and the empirical CV^2 values. S2 Fig E is like the figure in panel D, but the spike-in count data are from the yeast dilution study. Finally, S2 Fig F corresponds to the yeast growth rate study, and the negative binomial spike-in model does not capture spike-in noise nearly as well as in the 2 other studies above.
