# Supplementary material for: A complete statistical model for calibration of RNA-seq counts using external spike-ins and maximum likelihood theory
Source: PLoS Comput Biol. 2019 Mar 11;15(3):e1006794. doi: 10.1371/journal.pcbi.1006794 (PMC6428340; doi:10.1371/journal.pcbi.1006794)
Supplement: S6 Appendix — (PDF) [file pcbi.1006794.s006.pdf]

Modeling of relative yield coefficients, α_i

S3 Fig B and D show plots of observe versus fitted values of α based on a mathematical model including spike-in length (nt), GC content, and folding energy. For sake of simplifying notation in the model, we suppress the subscript i on the explanatory variables: x_1, defined as the difference between length of a spike-in and that of the reference spike-in; x_2, defined as the difference between the GC content of a spike-in and that of the reference spike-in; and x_3, defined as as the difference between folding energy of a spike-in and that of the reference spike-in. The fitted values are plotted from the equation

$$\alpha \;=\; [1+\beta_1\,x_1]\,\exp\left(\,\beta_2\,x_2\;+\beta_3\,x_3\,\right). \tag{1}$$

Eq (1) says that, for fixed GC content and folding energy, the relative yield coefficient of a spike-in molecule, α, depends linearly on length. The form of the linear coefficient in square brackets is a reflection of the fact that the relative yield coefficient of the reference spike-in is equal to 1 by definition. Also note that the term in square brackets must be positive, so there is a constraint that $1+\beta_1\min(x_1)>0$. Eq (1) also says that, for fixed length, α depends exponentially on GC content, and exponentially and folding energy. This modeling is closely related to that of [17], but they modeled counts; the explanatory variables were all in the argument of an exponential function; and folding energy was not included.

We estimated the β parameters in Eq (1) by maximizing the the total log-likelihood of all observed spike-in counts when each relative yield coefficient α_i is computed from Eq (1) with the corresponding explanatory variable values, $(x_1)_i$, $(x_2)_i$, and $(x_3)_i$. This is an iterative procedure. In this maximum likelihood computation, we took into account the observation above, that, upon close inspection, the distribution of spike-in counts is closer to negative binomial than multinomial, Consequently, we used the multinomial distribution in our log likelihood function. For each spike-in i in library j, its expected count (negative binomial mean) $\mu_{i,j}$ is given by

$$\mu_{i,j}\;=\;\frac{\alpha_i n_i}{\sum\limits_{k=1}^{s}\alpha_k n_k}\,\mathcal{L}_j^{\mathrm{SI}}, \tag{2}$$

where the α_i in each iteration are computed from the β-values in that iteration. We took the negative binomial shape parameter to be $a=1000$, based on the analysis of spike-in noise above. The part of the log likelihood (LL) of spike-in count $y_{i,j}$ that depends on the β-coefficients, through $\mu_{i,j}$ in Eq (2), is given by

$$\mathrm{LL}(y_{i,j})\;=\;-(a+y_{i,j})\,\log(\mu_{i,j}+a)\;+\;y_{i,j}\,\log(\mu_{i,j}) \tag{3}$$

The total log likelihood is given by summing log likelihoods in Eq (3) over i and j. We minimized -(total log likelihood) over the β-values using R's built-in *nls* function. We found all 3 explanatory variables — length, GC content and folding energy — to be highly significant by 3 separate log ratio of maximum likelihoods tests. In each test, the null model was a model in which one of the 3 explanatory variables was omitted, while the alternative model was the full model. The maximum likelihood estimates of the β-coefficients are reported in the S3 Fig.

Spike-in proportions computed from the *Ciona* and yeast data are highly correlated ($r=0.85$ for proportions, and $r=0.92$ for log proportions), but the corresponding α-values are not tightly correlated ($r=0.27$ for α-values, and $r=0.30$ for log α-values). This dichotomy between the two correlation coefficients seems to be a consequence of the fact that, according to S1 Appendix Eq(10), α_i depends on the ratio of corresponding proportions and attomoles, f_i/n_i. While f_i and n_i are highly correlated

($r = 0.89$ and $r = 0.94$ on linear and log scales, respectively, for dilution data; $r = 0.96$ and $r = 0.95$ on linear and log scales, respectively, for *Ciona* data), the quotient is sensitive to the departures of f_i from a linear dependence on n_i.
