# Supplementary material for: A complete statistical model for calibration of RNA-seq counts using external spike-ins and maximum likelihood theory
Source: PLoS Comput Biol. 2019 Mar 11;15(3):e1006794. doi: 10.1371/journal.pcbi.1006794 (PMC6428340; doi:10.1371/journal.pcbi.1006794)
Supplement: S7 Appendix — (PDF) [file pcbi.1006794.s007.pdf]

Determination of shape parameter a in the yeast growth rate study

We explore a simple statistical model for yeast with as single shape parameter a across all transcripts, as advocated by [47], within each condition. To determine the negative binomial shape parameter a_l (for condition l) we used a variation on the maximum marginal likelihood estimator of [11]. In this method, the marginal likelihood of a particular set of counts for transcript i (across replicates) in condition l, $y_{i,j\in\Omega_l}$, given a, is found by integrating over μ the likelihood (probability) of those counts, given a and μ, multiplied by the conditional probability density function for μ given a:

$$\Pr\{y_{i,j\in\Omega_l}\,|\,a\} \;=\; \int_0^\infty \prod_{j\in\Omega_l} (\mathrm{NB}(y_{ij};\nu_j\mu_Z,a) f_{\mu_Z|a}(\mu_Z;a)\,d\mu_Z, \tag{1}$$

where $\Pr\{y_{i,j\in\Omega_l}\,|\,a\}$ is the the joint marginal probability mass function for $y_{i,j\in\Omega_l}$ given a, as defined on the right-hand side of Eq (1); $\mathrm{NB}(y_{ij};\nu_j\mu_Z,a)$ is the negative binomial probability of spike-in count $y_{i,j}$ with mean parameter $\nu_j\mu_Z$ and shape parameter a; and $f_{\mu_Z|a}(\mu_Z;a)$ is the conditional probability density function for random μ_Z, given the shape parameter a. For the conditional density function $f_{\mu_Z|a}(\mu_Z;a)$, [11] chose a conjugate prior distribution for the negative binomial, which allows analytical integration of Eq (1) in the special case of constant ν across replicates j in condition l. We implemented the method of [11], but we chose to use the empirical density function for μ_Z instead of the conjugate prior. Either way the integration must be done numerically when ν_j varies over replicates within a condition.

To determine a single shape parameter for each condition l, we maximized the sum of the log marginal likelihoods over all transcripts within each condition:

$$a_l^{\mathrm{MML}} \;=\; \arg\max_a \sum_{i=s+1}^{s+q} \log \Pr\{y_{i,j\in\Omega_l}\,|\,a\} \tag{2}$$

We obtained a-values of 23, 34, and 24 for growth rates per cell of 0.12, 0.20, and $0.30\,\mathrm{h}^{-1}$. These a-values are somewhat smaller than the $a \approx 100$ (across all transcripts) of [47].

We checked this method for estimating the shape parameter a, using the empirical density, on synthetic (Monte Carlo) data generated according to our statistical model for each condition l. For each RNA species, in each library, with observed count $y_{i,j}$ we generated a corresponding random count according to the multinomial model, with a fixed a for each condition. We found the recovered a-values to be within 10% of but always less than the actual values.

Test of statistical model for yeast: comparison of variation in laboratory, and synthetic data

To the extent that our statistical model for RNA is a good one, our model, when used in a generative mode, should produce synthetic data that is like our laboratory data.

For each RNA transcript, in each library, with observed count $y_{i,j}$ we generated a corresponding random count, $Y_{i,j}^{\mathrm{MC}}$ according to the negative binomial model, and computed a corresponding random abundance, $Z_{i,j}^{\mathrm{MC}}$ according to our ν_j calibration method. The Monte Carlo count corresponding to $y_{i,j}$ for j in condition l is a negative binomial random variable with mean ν_j times the mean, over j in condition l, of $z_{i,j}$, and shape parameter for condition l, a_l, as determined by the maximum marginal likelihood method for condition l (Eq (2)).

One way to judge the similarity between data types is by comparing distributions of the sample estimate of coefficient of variation of the mean for our laboratory data and for the Monte Carlo (synthetic) data, as in S4 Fig.
