# Supplementary material for: A complete statistical model for calibration of RNA-seq counts using external spike-ins and maximum likelihood theory
Source: PLoS Comput Biol. 2019 Mar 11;15(3):e1006794. doi: 10.1371/journal.pcbi.1006794 (PMC6428340; doi:10.1371/journal.pcbi.1006794)
Supplement: S8 Appendix — (PDF) [file pcbi.1006794.s008.pdf]

Comparison with other normalization methods

Anders and Huber-like spike-in normalization

Our normalization method is most closely similar to that of [36] who adapted and applied the library size scaling factor [34] s_j in Eq (5) in the present paper to put RNA sequencing counts on a common scale by using spike-ins. In the method of [36], the computation of s_j is done with spike-in counts only. For the sake of clarity, let's distinguish the spike-in normalization factor of [36] from that of [34] (without spike-ins) by naming it, s_j^{SI}. An important distinction between the s_j^{SI} scaling of counts and ours is that our nominal cellular RNA abundances, obtained the scaling $z_{i,j} = y_{i,j}/\nu_j$, are a step closer to absolute RNA abundances, which we have called $n_{i,j}$. Nominal and absolute abundances differ by a relative yield scale factor, α_i; i.e., $z_{i,j} = \alpha_i \, n_{i,j}$. A nominal abundance of ours $z_{i,j}$ can be converted to an absolute abundances in the future once the relative yield coefficient is measured. There are a number of was that one could measure relative yield coefficients (see Discussion). In contrast, the s_j^{SI} scaling of counts produces a dimensionless measure of abundance that has no direct relationship to underlying cellular molecular abundance (Discussion).

What is the quantitative relationship between the library size factor, s_j^{SI}, of [36], computed using only spike-in counts, and our normalization factor ν_j? We show below that s_j^{SI} and our ν_j are approximately proportional, by a scaling parameter, universal across libraries, in a given study, when the spike-in library sizes are large enough. To make this clear, we rewrite the formula for s_j^{SI} in terms of spike-in library sizes $\mathcal{L}_j^{\mathrm{SI}}$ and fractions of total spike-in library counts accounted for for spike-in i in library j, $f_{i,j}$, as follows.

$$
\begin{aligned}
s_j^{\mathrm{SI}} &= \underset{i \subset \{1,2,\ldots,s\}}{\text{median}} \frac{y_{i,j}}{\left(\prod_{k=1}^{r} y_{i,k}\right)^{1/r}} \\
&= \underset{i \subset \{1,2,\ldots,s\}}{\text{median}} \frac{f_{i,j}\, \mathcal{L}_j^{\mathrm{SI}}}{\left(\prod_{k=1}^{r} f_{i,k}\, \mathcal{L}_j^{\mathrm{SI}}\right)^{1/r}} \\
&= \underset{i \subset \{1,2,\ldots,s\}}{\text{median}} \left[\frac{f_{i,j}}{\left(\prod_{k=1}^{r} f_{i,k}\right)^{1/r}}\right] \frac{\mathcal{L}_j^{\mathrm{SI}}}{\left(\prod_{m=1}^{r} \mathcal{L}_m^{\mathrm{SI}}\right)^{1/r}}
\end{aligned}
\tag{1}
$$

(Note that the median in Eq (1) must be computed over the subset of spike-ins that are detected in all libraries; otherwise, the geometric mean in the denominator is equal to zero.) For large spike-in library sizes, the spike-in fractions $f_{i,j}$ differ little from population spike-in proportions p_i, whose maximum-likelihood estimators are f_i in Eq (8), as we have shown above. Therefore, for large spike-in library sizes, the term is square brackets in Eq (1) is close to 1. Consequently, multiplying s_j^{SI} in Eq (1) by a particular global scale factor gives "library-size" factors, which we call $\tilde{s}_j^{\mathrm{SI}}$ that are close

to our ν_j:

$$\begin{aligned}\tilde{s}_j^{\mathrm{SI}} &\stackrel{\mathrm{def}}{=} \left\{ \left(\prod_{m=1}^{r} \mathcal{L}_m^{\mathrm{SI}} \right)^{1/r} \frac{n_1}{f_1} \right\} s_j^{\mathrm{SI}} \\ &= \underset{i \subset \{1,2,\ldots,s\}}{\mathrm{median}} \left[\frac{f_{i,j}}{\left(\prod_{k=1}^{r} f_{i,k} \right)^{1/r}} \right] \frac{n_1}{f_1} \mathcal{L}_j^{\mathrm{SI}} \\ &\approx \frac{n_1}{f_1} \mathcal{L}_j^{\mathrm{SI}} = \nu_j. \end{aligned} \tag{2}$$

Our computations confirm this near equality in the case of the *Ciona* embryonic differentiation data and in the case of the yeast dilution-study.

In the *Ciona* embryonic differentiation study, the ranges of summed counts for RNA, spike-in, and total libraries were 9.8–14 , 0.58–1.9, and 10–16×10^6, respectively. In this study, we found the ratio of the 2 normalization constants to be nearly equal to 1; i.e., $0.983 < \tilde{s}_j^{\mathrm{SI}}/\nu_j < 1.03$. An implication is that the library size factor s_j^{SI} of [36], computed with spike-ins only in Eq (1), is consistent with our ν_j normalization factor that is the result of a maximum likelihood derivation. Furthermore, the scaling of their s_j^{SI}, as in Eq (2) gives nominal abundances that are virtually identical to ours for the *Ciona* data.

We confirmed that pairwise testing of differential gene expression with s_j^{SI} size factors in place of our ν_j values yields very similar results (with or without the δ correction factors).

In the yeast dilution study, the ranges of summed counts for RNA, spike-in, and total libraries were 8.5–12 , 0.65–2.4, and 10–13×10^6, respectively. We computed ν_j and $\tilde{s}_j^{\mathrm{SI}}$ values for each of 6 libraries and found all the $\tilde{s}_j^{\mathrm{SI}}/\nu_j$ ratios (0.994, 1.00, 0.987, 1.00, 1.01, 1.01) to differ by less than 1.3% . Consequently the abundances of RNA computed by the two normalization methods are virtually identical. The conclusion is that the inferred abundances of RNA and spike-in for the technical replicates in this dilution study are essentially exactly the same for the ν_j and $\tilde{s}_j^{\mathrm{SI}}$ normalization methods.

In the case of our yeast GR data, with library sizes roughly 5 times smaller than those above, the $\tilde{s}_j^{\mathrm{SI}}$ and ν_j normalizations methods differed somewhat. The ranges of summed counts for RNA, spike-in, and total libraries were 1.6–2.6 , 0.14–0.42, and 1.8–3.0×10^6, respectively. For these data we found the $\tilde{s}_j^{\mathrm{SI}}/\nu_j$ ratios (1.28, 1.22, 1.38, 1.05, 0.850, 0.978, 0.954, 0.819, 0.840) to vary from -18% to +38%.

The ratios of scale factors, $\tilde{s}_j^{\mathrm{SI}}$ and ν_j, average over replicates within each condition are 1.30, 0.958, and 0.870 for growth rates per cell of 0.12, 0.20 and 0.30 h^{-1}, respectively. A practical implication with regard to estimating RNA abundance in our GR study in yeast is that the $\tilde{s}_j^{\mathrm{SI}}$ normalization method introduces a mean fold difference of 1.5 over and above the fold difference between growth rates per cell of 0.30 and 0.12 h^{-1} given by the ν_j normalization method. The difference remains if a common set of spike-ins is used to compute both normalization constants, namely, all spike-in molecules that are detected in all libraries. Perhaps differences are a consequence of technical noise in this study (S2 Fig C) that we do not understand.

We wondered whether the differences between the $\tilde{s}_j^{\mathrm{SI}}$ and ν_j normalization constants, when they exist, are a consequence of the $\tilde{s}_j^{\mathrm{SI}}$ normalization method giving relatively more weight to noisy counts from spike-ins with low abundance. This possibility is supported by recomputing the $\tilde{s}_j^{\mathrm{SI}}$ and ν_j normalization constants based solely on the counts from subsets of spike-in molecules accounting for large proportions of the counts, the top 5 (78% of total), the top 10 (90% of total), or the top 15 (96% of total) to obtain $\tilde{s}_j^{\mathrm{pruned}}$ and ν_j^{pruned}. We found all of the ν_j^{pruned} values to be very close

to the original ν_j values. But the $\tilde{s}_j^{\text{pruned}}$ values differ from the original $\tilde{s}_j$ values in such a way that all are close to the corresponding ν_j^{pruned} and original ν_j values. For example, when the top 5 spike-in molecules are used, the new $\tilde{s}_j^{\text{pruned}}$ and ν_j^{pruned} differ by less than 3%. Results are closely similar if the top 10 or the top 15 spike-in molecules are used for the normalization instead.

Is it possible that the discrepancy between the $\tilde{s}_j^{\text{SI}}$ and ν_j normalization constants is due instead to the ν_j normalization method giving undue weight to the top 1 or 2 spike-in molecules that account for a large fraction of the total spike-in counts? Perhaps the concordance between the $\tilde{s}_j^{\text{SI}}$ and ν_j normalizations using a top subset of spike-in molecules is a consequence of both methods being dominated by the top 1 or 2 spike-in molecules in this situation. We checked this possibility by pruning the spike-ins from the top rather than the bottom to compute the new (pruned) normalization constants. When we removed the top spike-in accounting for 27% of all spike-in counts, and when we removed the top 2 spike-ins accounting for 49% of all spike-in counts, the discrepancy between the two normalization methods was little changed.

The conclusion is that the differences between the ν_j and $\tilde{s}_j^{\text{SI}}$ normalization constants for the yeast GR data stem from the $\tilde{s}_j^{\text{SI}}$ normalization method giving substantially more weight to the spike-in molecules of low abundance that account for less than 5% of the total spike-in counts. Although the $\tilde{s}_j^{\text{SI}}$ normalization method is sensitive to noisy spike-in of low abundance, this does not imply that the the ν_j normalization is superior. The more accurate negative binomial model above for technical spike-in noise with very large size factor (~ 400, mimicking slight jitter in the relative yield coefficients) could be helpful in further analysis of this issue.

Correction factor $\boldsymbol{\delta_j}$ and its relationship to the total cellular RNA scale factor, $\boldsymbol{\xi_j}$, in the single-cell RNA-seq study of [36]

Our δ_j correction factor in S2 Appendix Eq (4) is closely related to the ξ_j correction factor in the single-cell RNA-seq study of [36]. In that study, the authors regard s_j^{SI} as a scale factor that accounts for the effect of sequencing depth on the expected value of observed counts corresponding to each RNA molecule in library j. In addition, they argue, the expected count is scaled by a factor ξ_j that accounts for an unwanted source of variation, namely, differences in total RNA across samples as a consequence of cell lysis efficiency and true differences in total cellular RNA. Thus, in their view, the expected count for transcript i in library j is given by

$$\mathbb{E}[Y_{i,j}] \;=\; \xi_j \, s_j^{\text{SI}} \mu_i, \tag{3}$$

where μ_i is the expected (geometric) average count across libraries. Consequently, a point estimate intrinsic abundance of RNA molecule i in sample j, uncontaminated, so to speak, by sequencing depth and total RNA variation, is given by $y_{i,j} / \left(\xi_j \, s_j^{\text{SI}}\right)$. In this model, it follows, as the authors point out, that ξ_j can be estimated by

$$\xi_j \;=\; \frac{s_j^{\text{B}}}{s_j^{\text{SI}}}, \tag{4}$$

where s_j^{B} is their "biological" size factor, i.e., the median-based size factor in Eq (5), based on counts from native RNA molecules.

If one rewrites the S2 Appendix Eq (4) for δ_j by substituting, on the right-hand side, $y_{i,j}/\nu_j$ for $z_{i,j}$, and one uses the relationship between ν_j and s_j^{SI} above, and the definition of s_j^{B}, one finds that

$$\delta_j \;\approx\; \xi_j, \tag{5}$$

with equality in the idealized case in which the spike-in counts are equal to their expected values.

Although the correction factor ξ_j of [36] and our δ_j correction factor are essentially the same mathematically, our philosophy behind δ_j differs slightly from that of [36]. We think of our δ_j as a correction for unwanted variation that could stem from unintentional variation of spike-in amount and/or variation in total RNA (within a condition) due to errors in total cell count and/or lysis efficiency. In contrast, [36] think of their spike-in amounts as exactly equal to the intended amount, with variation only in the total RNA among samples. In the end, we normalize counts in a smilar way with regard to the use of correction factors; i.e.

$$y_{i,j} / \left(\xi_j \, s_j^{\mathrm{SI}}\right) \;=\; y_{i,j}/s_j^{\mathrm{B}}$$

for [36], and

$$y_{i,j} / \left(\delta_j \, \nu_j\right)$$

for us.

Removal of unwanted variation (RUV) methods

[15, 41, 43, 44] use factor analysis to identify and remove unwanted variation in inferred gene expression stemming from nuisance sources, which could be unidentified. The authors have demonstrated that these methods are very effective when they are applied with care and judgement. Three versions of RUV that are available as R functions, RUVg, RUVs, and RUVr, in the R package RUVSeq [15]. As the authors have pointed out, RUV-normalized counts are pseudo-counts and they are for diagnostic purposes only; they give a hint if one is on the right track in applying these methods. The power of RUV methods comes from following them with regression and hypothesis testing. Any of these methods can be applied to RNA-seq count data following ν_j (or s_j^{SI}) normalization. Two, RUVg, RUVs, seem of particular interest, because they can both be applied to a set of control genes or spike-ins, to reduce variation in the RNA expression of interest. However, these two RUV methods were designed for application in situations where only a small fraction of genes are differentially expressed, and total cellular RNA varies little. Neither of these methods by itself, or following global normalization (e.g. upper quartile (UQ) or median (s_j) normalization), was proposed to detect and quantify genome-wide amplification of expression, and they are not suited to do so [19, 41]. The simple reason is that, in the case of large variation of total RNA driven by treatment/condition/cell-type, the variation in spike-in counts is strongly correlated with variation in the RNA counts. This is especially evident if one considers the special case of a fixed total RNA-seq library size. A fundamental condition that must be satisfied for the application for an RUV method based on an invariant gene set or spike-ins to be useful is that the variation in counts from the control genes or spike-ins be uncorrelated with the variation of RNA driven by biological factors of interest. A more mathematical explanation based on a simple heuristic example follows.

The RUV methods are based on a linear population model for the log of expected normalized counts,

$$\log\left(\mathbf{D}^{-1}\,\mathbb{E}[\mathbf{Y}^T]\right) \;=\; \mathbf{X}\boldsymbol{\beta} + \mathbf{W}\boldsymbol{\alpha}, \tag{6}$$

where $\mathbf{Y}$ is the genes-by-libraries matrix of counts, $\mathbf{D}$ is a diagonal matrix of library-specific normalization (size) factors (determined in a separate independent step preceding RUV normalization), $\mathbf{X}$ is the libraries-by-conditions design matrix, $\boldsymbol{\beta}$ is a conditions-by-genes matrix of regression coefficients, $\mathbf{W}$ is the libraries-by-k factor matrix identified (estimated) by the RUV method, k is the assumed number of factors contributing to unwanted variation, and $\boldsymbol{\alpha}$ is a k-by-genes matrix of regression

coefficients. The assumption underlying an application of an RUV method using spike-in controls is that the variation in spike-in counts is unrelated to the explanatory variables (treatment). This is not the case with global gene amplification. Consider a heuristic, idealized situation in which treatment causes all RNA counts to increase by a factor of e^b, and spike-in counts to decrease by a factor of e^a, compared to control, and there is just a single control library and a single treatment library. In this case, the factor matrix $\mathbf{W}$, which accounts for deviation of log spike-in counts from the mean, with $k = 1$, is given by $\mathbf{W} = [a/2, -a/2]^T$; and, in this case, the null model, in which one attempts to account for all variation in RNA counts by factor matrix $\mathbf{W}$ only in Eq (6), accounts fully for the variation. If we define $\mathbf{y}$ to be the 2-element vector of counts for a particular RNA molecule, one for control and one for treatment, then we can express $\mathbf{y}$ exactly in terms of the null model,

$$\begin{aligned} \log \mathbf{y} &= \begin{bmatrix} \log y_0 \\ \log y_0 \end{bmatrix} + \begin{bmatrix} 0 \\ b \end{bmatrix} \\ &= \begin{bmatrix} \log y_0 + b/2 \\ \log y_0 + b/2 \end{bmatrix} + \begin{bmatrix} a/2 \\ -a/2 \end{bmatrix} (-b/a) \\ &= \begin{bmatrix} 1 \\ 1 \end{bmatrix} \beta_0 + \mathbf{W}\alpha, \end{aligned} \tag{7}$$

where $\beta_0 = \log y_0 + b/2$, $\mathbf{W} = [a/2, -a/2]^T$ (as defined above), and $\alpha = -b/a$. The erroneous conclusion, following from failure to reject the null model, is that there is no "real" effect of treatment on gene expression. In contrast, if RNA counts are normalized by our maximum likelihood, ν_j, method, the conclusion is that treatment leads to a log fold change of $b + a$, which is the correct conclusion in the context of our statistical model.

Comparison of δ_j correction with the RUVr method

Because RUV normalization does not normalize for total cellular RNA, if it is used, it should be applied on top of ν_j normalization. S5 Fig A demonstrates the results of applying an RUVr method with 1 factor of unwanted variation (RUVSeq R package [15]) following ν_j normalization. Data are from the yeast growth rate study. The RUVr method uses a factor matrix $\mathbf{W}$ computed by modeling the residuals from the linear model in Eq (6) without the factor matrix $\mathbf{W}$.

Our δ_j correction correction method is very restricted and constrained compare to the RUVr method. With the removal of 1 factor of unwanted variation, the W matrix in 6 is simply a column vector. The transpose of $\mathbf{W}$ is something akin to our vector of $\log \delta$ values. But there is an important difference. Our δ vector is is applied as a normalization factor to every row (transcript) in the the (genes $\times$ replicates) RNA count matrix In the RUVr method, every row of counts is normalized by its own multiple α value in Eq 6. Consequently, if there are q RNA molecules, the RUVr method has q more fitted parameters than the δ_j method. It is worth knowing, for the sake of interpreting the results below, that the δ_j correction method above is a special case of RUVr with $k = 1$; it corresponds to $\mathbf{W} = \pm \log \boldsymbol{\delta}$ and a very particular outcome in which every element of the 1-by-genes $\boldsymbol{\alpha}$ matrix equal to either plus 1 or minus 1 in Eq (6) (see text of S5 Fig on the observed near equality). The equality, $\mathbf{W} = \pm \log \boldsymbol{\delta}$ obtains when the RNA abundance vectors $\mathbf{zi_j}$ are equal except for global scale factors δ_j for libraries within a condition l. The RLE plot S5 Fig A for RUVr normalization of of our ν_j normalized counts is similar to the one obtained by correcting for putative library preparation errors with library specific δ_j scaling factors in Fig S1 Fig D. In these plots there are clear differences in the 0.5 quantile of relative log error across growth rates that reflect genome-wide amplification of expression with increasing

growth rate. The corresponding principal components analysis (PCA) biplot of the normalized scores of the second, versus the first principal component, based on the normalized counts matrix, is shown in S5 Fig B. Each of the 3 clusters contains libraries from only one condition. RUVr-normalized counts in the RLE and PCA plots are pseudo-counts and meant for diagnostic purposes only. Hypothesis testing is performed on the original counts using the population model in Eq (6). For the yeast GR data, we found little difference between the RUVr normalization and δ_j correction for comparing the lowest to the highest growth rate. Among significantly regulated genes (FDR=0.01), 3,835 were detected by both methods; the RUVr and δ_j methods produced only 183 and 20 additional transcripts that were not shared, respectively.

S5 Fig C is similar to panel A, but the data are from the *Ciona* embryonic differentiation study. These RLE plots are similar to those in Fig S1 Fig F for the same original counts, first normalized by ν_j, and then corrected by δ_j to account for library preparation errors. The corresponding PCA biplot (panel D) suggests imperfect clustering of the libraries, and this is confirmed by both hierarchical and kmeans clustering, in which the library labeled lacz2 is misplaced into a cluster containing 3 libraries with camras labels. In the case of the *Ciona* data, RUVr normalization and the δ_j correction factors, produce somewhat different results with respect to detection of differential gene expression. Among significantly regulated genes (FDR=0.01), in the $\mathrm{Fgfr}^{\mathrm{DN}}$/LacZ comparison, 3,848 were detected by both methods; the RUVr and δ_j methods produced 114 and 1,086 additional transcripts that were not shared, respectively. In other words, only 75% of transcripts called significantly differentially expressed by the δ_j correction method were among those found by the RUVr method.

How important is the δ_j correction, or the RUV normalization, following ν_j normalization, with respect to detection of differential gene expression? In the yeast GR study, omitting the δ_j correction (or RUVr normalization) causes a mild reduction of 20% in the number of genes called differentially expressed (FDR=0.01) between growth rates of 0.30 and $0.12\,\mathrm{h}^{-1}$ (3,048 vs. 3,855). However, in the case of the *Ciona* data, the correction for unwanted variation makes a large difference for the $\mathrm{Fgfr}^{\mathrm{DN}}$ vs. LacZ comparison. Without it, only 112 transcripts are called as differentially expressed (FDR=0.01), as opposed to 4,934 with the δ_j correction, and 3,962 with the RUVr method [15]. Thus both the RUVr and the δ_j methods for removal of unwanted variation reveal substantial regulation of absolute RNA abundance that is obscured by unwanted variation.
