# Supplementary figures and images for: A complete statistical model for calibration of RNA-seq counts using external spike-ins and maximum likelihood theory
Source: PLoS Comput Biol. 2019 Mar 11;15(3):e1006794. doi: 10.1371/journal.pcbi.1006794 (PMC6428340; doi:10.1371/journal.pcbi.1006794)

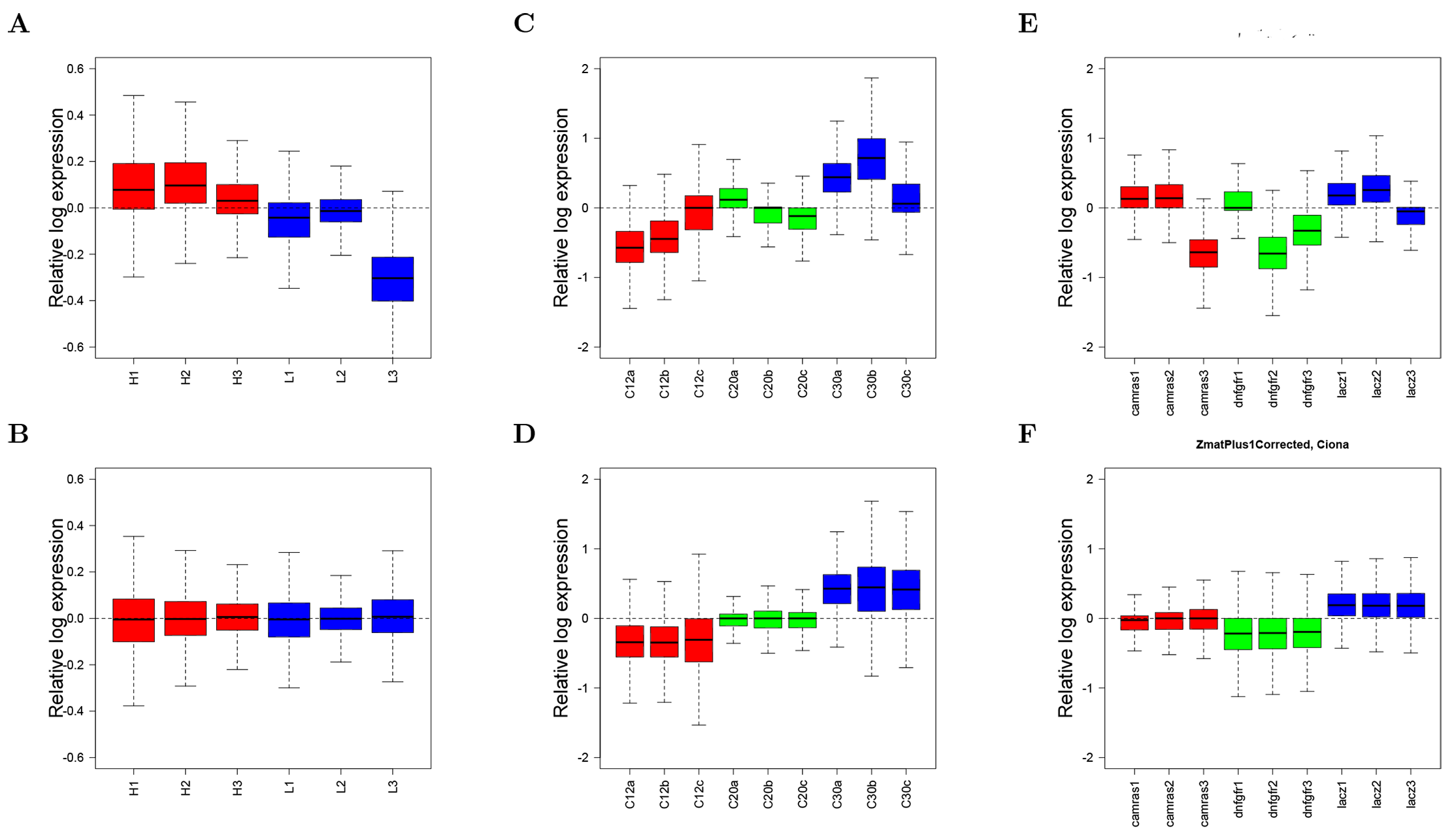

Supplement: S1 Fig — (A) RNA abundance, as estimated by the νj maximum-likelihood normalization method, in pilot studies with 6 technical replicate RNA libraries and 2 different volumes of the stock spike-in mixture added to the RNA, 3 with high (H) volume and 3 with low (L) volume. Actual RNA abundance does not vary across replicates. Variation in the medians of the distributions of relative log expression probably reflect technical volume/dilution errors in adding spike-ins to the cellular RNA and possible within-condition errors in total RNA due to cell count or RNA extraction. Before computing relative log expression, the table of counts was filtered to include only rows corresponding to those transcripts that were detected in more than 4 of 6 libraries (3 replicates for each of 2 spike-in volume aliquots). Next, the value 1 was added to each count (y˜i,j=yi,j+1) to ensure that the log of each corresponding abundance exists. Log transformation normalized counts z˜i,j=y˜i,j/νj (nominal abundance) followed. (B) Relative log expression for data in panel A, but after adjustment of abundances by a single scale factor for each library to correct for putative library preparation errors. Corrected abundances were computed by z˜i,jc=z˜i,j/δj, which is equivalent to z˜i,jc=y˜i,j/(νjδj). See text in S2 Appendix for discussion and S2 Appendix Eq (3) used to compute δj scale factors. The vector of δ-values is (1.13 1.14 1.05 0.978 1.01 0.744). (C) RNA abundance (z-values, computed by the νj maximum likelihood method) measured at 3 different growth rates per cell in yeast growth-rate/quiescence study before correcting for library preparation errors. Median values were computed across all libraries. Within each condition there is variation in location of the 0.5 quantile of RLE. The between condition variation reflects different overall RNA abundances at the growth rates per cell of 0.12, 0.20, and 0.30 h-1. (D) RLE plots for yeast GR data in panel C, but after adjustment of abundances by the δj s [file pcbi.1006794.s009.tif]

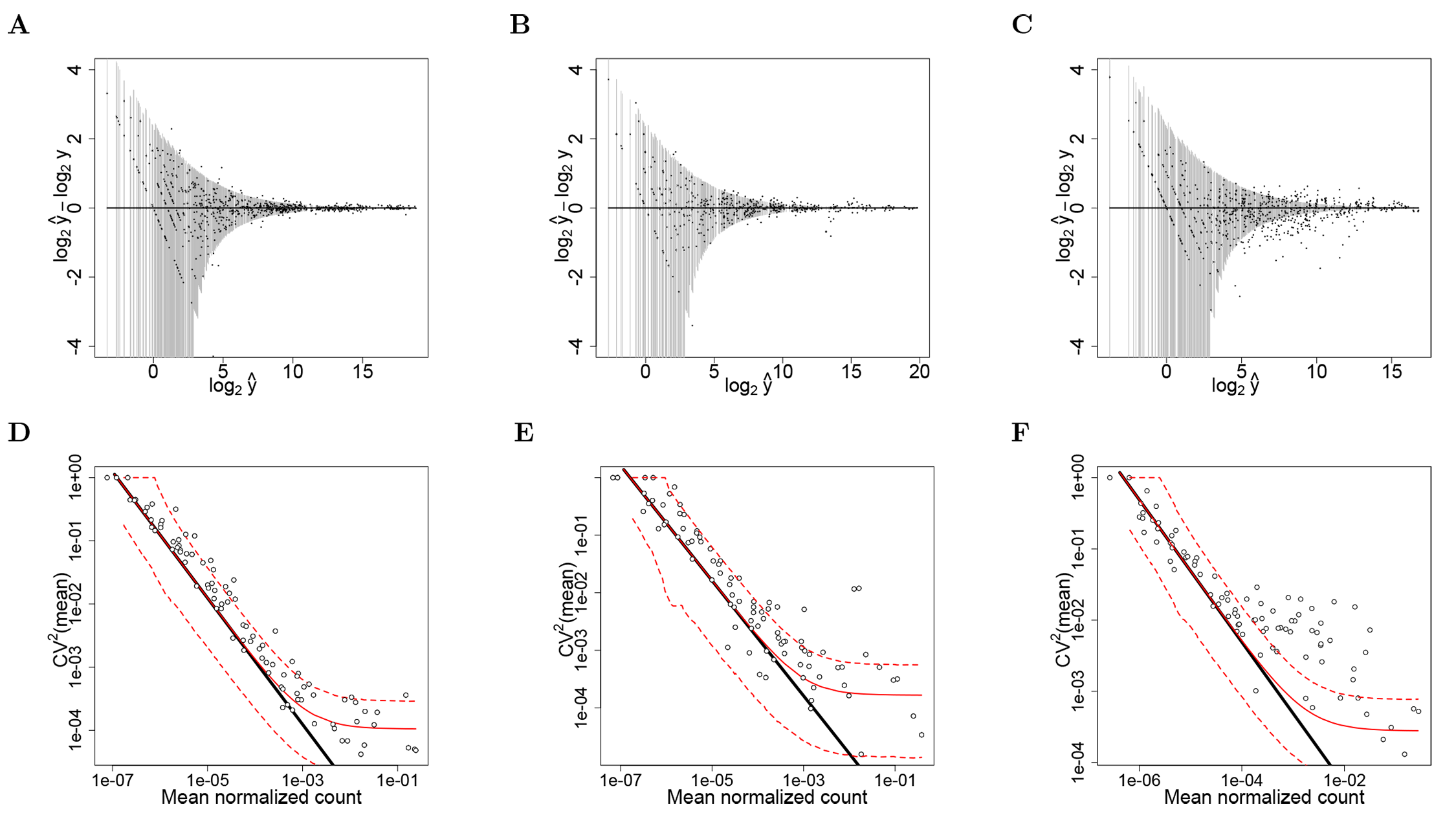

Supplement: S2 Fig — (A) Comparison of observed and predicted counts, in MA-like plot format, according to the multinomial statistical model linking spike-in counts to abundance in the corresponding sample. Each plotted point represents log2 ratio of observed to predicted spike-in counts for each detected molecule i in one or more replicates j vs. log2 of the predicted counts. Data from the Ciona embryonic differentiation study. The mean log2 ratio of observed to predicted spike-in counts is equal to 0.046, which is consistent with unbiased prediction. Vertical bars with lower and upper endpoints (L, U) demarcate a mid 0.99 quantile range of random counts generated from the multinomial model with the maximum likelihood proportions, such that Pr{Y < L} < 0.005, and Pr{Y > U} < 0.005. Because the marginal probability mass functions are binomial, the (L, U) interval for each transcript is the same as the mid 0.99 binomial quantile for that transcript. (B) Comparison of observed and predicted counts from multinomial spike-in model, as in panel A, but based on data from the yeast dilution study. (C) Comparison of observed and predicted counts from multinomial spike-in model, as in panel A, but based on data from the yeast growth rate study. (D) CV2(mean) versus mean for normalized spike-in counts, on log-log axes, for the same Ciona spike-in data (open symbols) in panel A. For each spike-in i the mean normalized count plotted on the x-axis is the mean over all libraries j of yi,j/LjSI. The corresponding squared CV is plotted on the y-axis. The solid black line connects the theoretical population CV2 values according to the multinomial model, and it is drawn from S5 Appendix Eq (3). The solid red line is the theoretical population CV2 that follows from a negative binomial model for random spike-in counts Yi,j, in which the mean is given by S5 Appendix Eq (1) and the shape parameter is a single value, a = 1000. It is drawn from S5 Appendix Eq (4). The dotted red lines demarcate the mid 0.99 qu [file pcbi.1006794.s010.tif]

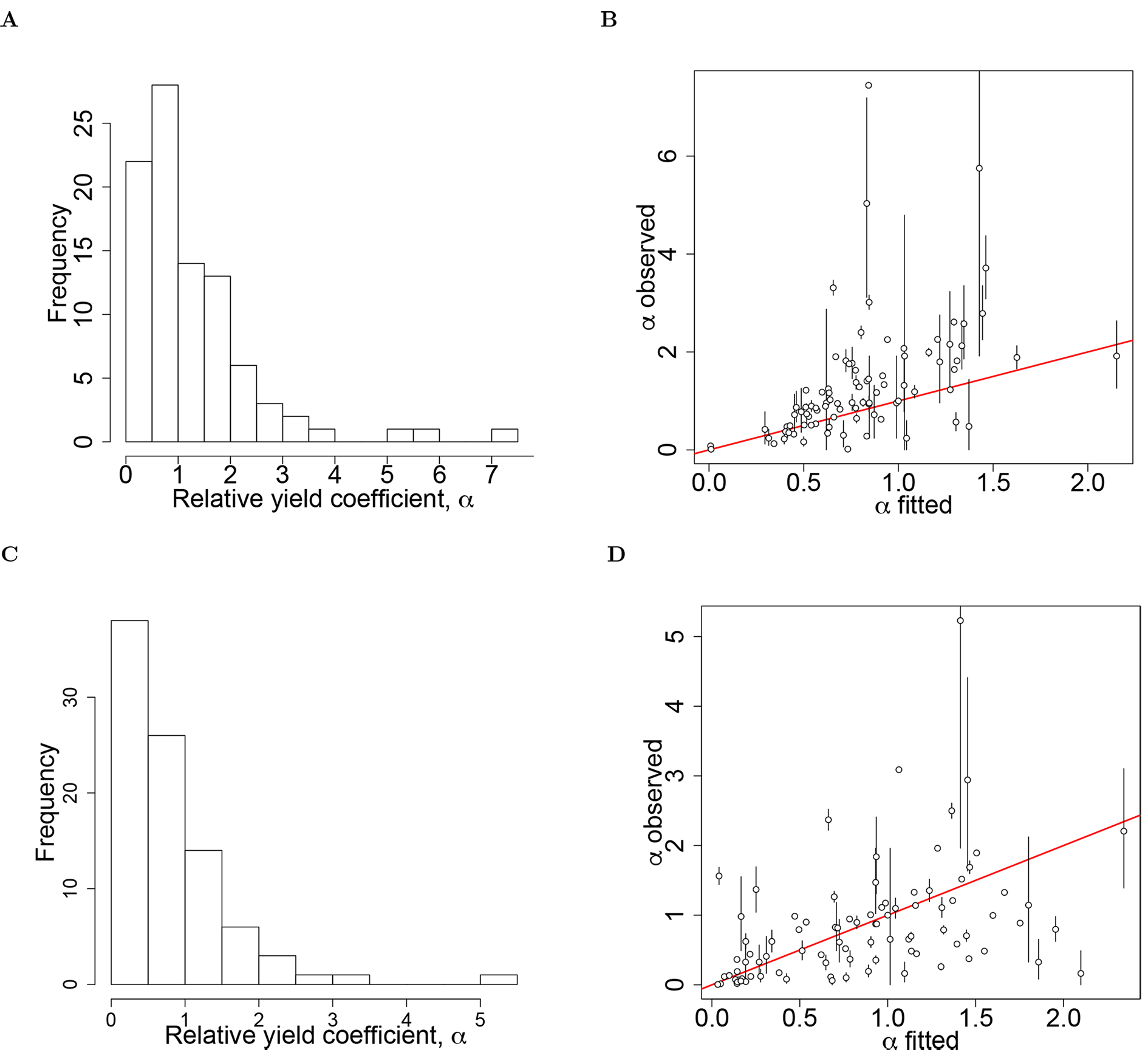

Supplement: S3 Fig — (A) Histogram of relative yield coefficients (αi) based on spike-in counts from 9 libraries in the Ciona embryonic differentiation study. The median α-value is 0.95, and the interquartile range (IQ) of 1.3 extends from 0.51 to 1.8. (B) Observed (and fitted values of αi, in the Ciona embryonic differentiation study, based on a mathematical model (S6 Appendix Eq (1)) including spike-in length (nt), GC content, and folding energy (Kcal/mol). The vertical bars delineate the mid 0.95 quantile of α-values, computed according to the multinomial model when the empirical proportions for the spike-ins are used as stand-ins for the true population proportions. In some cases, the filled symbol obscures exceptionally narrow mid 0.95 quantile ranges of the αi values. The maximum likelihood values of the β-coefficients (see text) in S6 Appendix Eq (1) are: β1 = 0.013; β2 = 0.0032; and β3 = −0.0014. The root mean square error, normalized by max(α) − min(α), or normalized by sd(α) are equal to 0.16 or 0.98, respectively. (C) Histogram of relative yield coefficients (αi) based on spike-in counts from 6 libraries in the yeast dilution study (different library preparation protocol than that used in the Ciona embryonic differentiation study). The median of α-value is 0.65, and the IQ range of 0.83 extends from 0.28 to 1.1. (D) Observed (values in panel C) and fitted values of α, for yeast dilution study, based on S6 Appendix Eq (1). The maximum likelihood values of the β-coefficients S6 Appendix Eq (1) are: β1 = 0.0012; β2 = 0.0045; and β3 = 0.140. All 3 variables were found to be highly significant (see text). The root mean square error, normalized by max(α) − min(α), or normalized by sd(α) are equal to 0.14 or 0.93, respectively. (TIF) [file pcbi.1006794.s011.tif]

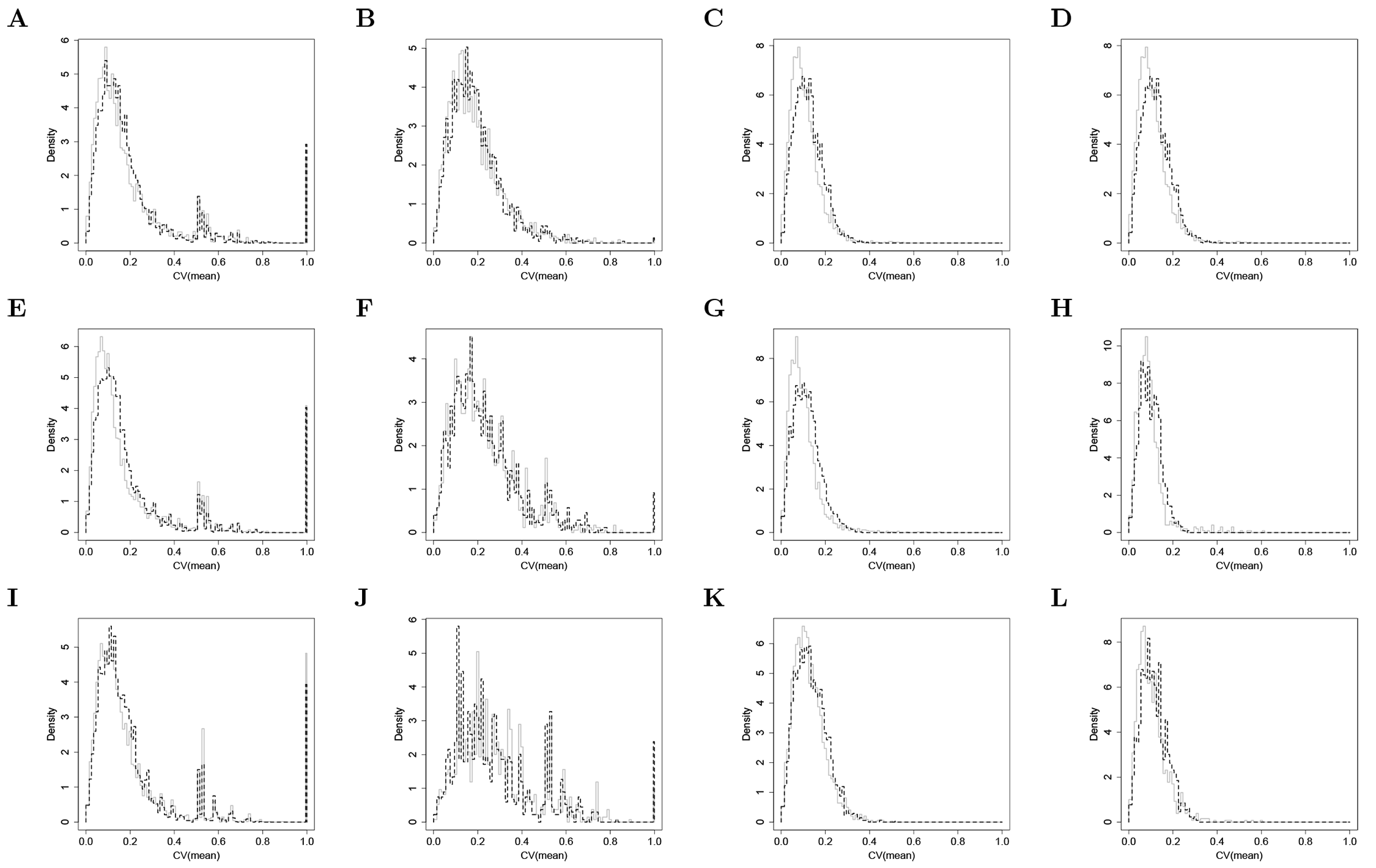

Supplement: S4 Fig — A rigorous challenge of our statistical model, with a single shape parameter a for each condition in the yeast GR study, is provided by looking at the overall distributions of CV(mean), and the distributions of CV(mean) for transcripts whose means fall into each of several subintervals of the range of mean values. (A) Histograms of sample estimates of CV(mean), plotted on a density scale, corresponding to the yeast quiescence study with C-limited growth at rate per cell of 0.12 h-1. Solid grey line correspond to experimental data (CV values computed from 3 replicates), and the dashed black lines are for corresponding synthetic (Monte Carlo) data generated according to the negative binomial model in S7 Appendix Eq (1). Mean ± sd of the distributions for lab, and synthetic data are 0.19±0.19 and 0.19±0.19, respectively. Shape parameter for synthetic data, a = 23, determined by a maximal marginal likelihood method (S6 Appendix). (B-D) CV(mean) for sample mean values in the the top 3 of 4 bins of equal width on the logarithmic scale of mean values. The spike at a CV equal to 1 corresponds to transcripts that were detected in only 1 out of 3 replicates, and it stems from those transcripts expressed at very low copy number (nominal abundance of ∼ 2 transcripts per 1000 cells). The spikes in CV values between roughly 0.5 and 1 (panels A–C) come exclusively from transcripts with very low levels of expression. (E–H) Similar to (A–E), but data are from libraries corresponding to growth rate per cell of 0.20 h-1. Mean ± sd of the full distributions for lab, and synthetic data in panel (E) are 0.19±0.22 and 0.19±0.21, respectively. Shape parameter for synthetic data, a = 34. (I–L) Similar to (A–E) but data are from libraries corresponding to growth rate per cell of 0.30 h-1. Mean ± sd of the full distributions for lab, and synthetic data in panel (E) are 0.21±0.23 and 0.20±0.21, respectively. Shape parameter for synthetic data, a = 24. (TIF) [file pcbi.1006794.s012.tif]

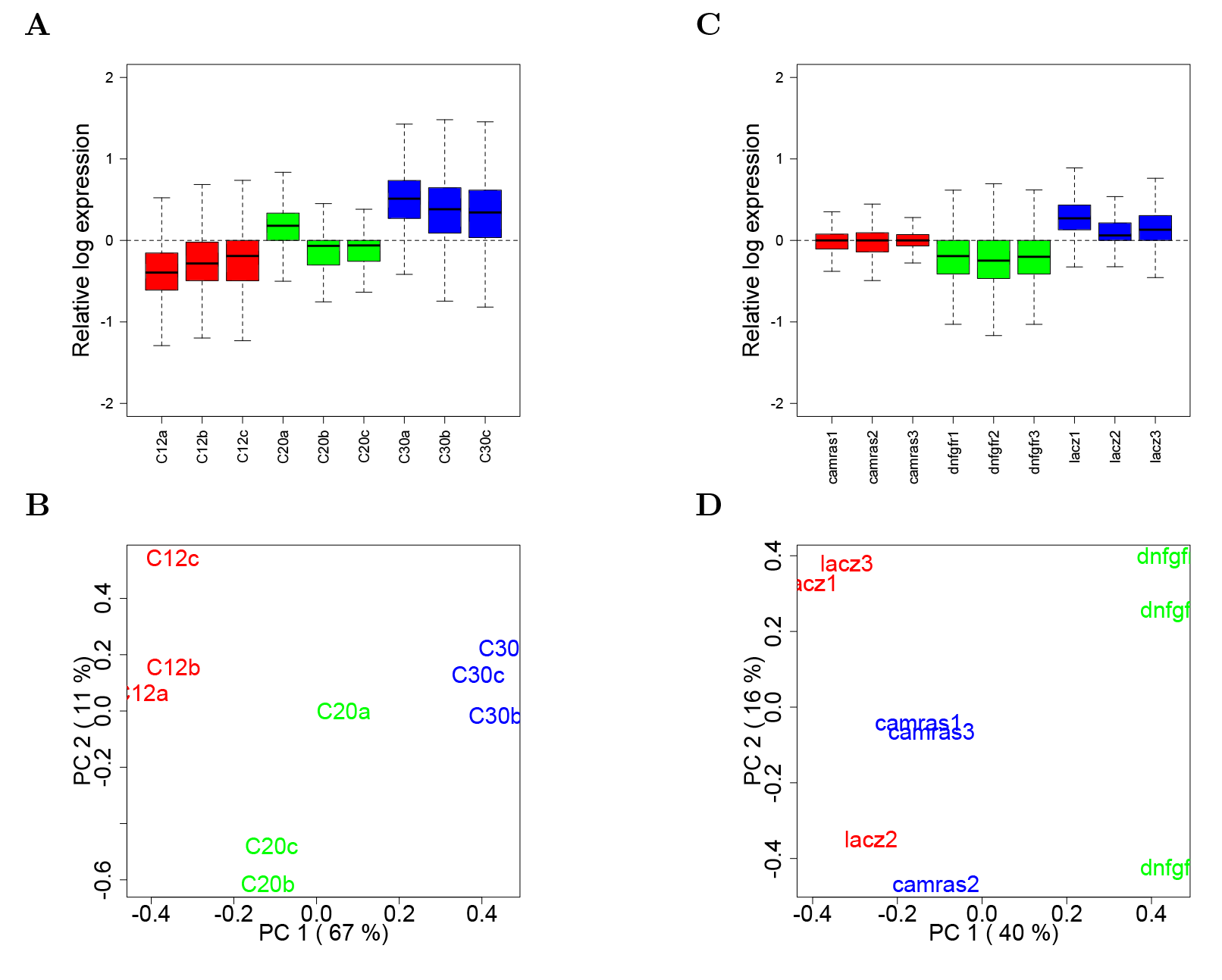

Supplement: S5 Fig — (A) Diagnostic relative log expression (RLE) plots of adjusted counts obtained by applying an RUVr normalization method based on residuals to νj-normalized counts (z abundance values) from yeast GR study (R function RUVr in the RUVSeq package [15]). Before application of RUVr, the table of counts was filtered to include only rows for those transcripts that were detected in more than 6 of 9 libraries (3 replicates for each of 3 growth rates per cell). This eliminates transcripts that were not detected at all in one or more of the 3 conditions. Next, the value 1 was added to each count, as usual, to ensure that the log of each count exists. Next, νj normalization was applied, followed by RUV normalization. These RLE plots resemble those for the νj-normalized data, followed by δj to correct for putative volume/dilution/extraction/counting errors, in S1D Fig, in the variation within condition, and the variation of the 0.5 quantile of the log relative expression distributions across conditions. In principle, the two correction methods, the RUVr method with the factor matrix W and the δ correction factor method could yield identical results in the special case with one factor of variation where W = ± log δ (S8 Appendix). We did indeed find these 2 sets of constants to be similar for our yeast data, with correlation coefficient equal to 0.83. (B) Diagnostic principal components analysis (PCA) biplot of the normalized scores of the second, versus the first principal component, corresponding the normalized counts matrix for panel for panel A. The seemingly good clustering of the libraries in this plot is confirmed by kmeans, and hierarchical clustering. Each of the 3 clusters contains libraries from only one condition. (C) Similar to panel A, but based on data from the Ciona embryonic differentiation study. These RLE plots are similar to those in S1F Fig, where the original counts were νj normalization was followed by δj normalization to correct for putative library preparat [file pcbi.1006794.s013.tif]
