# Supplementary material for: A complete statistical model for calibration of RNA-seq counts using external spike-ins and maximum likelihood theory
Source: PLoS Comput Biol. 2019 Mar 11;15(3):e1006794. doi: 10.1371/journal.pcbi.1006794 (PMC6428340; doi:10.1371/journal.pcbi.1006794)
Supplement: S1 Code and Data — The YmatRNAyeast.txt, YmatRNAciona.txt, and YmatRNAdilution.txt files are data frames for native RNA count data (genes-by-replicates) for the yeast growth rate study, Ciona embryonic differentiation study, and yeast dilution study, respectively. The corresponding count data for spike-in molecules are named similarly, but with SI instead of RNA. Data files ERCCyeast.txt and ERCCciona.txt are data frames for detected ERCC spike-in molecules in the yeast growth rate study and Ciona study, giving attomoles of each molecule added to native RNA, length (nt), GC content, and folding energy (Kcal mol-1). The ERCCdilutionNmatAttomoles.txt file is a data frame for the yeast dilution study that gives attomoles of detected spike-in molecules for the high-volume (H) and low-volume (L) spike-in aliquot conditions. (ZIP) [file pcbi.1006794.s014.zip › S1CodeandData/AthanasiadouEtAlPaperCode2_raDT.pdf]

Code and Applications to Yeast Growth Rate Study

Source all needed functions and activate needed packages

```
source('MLfitToGrowthRate.aOFl.R')
source('LLlambdaOFlEXP.R')
source('MAplotYobsYexp.R')
source('SSUandLSUrRNA.R')
colLib<-rep(c("red","green","blue"),each=3)
```

To install and activate needed Bioconductor packages, uncomment

```
#source("https://bioconductor.org/biocLite.R")
#biocLite("EDASeq") #Shouldn't need to do this again
#library(EDASeq)
#biocLite("DESeq2")
#library(DESeq2)
```

1 Analysis of differential gene expression in yeast growth rate study data

1.1 RNA and spike-in count data and spike-in molecular properties

Read in spike-in and RNA and count data frames, YmatSIyeast.txt, and YmatRNAyeast.txt, respectively. Column labels are *conditions*. Labels C12a–C12c signify 3 replicates for C-limited growth at a rate (per cell) of 0.12,h-*1*; C12 and C30 correspond to growth rates of 0.20 and 0.30,h-*1*, respectively. Row labels for the spike-in data frame are the spike-in identifiers; row label for the RNA data frame are transcript identifiers. Undetected transcripts and spike-ins have already been removed.

```
YmatSI<- as.matrix( read.table(file="YmatSIyeast.txt") )
YmatRNA<-as.matrix( read.table(file="YmatRNAyeast.txt") )
```

Read in a data frame containing the spike-in amounts (attomoles) added to each sample from which RNA was extracted. The data frame also has columns for molecular properties: length (nt), GC content, and DeltaG (Kcal/mol), the folding energy.

```
ERCC<-read.table(file="ERCCyeast.txt")
ERCC<-ERCC[rownames(YmatSI),]
```

Define a factor vector, named groupC, with levels corresponding to the condition for each column of the count matrices above.

```
groupC<-as.factor(rep( c("C12", "C20","C30"),rep(3,3) ))
```

Inspect library sizes and the ratio of total spike-in count to total RNA count (roughly 0.1–0.2).

```
LibSizeSI <- colSums(YmatSI) #spike-in library sizes
LibSizeRNA<- colSums(YmatRNA) #RNA library sizes
Ratio<-LibSizeSI/LibSizeRNA
data.frame(LibSizeSI,LibSizeRNA,Ratio)
```

```
##      LibSizeSI LibSizeRNA      Ratio
## C12a    411491    1871150 0.21991342
## C12b    404867    2171203 0.18647128
## C12c    417375    2570950 0.16234271
## C20a    259158    2567382 0.10094252
## C20b    194145    1755252 0.11060805
## C20c    237054    1748464 0.13557843
## C30a    174424    1992288 0.08754959
## C30b    133603    1935219 0.06903766
## C30c    168501    1591541 0.10587286
```

1.2 Computation of maximum likelihood ν_j calibration factors, and normalization counts

For sake of convenience, and later use, extract a vector of attomoles for each spike-in, and compute corresponding molecules per cell, given that there are 10^7 cells in this study. Also compute conversion factor to convert from attomoles to molecules per cell.

```
Nvec<- ERCC[,"attomoles"] #total amol of each spike-in
                          #in sample to be sequenced
names(Nvec)<-rownames(ERCC)
AttomoleToMoleculesPerCell<-(1.e-18)*(6.22e23)*(1.e-7)
MoleculesPerCell<-Nvec*AttomoleToMoleculesPerCell
names(MoleculesPerCell)<-names(Nvec)
```

Compute spike-in proportions. Choose as the reference spike-in, the one with the largest proportion; record its proportion and attomoles; and compute the library-specific ν_j calibration factors:

```
f.vec<-rowSums(YmatSI)/sum(YmatSI) #fraction of total spike-in
                        #counts accounted for by each spike-in
```

```
INDmax<-which(f.vec==max(f.vec)) #index of spike-in
                      #with largest proportion, to be used as
                      #the reference spike-in
f.ref<-print(f.vec[INDmax]) #fraction (empirical proportion) for reference
```

```
## ERCC-00130
##  0.2716534
```

```
n.ref<-print( Nvec[names(f.vec[INDmax])] ) #corresponding amol
```

```
## ERCC-00130
##       3000
```

```
nu <- (f.ref/n.ref)*LibSizeSI #for counts to nominal amol conversion
Zmat<-YmatRNA%*%diag(1/nu) #normalization giving  amol
colnames(Zmat)<-colnames(YmatRNA)
```

1.3 Realtive yield coefficients, α_i

Relative yield coefficients for the spike-ins are given by:

```
alphas<-(n.ref/Nvec)*(f.vec/f.ref)
```

1.4 To compare observed spike-in counts to those expected according to the multinomial model in MA plot-like format:

```
DifferenceMeasures<-MAplotYobsYexp(YmatSI,f.vec,ylims=c(-4,4))
```

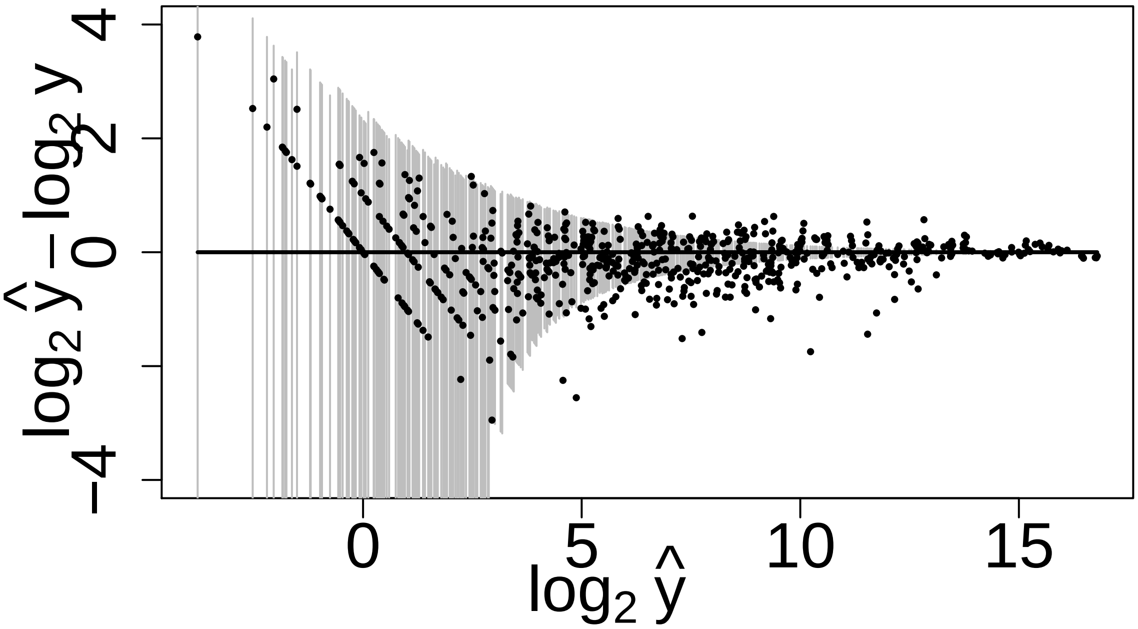


```
DifferenceMeasures
```

```
## $MeanLog2FoldDiff
## [1] -0.05595079
##
## $MeanFoldDiff
## [1] 0.9619603
```

1.5 Diagnostic RLE plots and simple correction for unwanted variation

For for purposes of RLE (relative log error) plots and removal of unwanted variation (library preparation errors), filter count matrix to ensure that each transcript was detected in all conditions. Add 1 to count matrix to be log transformed.

```
Jfilter <- apply(YmatRNA, 1, function(x) length(which(x>0))>6)
YmatRNAplus1<-YmatRNA[Jfilter,] + 1
ZmatPlus1<-YmatRNAplus1%*%diag(1/nu)
colnames(ZmatPlus1)<-colnames(Zmat)
```

1.5.1 RLE plots before correcting for unwanted variation

```
plotRLE(ZmatPlus1, col=colLib, outline=FALSE, ylim=c(-2, 2),
                                          #From the EDASeq package
mtext("Relative Log Expression", cex=1.5, side=2, line=2.6)
```

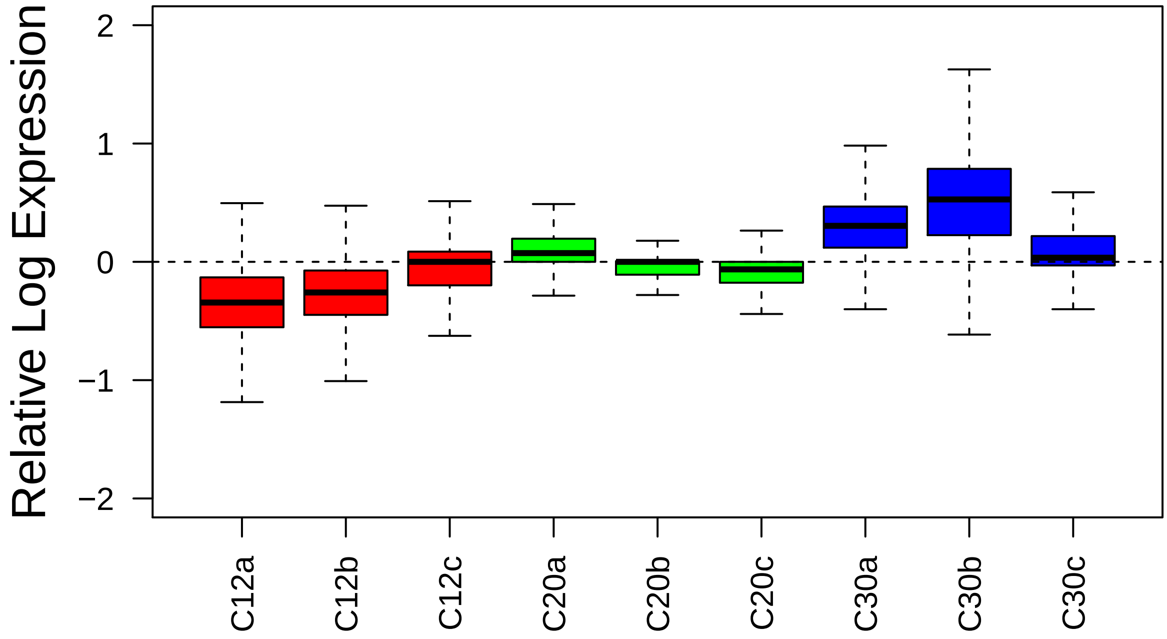

1.5.2 Compute δ_j correction factors to account for library preparation errors and apply to ν_j normalized abundances

```
meanLogZplus1<-t(apply(log(ZmatPlus1), 1,
                       function(x) tapply(x,groupC,mean)))
MeanMatLogZplus1<-t(apply(meanLogZplus1,1,function(x) rep(x,each=3)))
aux<-colMeans(log(ZmatPlus1) - MeanMatLogZplus1)
delta<-exp(aux) #The correction factors
Zmat<-Zmat%*%diag(1/delta)
colnames(Zmat)<-colnames(YmatRNA)
```

1.5.3 RLE plots for adjusted abundance values, counts normalized by $(\nu_j\delta_j)$

```
ZmatPlus1Corrected<-ZmatPlus1%*%diag(1/delta)
colnames(ZmatPlus1Corrected)<-colnames(Zmat)
plotRLE(ZmatPlus1Corrected, col=colLib, outline=FALSE, ylim=c(-2, 2), las=2) #From the 
mtext("Relative Log Expression", cex=1.5, side=2, line=2.6)
```

1.6 Hypothesis testing for dependence of abundance on growth rate

Maximum likelihood fit of a model in which abundance depends exponentialy on GR. The function *MLfitToGrowthRate.aOFl* below maximizes the sum over replicates of the log

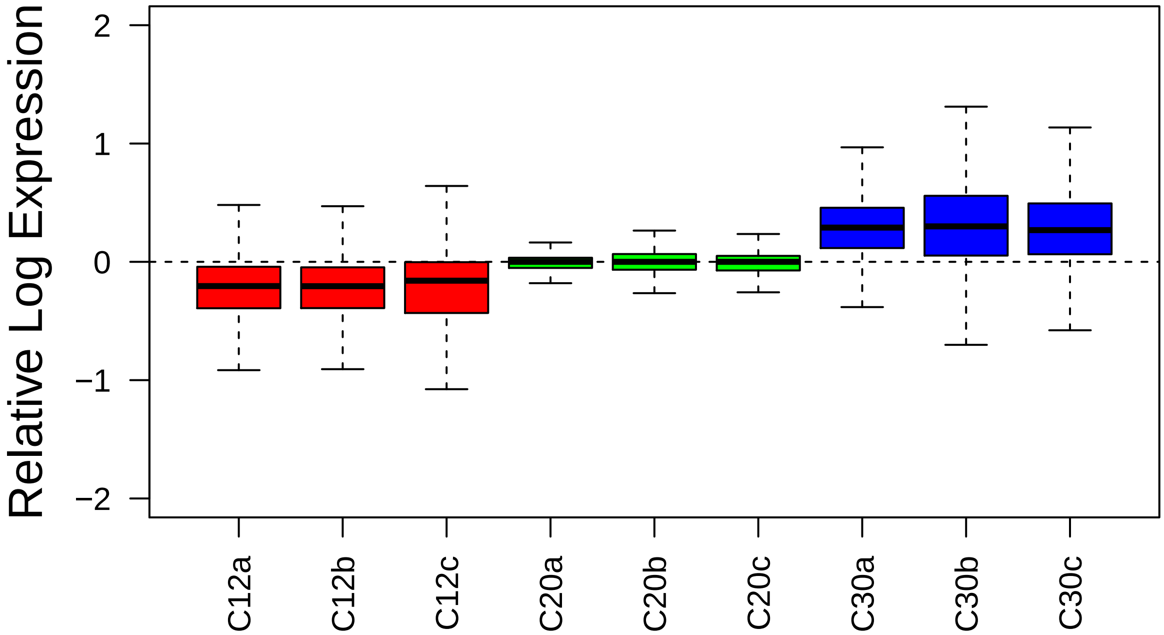

Figure 1: RLE plot RNA abundances after removal of unwanted variarion.

likelihoods (same as probabilities) of the observed counts under H_0: no dependence of abundance on growth rate; and under under H_A: abundance depends exponentially on growth rate. The test satistic is the standard log of the ratio of maximum likelihoods test. The output of *MLfitToGrowthRate.aOFl*, mlfit.out is a list: mlfit.out$pValML gives the *p*values, and mlfit.out$EstA gives the vector of sample estimates of (θ_0, θ_1) in Eq~S28, under H_A.

The maximum likelihood fit is done under the assumption of fixed negative binomial shape parameter a for each condition. The a-value for each library is comupted by maximizing over a the marginal likelihood of all observed counts within each condition, as described in the text. The function below that does this can be run by supplying to the function, as inputs, the matrix of RNA counts, YmatRNA; the 3-column matrix of mean abundances (one column for all the transcripts in each of 3 conditions), meanZ; and a vector of length 3 for the inial guesses of the 3 shape parameters (a-values), $a0.vec$, e.g., a0.vec<-c(20,20,20).

```r
meanZ<-t(apply(Zmat, 1,function(x) tapply(x,groupC,mean)))
a.vec<-c(23,34,24) #Marginal maximum likelihood values for
           #shape parameters for the 3 yeast growth rates
           #S7 Appendix
```

```r
lambda<-rep(c(0.12,0.2,0.3),each=3) #Growth rate (GR)
                                    #in divisions per hr
mlfit.out<-MLfitToGrowthRate.aOFl(Y=YmatRNA,
            norm.const=delta*nu,Z=Zmat,
                lambda=lambda,a.vec=a.vec,print.level=0)
pVal<-mlfit.out$pValML #p-values for H0: exponential
                       #constant is equal to zero
q<-p.adjust(mlfit.out$pValML, method="BH") #adjusted p-values
FDR<-0.01 #False discivery rate
sum(q<FDR) #Number of transcripts significantly regulated by GR
```

```
## [1] 4302
```

```r
phi.sig<-mlfit.out$EstA[q<FDR,] #significant exponential growth
                                #rate coefficients
phi_1.sig<-mlfit.out$EstA[q<FDR,2]
```

1.6.1 Histogram of exponential constants in model for dependence of abundance on growth rate, Fig 2

```r
hist(phi_1.sig,breaks=500,freq=FALSE,cex.lab=2,
     cex.axis=2, xlab=NA, ylab=NA, xlim=c(-30,30))
mtext(expression(paste(phi[1],' (h)')),side=1,line=2.5,cex=2,
      adj=0.515)
mtext("Density", side=2, line=2.5, cex=2)
```

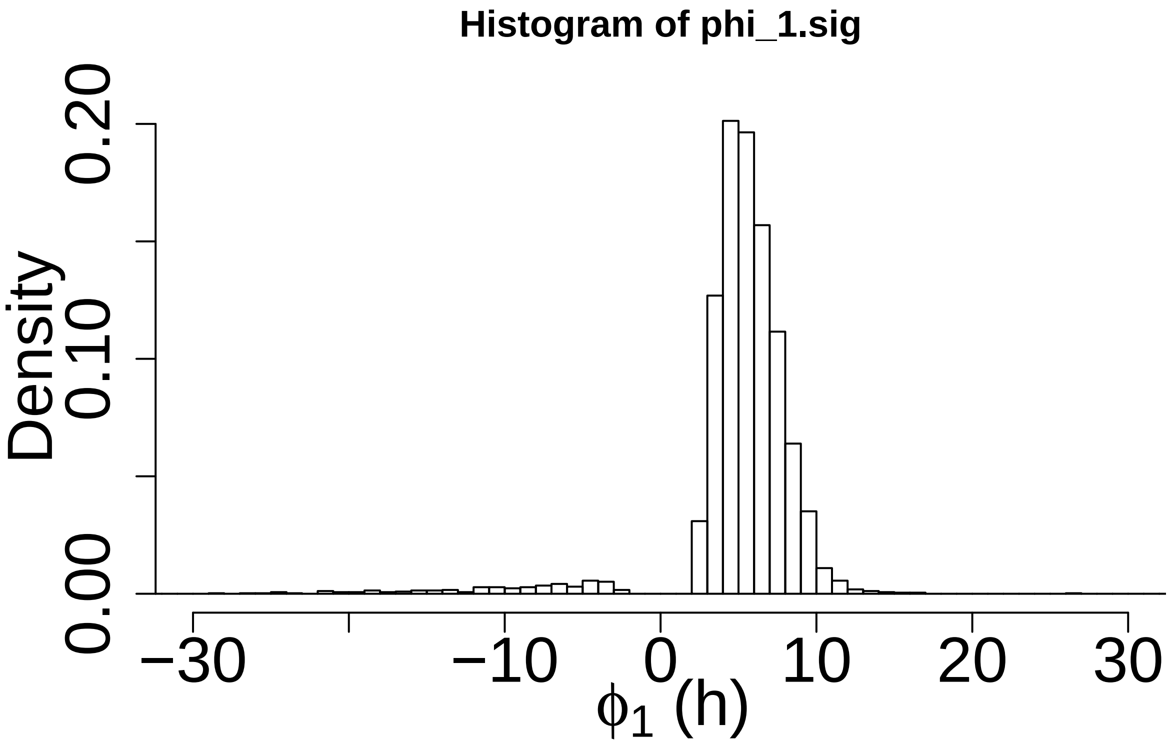

1.6.2 Regulation of Ribosomal RNA, SSU rRNA and LSU rRNA

To plot experimentally measured log abundance versus growth rate and compare to that predicted for the model:

```
SSUandLSUrRNA(phi_1.sig, Zmat)
```

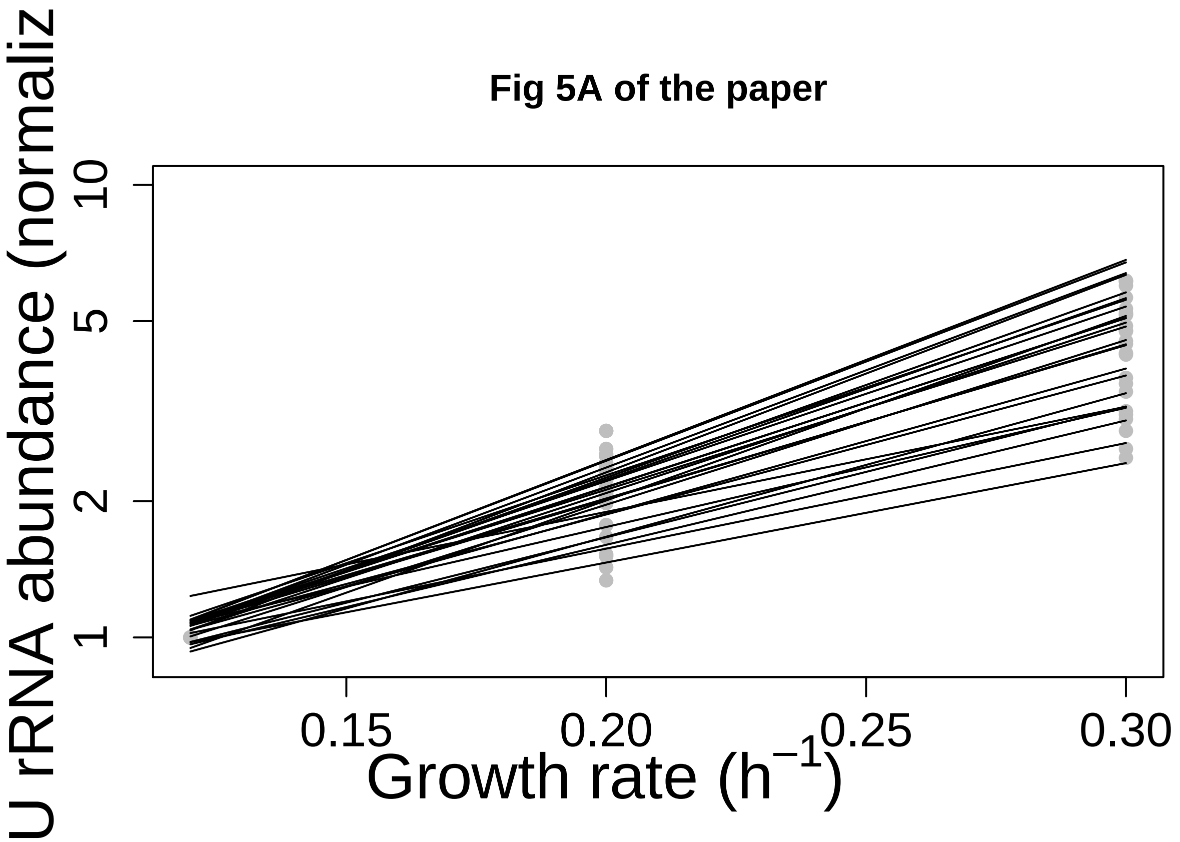
Fig 5A of the paper
SU rRNA abundance (normaliz
1
2
5
10
0.15
0.20
0.25
0.30
Growth rate (h^{-1})

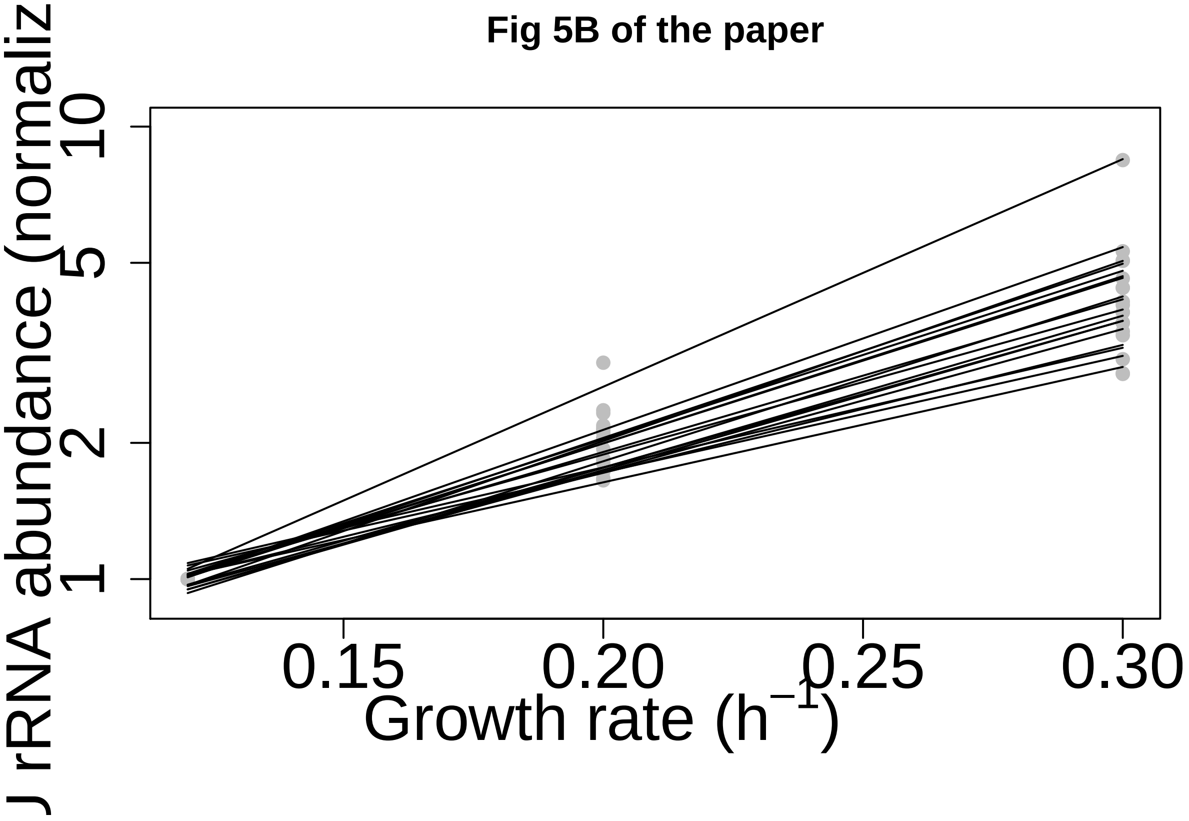
Fig 5B of the paper
J rRNA abundance (normalize
1
2
5
10
0.15
0.20
0.25
0.30
Growth rate (h^{-1})
